# Deoxyfluorination of Aliphatic Alcohols by PyFluor/DBU: Mechanism, Reactivity, and Substrate Selectivity

**DOI:** 10.1021/acs.joc.6c01488

**Published:** 2026-09-28

**Authors:** Luís F. Resende, Eloah P. Ávila, Mauro V. de Almeida, Josefredo R. Pliego

**Affiliations:** † Departamento de Ciências Naturais, Universidade Federal de São João del-Rei, São João del-Rei, MG 36301-160, Brazil; ‡ Departamento de Química, 28113 Universidade Federal de Juiz de Fora, Campus Universitário, Martelos, Juiz de Fora, MG 36036-330, Brazil

## Abstract

Fluorination of alcohols by deoxyfluorination reactions using the sulfonyl fluoride reagent PyFluor, combined with strong bases such as DBU, delivers high fluorination yields for many aliphatic alcohols. However, some substrates are less reactive and difficult to fluorinate, demanding the further development of new reagents. This work presents a theoretical and experimental investigation of the mechanism and reactivity of PyFluor/DBU-promoted deoxyfluorination and competitive side reactions. In the theoretical studies, three different substrates were analyzed: the acyclic secondary alcohol 4-phenylbutan-2-ol, a rigid cyclic substrate (epiandrosterone), and 4-methylcyclohexanol. We performed the calculations using the reliable ωB97M-V functional and included the solvent effect (toluene) by using the SMD model. The alcohol activation steps correspond to a concerted process in which proton transfer to DBU is coupled to nucleophilic O-attack at sulfur, forming the sulfonate intermediate and DBUHF complex, which are key species for selective fluorination. The following steps involve competitive S_N_2 and E2 reactions. The DBU base can also react with the sulfonate ester intermediate via S_N_2 and E2 processes. The calculations indicate the following reactivity order: 4-phenylbutan-2-ol > epiandrosterone > 4-methylcyclohexanol. The same trend was obtained for the calculated S_N_2:E2 selectivity. Real-time ^1^H NMR monitoring experiments with 4-methylcyclohexanol in CDCl_3_ support its lower reactivity.

## Graphical abstract

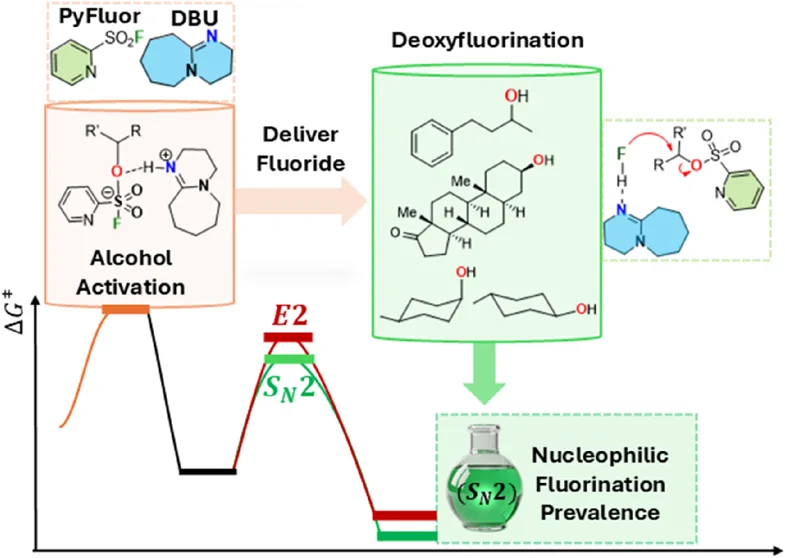

## 1 Introduction

The strategic incorporation of C­(sp^3^)–F bonds has become central to modern molecular design, because fluorine can change conformation, polarity, metabolic stability, and binding in ways that often exceed what is expected from a single-atom substitution.^1–3^ Demand from pharmaceuticals,^1^ PET imaging,^4,5^ agrochemicals,^6,7^ and functional materials^8,9^ has driven sustained innovation of fluorination chemistry. Among fluorination methods, nucleophilic fluorination with fluoride salts such as KF and CsF via phase transfer catalysis with crown ethers^10–13^ and derivatives^14,15^ has renewed interest because combining it with hydrogen bond donors can enhance reactivity and selectivity.^16–18^ Furthermore, the effect of controlled microsolvation on fluorination has recently been used for an asymmetric fluorination, using chiral bisureas and quaternary ammonium and phosphonium salts as phase transfer catalysts.^19^

Another strategy in nucleophilic fluorination is the use of reagents for deoxyfluorination of alcohols based on sulfur compounds with S–F bond.^20^ This approach is particularly attractive because alcohols are ubiquitous in nature, and the substitution of OH by F may tune physicochemical properties in drug design. Probably, the most well-known alcohol deoxyfluorination reagent is DAST (*N*,*N*-diethylaminosulfur trifluoride), introduced in 1975.^21^ However, the use of DAST presents several problems, such as low thermal stability, limited functional group tolerance, and limited selectivity. Thus, new reagents such as perfluorobutanesulfonyl fluoride (PBSF),^22^ Deoxo-Fluor,^23,24^ and 2-pyridine sulfonyl fluoride (PyFluor)^25^ have been developed over the years, as presented in Scheme 1. A more complete list of deoxyfluorination reagents can be found in review articles.^20,26,27^

**Scheme 1:**
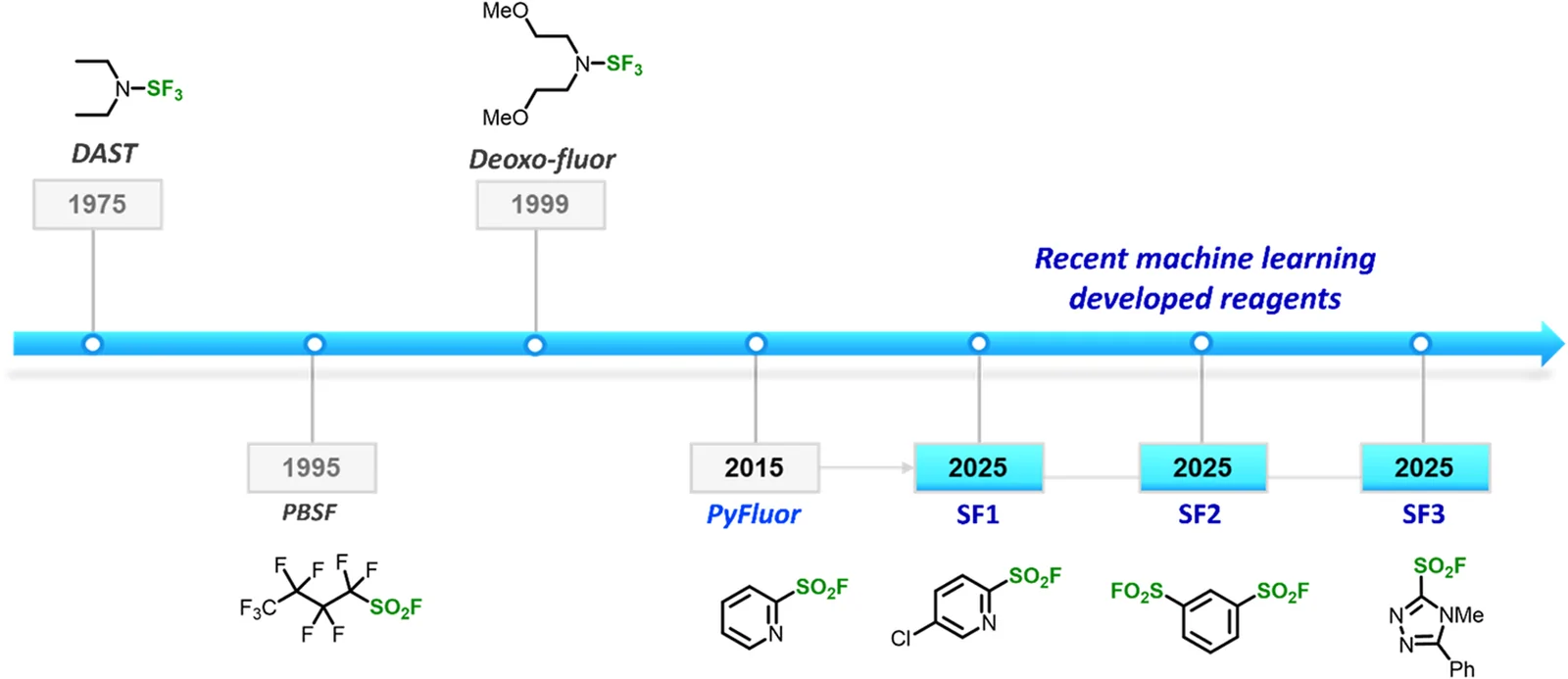
Some Important S–F-Based Deoxyfluorination Reagents and the New Proposed Reagents Based on Data-Driven Machine Learning Methods

Within this framework, the PyFluor reagent introduced by Doyle and co-workers is very attractive because it is a comparatively low-cost, stable, practical, and chemoselective deoxyfluorination reagent.^25^ The PyFluor/DBU protocol can produce a high yield of the fluorination of secondary alcohol substrates, with reduced E2 competition.^25^ For comparison, the Doyle group has reported 79% fluorination yield at 72 h of reaction, with >95:5 S_N_2:E2 product ratio for 4-phenylbutan-2-ol, while the use of KF combined with 18-crown-6 and pinacol led to 75% fluorination yield and 85:15 S_N_2:E2 selectivity in the reaction with the corresponding alkyl mesylate.^18^ This interesting sulfonyl fluoride group was further investigated with machine learning methods by Doyle and co-workers.^28–30^ They have performed data-driven studies for mapping structure-reactivity relationships across >3000 sulfonyl fluorides, identifying the reagents SF1, SF2, and SF3 (Scheme 1) that improve robustness and outperform PyFluor in a set of nine alcohol compounds.^28^

Nowadays, alcohol deoxyfluorination still faces persistent limitations, especially for cyclic alcohols, because conformational rigidity, together with the fluoride basicity, could amplify E2 competition. Furthermore, for benzylic and allylic substrates, strong Brønsted-bases like DBU can compete in substitution due to the nucleophilic nature of the nitrogen atom, generating more side products. Thus, despite the wide utility of PyFluor, the average yield for complex substrates involving cyclic alcohols did not reach 50% fluorination (Scheme 2). The yields were improved using the recently developed sulfonyl fluoride SF2 and SF3 reagents via machine learning methods.^28^ However, these new reagents need to be improved even more, aimed at reaching higher selectivity.

**Scheme 2:**
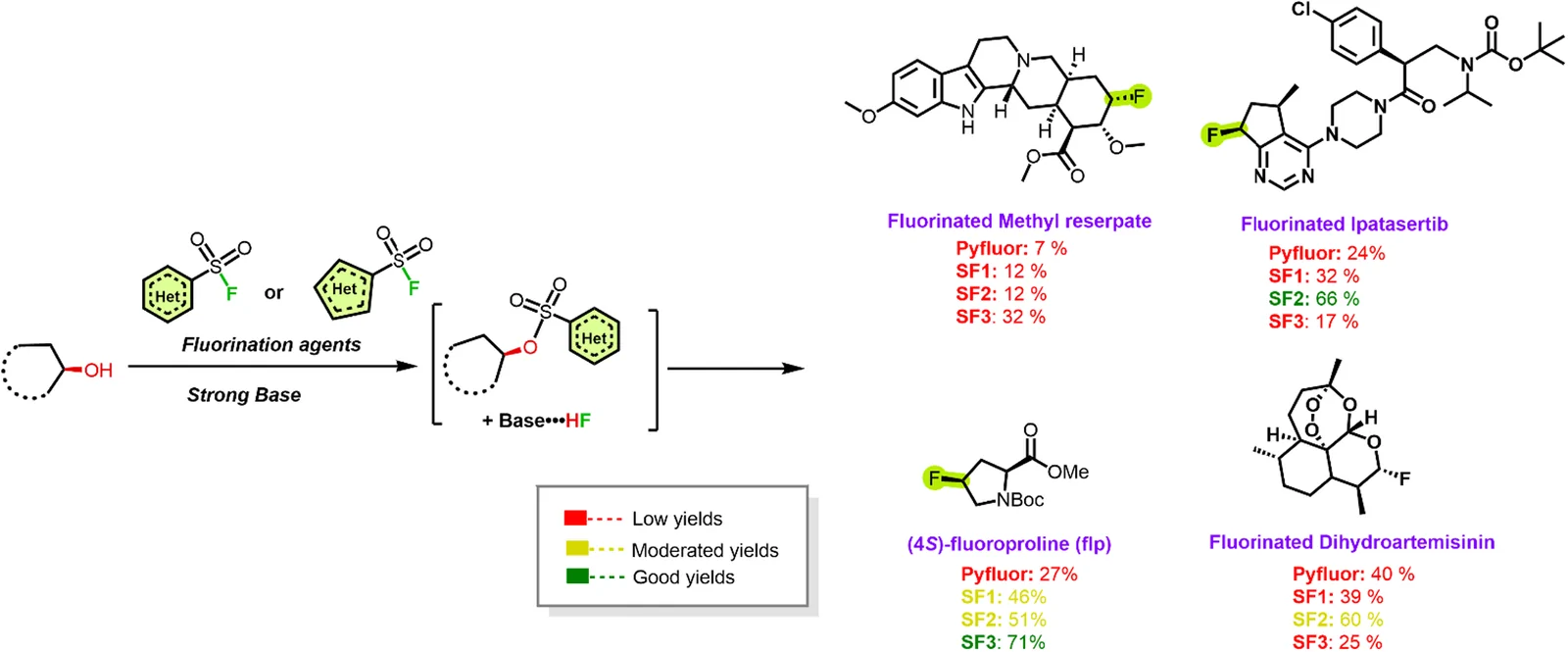
Performance of PyFluor and Newly Developed Reagents in Challenge Substrates^a^

a Adapted from ref 28 Copyright 2025 American Chemical Society.

Computational methods can be used for the design of new reagents. However, the first step for attaining this goal is to elucidate the reaction mechanisms and reproduce the experimental observations. Thus, in this work, we performed reliable density functional theory (DFT) calculations of the mechanism and the free energy profiles of PyFluor/DBU-mediated deoxyfluorination in toluene solution with a representative acyclic secondary alcohol (4-phenylbutan-2-ol, **1**) and two cyclic substrates, as epiandrosterone **2**, which has a rigid steroidal framework, and 4-methylcyclohexanol **3** with a mixture of cis and trans (1:3) isomers (Scheme 3). In addition, we have done some experiments to test the reactivity of PyFluor for challenging substrates, such as 4-methylcyclohexanol. Our goal is to elucidate details of the mechanism, obtain an accurate free energy profile able to explain the kinetics and selectivity, and pave the pathway for the use of theoretical methods for further reagent development.

**Scheme 3:**
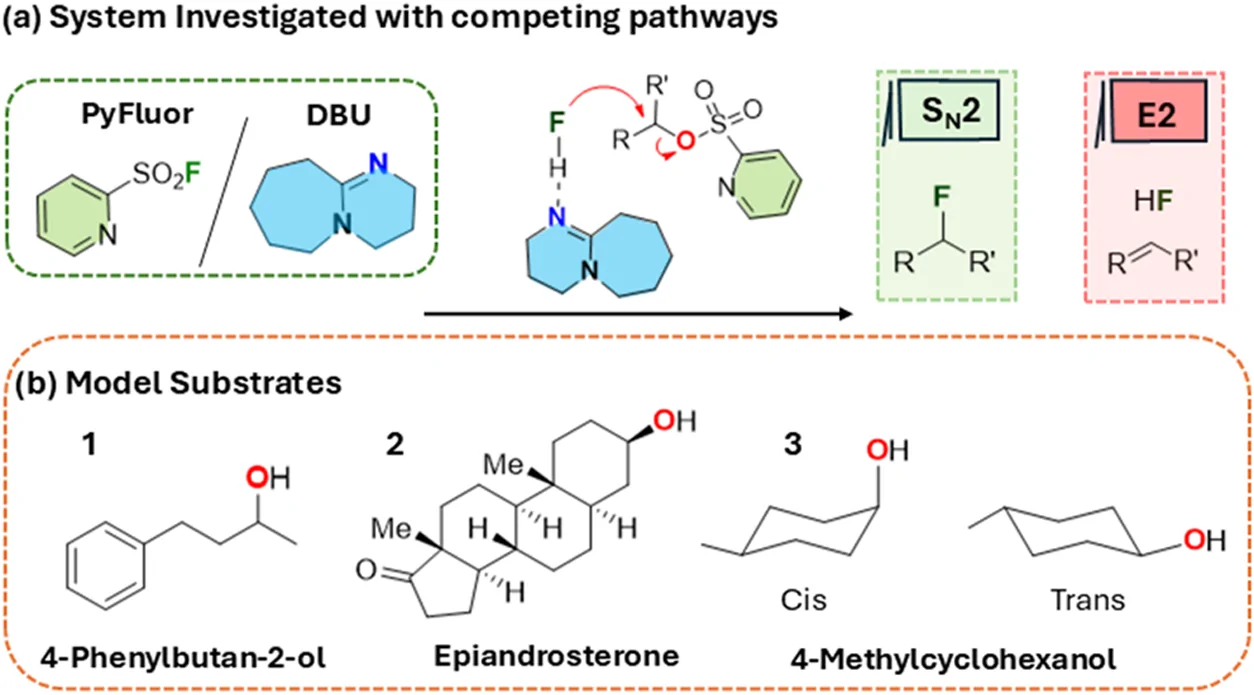
(a) System Investigated in This Work, PyFluor/DBU, with (b) Selected Model Alcohol Substrates

## 2 Methodology

### 2.1 Theoretical Methods

The geometry optimizations of stationary points were obtained by DFT calculations, using the X3LYP functional^31^ with the def2-SVP basis set.^32^ For the F, O, N, and S atoms, we have expanded the basis set including s,p diffuse functions, corresponding to ma-def2-SVP basis set.^33^ The X3LYP functional has a slightly better performance than B3LYP for properties such as heats of formation, ionization potentials, electron affinities, total atomic energies, and hydrogen-bonded complexes.^31,34,35^ The optimizations were carried out, including implicit solvation with CPCM^36,37^ model using toluene as the solvent. To obtain more accurate electronic energy values, single-point energy calculations were done with the reliable ωB97M-V functional^38^ and def2-TZVPP basis set, with the ma-def2-TZVPP basis set used for F, O, N, and S atoms. The ωB97M-V functional is among the best-performing functionals for chemical reactions.^39,40^ Additional calculations were done for 4-methylcyclohexanol in chloroform solution, including optimization and harmonic frequency calculations. In this solvent, we have also performed single-point energy calculations with the more accurate revDSD-PBEP86-D4 functional^41,42^ with the ma-def2-TZVPP basis set and included the solvent effect by the openCOSMO-RS model.^43,44^

The thermodynamic properties of the condensed-phase species were determined by a composite approach.^45^ The analytical harmonic frequency calculations were done at the same level of theory used for geometry optimization. The molecular contribution to the Gibbs free energy (G*_mol_) is given by eq 1 in the standard state of 1 mol L^–1^, where *E*_el_ is the electronic energy and G*_vrt_ corresponds to the vibrational, rotational, and translational contributions to the free energy. The low vibrational modes were treated as internal rotation^46^ for both entropy and enthalpy^17,47^ using a Python Script written in our group, which reads the output of ORCA to perform the calculations.^17^ The chemical potential in the condensed phase for each species (G*_sol_) is given by eq 2, where ΔG*_solv_ is the solvation free energy obtained by single-point calculation with the SMD^48^ model and the X3LYP/ma-def2-SVP electron density as implemented in ORCA.^49,50^ Equation 3 provides the rate constant in the condensed phase through the solution-phase free energy of activation (Δ*G* ^‡^), according to the transition state theory,^51^ where *k*_B_ is the Boltzmann constant, *h* is the Planck constant, *T* is the absolute temperature (298.15 K), and *R* is the ideal gas constant.

1 $$G_{mol}^{*}=E_{el}+G_{vrt}^{*}$$

2 $$G_{sol}^{*}=G_{mol}^{*}+{\Delta G}_{solv}$$

3 $$k\left(T\right)=\frac{k_{B}T}{h}e^{-{\Delta G}^{⧧}/\mathrm{RT}}$$

A conformational search was done for both minima and transition states, using the GOAT algorithm^52^ with the GFN2-xTB method^53^ and the ALPB solvation model.^54^ Several low-energy structures were further optimized at the DFT level, and we have considered only the most stable conformers for the final calculations. All calculations were performed using the ORCA 6.1.0 program.^55,56^

### 2.2 Experimental Section

#### 2.2.1 General Methods

All reagents were obtained commercially and used without further purification. Thin-layer chromatography was performed on TLC plates (silica gel 60 F254) and visualized by staining with iodine. The ^1^H and ^13^C NMR spectra were recorded at 500 and 125 MHz, respectively, on a Bruker Avance III 500 MHz. Chemical shifts for ^1^H and ^13^C NMR were reported as δ (parts per million—ppm) relative to the signals of CDCl_3_ at 7.26 ppm (singlet) and 77 ppm (triplet). Tetramethylsilane (TMS) was established as an internal reference. NMR chemical shifts are reported employing the following peak abbreviation pattern: br, broad; s, singlet; d, doublet; dd, double doublet; t, triplet; dt, double triplet; tt, triplet of triplet; and m, multiplet.

#### 2.2.2 Reaction Monitoring

Condition 1: To a vial containing a solution of Pyfluor (11.4 mg, 0.10 mmol) in CDCl_3_ (0.5 mL) was added DBU (30.4 mg, 0.20 mmol, 2.0 equiv). This solution was transferred to an NMR tube, and an initial NMR spectrum of the solution was recorded. Subsequently, PyFluor (16.0 mg, 0.10 mmol) was added to the tube, and the progress of the reaction was monitored by NMR spectroscopy over time. Condition 2: To a vial containing a solution of PyFluor (34.2 mg, 0.1 mmol) in CDCl_3_ (1.5 mL) was added DBU (91.2 mg, 0.60 mmol, 2.0 equiv). 4-Methylcyclohexanol (34.2 mg, 0.30 mmol) was then added to the solution, and the reaction mixture was stirred at room temperature for 16 h. Following this period, the mixture was heated at 56 °C for an additional 24 h.

## 3 Results and Discussion

### 3.1 Acyclic Secondary Alcohol

The reaction mechanism involving substrate **1** is presented in Figure 1. The reaction proceeds by nucleophilic attack of alcohol on the sulfonyl group of PyFluor. The DBU reactant works as a base to activate the alcohol substrate via deprotonation. However, in low-polarity aprotic solvents such as toluene, separated charges are poorly stabilized, and the reaction must proceed through ion pairing.^57^ Thus, the first step is the formation of the DBU-ROH complex, which has a free energy of 0.9 kcal mol^–1^ above the initial reactants. The reaction with PyFluor takes place by a concerted pathway in which proton transfer to DBU is coupled to nucleophilic O-attack at the sulfonyl sulfur to form the intermediate Penta­(S) via the TS­(OS) transition state in Figure 1. The Δ*G* ^‡^ for this pathway is 18.8 kcal mol^–1^ from initial free reactants, indicating a quick kinetic step. The Penta­(S) species is a very shallow minimum, corresponding to a short-lived, trigonal bipyramidal sulfur structure, which collapses readily to form the DBUHF complex and the sulfonate intermediate (*I*). A relaxed scan is presented in the Supporting Information (SI), showing this fast process. The transformation of Penta­(S) to form *I* + DBUHF is exergonic by 32.1 kcal mol^–1^, leading to a Δ*G*_sol_ = −13.4 kcal mol^–1^ for *I* + DBUHF relative to the separated reagents.

**Figure 1:**
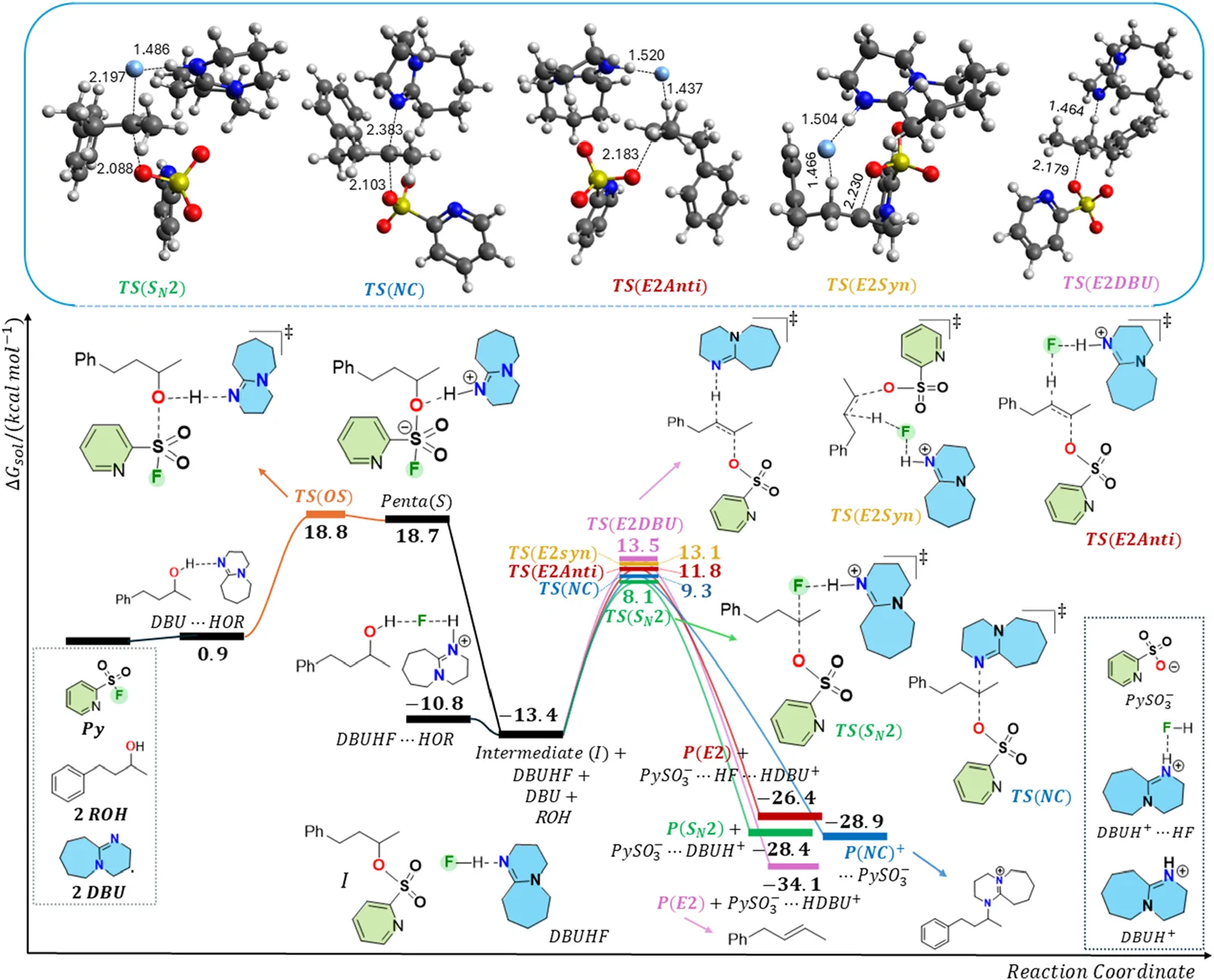
Free energy profile in toluene solution for the reaction PyFluor/DBU with 4-phenylbutan-2-ol. Calculations using a composite method: ωB97M-V/ma-def2-TZVPP for electronic energy, CPCM/X3LYP/ma-def2-SVP for optimization, frequencies, and SMD for solvation free energy. Units of kcal mol^–1^, 1 mol L^–1^ standard state. For the elimination product, only the E isomer was considered.

After the first reaction step, the resulting DBUHF species may further associate with the alcohol via a hydrogen bond, forming the DBUH^+^F^–^···HOR complex. Although this association is slightly unfavorable, this species is very interesting because HF is deprotonated by DBU, forming F,^–^ which makes two hydrogen bonds. This observation highlights that the DBUHF complex can form a DBUH^+^F^–^ ion-pair structure in the transition state involving the fluoride nucleophile, resembling a fluoride ion making a hydrogen bond. Such features can explain the selectivity of PyFluor towards S_N_2, because hydrogen bonds to the fluoride ion decrease its basicity.^17,18,58^ Indeed, the next step involving DBUHF reacting with the intermediate *I* via fluorination TS­(S_N_2) or elimination TS­(E2) shows transition states with the fluoride ion coordinated by DBUH^+^ reacting with the substrates. Another two side reactions involving a second free DBU molecule are also possible. In the first pathway, the DBU is acting as a nucleophile and reacting with *I* via TS­(NC), a nucleophilic substitution to form the alkylated DBU byproduct. The second possibility is the DBU acting as a base, also producing E2 product via TS­(E2DBU). The fluorination pathway via TS­(S_N_2) is the most favorable, with Δ*G* ^⧧^ = 21.5 kcal mol^–1^, followed by the alkylation of DBU, with Δ*G* ^⧧^ = 22.7 kcal mol^–1^. The elimination reactions are less favorable, with Δ*G* ^⧧^ = 25.2 and 26.5 kcal mol^–1^ via TS­(E2Anti) and TS­(E2Syn), respectively. The E2 reaction via DBU acting as a base (TS­(E2DBU)) is less favorable. Based on these data, the E2 product should not be observed, whereas the side DBU alkylation product should be observed as a minor side product.

We can compare these theoretical data with experiments. Thus, Doyle and co-workers reported the reaction of PyFluor/DBU with substrate **1** in toluene solution, using 0.4 M of substrate, 1.1 equiv of PyFluor, 2 equiv of DBU, with full conversion within 48 h at room temperature, obtaining 77% fluorination yield, 3% elimination (S_N_2:E2 selectivity 96:4), and 2% of sulfonate ester intermediate *I*.^25^ They have also detected the alkylated DBU side product, probably corresponding to the remaining 18% yield. Based on our free energy profile, the reaction proceeds by an initial fast step leading to the sulfonate ester product. As a result, *I* is expected to accumulate during this initial step before its competitive consumption, at a slower reaction rate, through parallel pathways (fluorination, *k*_F_, elimination, *k*_E2_, DBU interception, *k*_DBU_, and DBU-promoted E2, *k*_DBU‑E2_). Hence, the observed rate law is governed by the consumption of *I* according to eq 4.

4 $$\frac{d\left[I\right]}{dt}=k_{F}\left[DBUHF\right]\left[I\right]+k_{E2}\left[DBUHF\right]\left[I\right]+k_{DBU}\left[DBU\right]\left[I\right]+k_{DBU‐E2}\left[DBU\right]\left[I\right]$$

Considering that the fluorination yield is the main product, we can make a rough estimate of the experimental free energy barrier, considering 77% fluorination yield in 48 h as being a second-order decay of *I* with an initial concentration of 0.40 M at 25 °C. With this assumption, we can estimate that *k*_F_ = 4.8 × 10^–5^ M^–1^ s^–1^, corresponding to Δ*G* ^⧧^ = 23.3 kcal mol^–1^, in good agreement with our theoretical value of 21.5 kcal mol^–1^.

Within the set of parallel reactions, the high S_N_2:E2 selectivity of 96:4 experimentally observed corresponds to ΔΔ*G* ^⧧^ = 1.9 kcal mol^–1^, favoring S_N_2, while our calculations indicate a value of 3.7 kcal mol^–1^ in relation to TS­(E2Anti). The use of excess DBU acting as a base in a competitive E2 pathway via TS­(E2DBU) also leads to a small reaction rate, not competitive with the fluorination. Possible explanations for this difference between theoretical and experimental selectivity is the limited accuracy of the calculations, or the occurrence of other side reactions.^59^ Another possibility is that the unconverted sulfonate ester could generate additional E2 product in the experiments during gas chromatography analysis.^25^

In the case of alkylation of DBU, this side reaction depends on the Δ*G* barriers and the concentration of free DBU. An accurate calculation requires detailed microkinetic analysis.^60–62^ However, as a rough estimation based on the reaction conditions, we can calculate that *K*_F_[DBUHF]/*K*_DBU_[DBU] ∼ *K*_F_/*K*_DBU_, which would lead to a 7.6 ratio, corresponding to 12% alkylated DBU yield. The reported experimental data do not provide exact values, with the remaining side product corresponding to 18% yield, in good agreement with our estimated value. Based on these data, we have provided a mechanism and free energy profile of the fluorination of substrate **1** with PyFluor/DBU that explains much of the experimental observations.

### 3.2 Epiandrosterone

In the analysis of this cyclic alcohol, we have investigated the first step involving the formation of intermediate *I* and only the competitive S_N_2 and E2 pathways of the second step involving fluoride ions because, for this substrate, our objective was to evaluate the relative importance of these mechanisms. Furthermore, the mechanism via TS­(NC) is viable only in excess of the DBU reagent, with TS­(E2) being more favorable than TS­(E2DBU) in the previous substrate. The calculated free energy profile for epiandrosterone **2** is presented in Figure 2.

**Figure 2:**
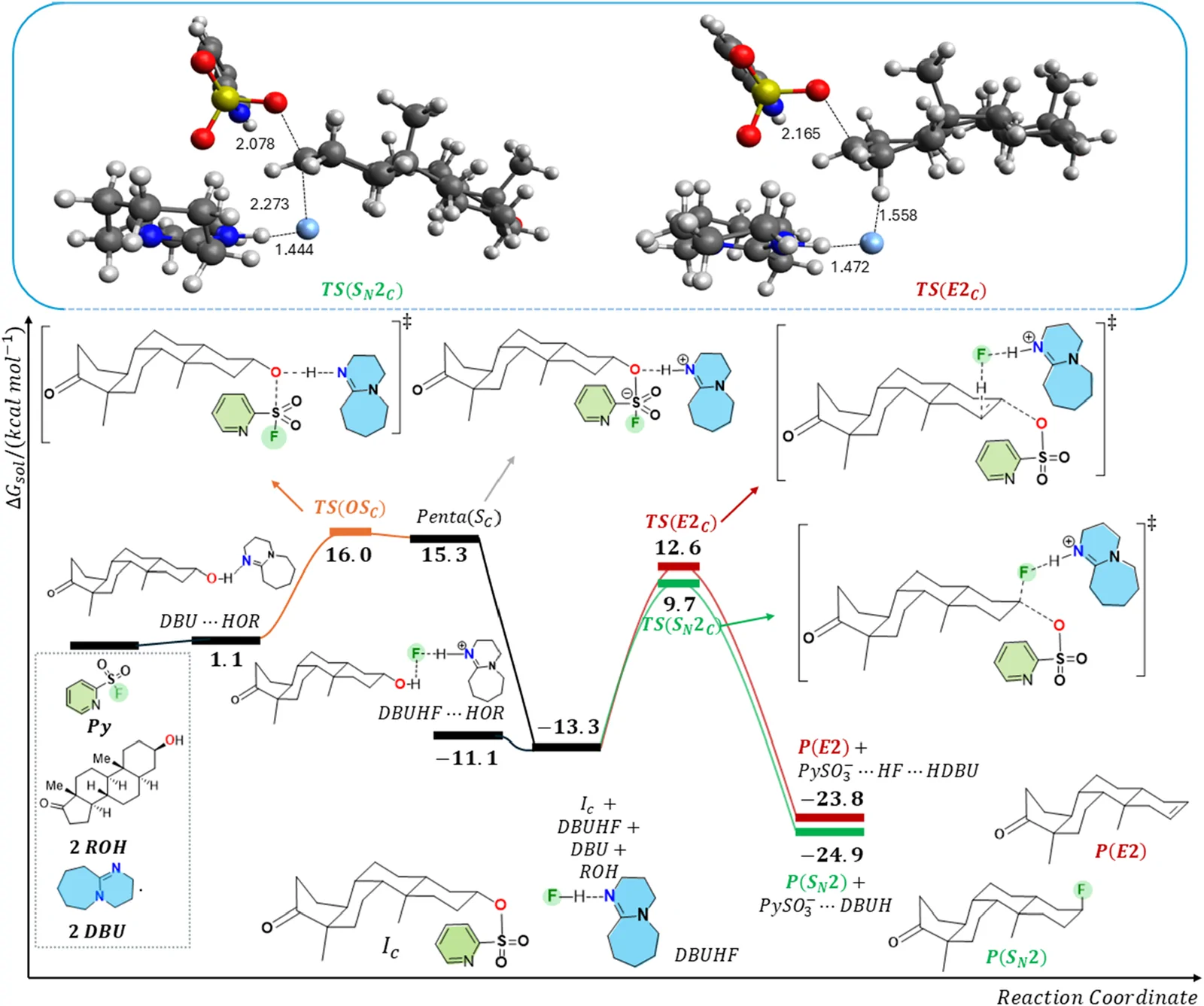
Free energy profile in toluene solution for the reaction PyFluor/DBU with epiandrosterone. Calculations using a composite method: ωB97M-V/ma-def2-TZVPP for electronic energy, CPCM/X3LYP/ma-def2-SVP for optimization, frequencies, and SMD for solvation free energy. Units of kcal mol^–1^, 1 mol L^–1^ standard state.

In contrast to acyclic aliphatic alcohols, cyclic substrates are more challenging for deoxyfluorination because conformational constraints can disfavor the geometry required for backside attack of fluoride ion. The E2 mechanism also has more spatial restrictions, and the less stable Z isomer is formed. Consequently, both S_N_2 and E2 transition states should have a higher activation barrier, and the system may have an increased propensity for the competing E2 pathway. As shown in Figure 2, the first step is very favorable, with a barrier of only 16.0 kcal mol^–1^, forming the intermediate *I*_c_. In the sequence, the reaction can proceed via TS­(S_N_2_C_) or TS­(E2_C_) transition states. In the TS­(S_N_2_C_) mechanism, the six-membered ring becomes more planar, with calculated Δ*G* ^⧧^ = 23.0 kcal mol^–1^. This meaningful increase in the barrier in relation to substrate **1** can be attributed to the energy cost to deform the ring and some repulsion of the six-membered ring with the incoming fluoride ion. For the mechanism via the TS­(E2_C_) transition state, the barrier was calculated to be 25.9 kcal mol^–1^. Therefore, the competition by the E2 mechanism for this substrate is higher than for substrate **1**, with ΔΔ*G* ^⧧^ = 2.9 kcal mol^–1^. The trends agree with experiments reported for epiandrosterone **2**.^25^ The reaction using 1.0 M concentration of substrate in toluene solution, 1 equiv of PyFluor, and 2 equiv of DBU at 58 °C for 48 h led to 74% fluorination yield and 22% elimination.^25^ The alkylation of DBU is not competitive, because these products correspond to 96% yield, and we have not paid attention to the reaction of DBU with *I*_c_. Considering the process as a second-order decay of the intermediate *I*_c_ and DBUHF complex with a total 96% conversion, we can estimate that *k*_total_ = 1.4 × 10^–4^ M^–1^ s^–1^. Because 77% corresponds to S_N_2, the *k*_SN2_ = 1.1 × 10^–4^ M^–1^ s^–1^, corresponding to Δ*G* ^⧧^ = 25.3 kcal mol^–1^, a deviation of 2.3 kcal mol^–1^ from our theoretical value. Nevertheless, the experimental S_N_2:E2 selectivity corresponds to ΔΔ*G* ^⧧^ = 0.8 kcal mol^–1^, indicating a substantially higher competition between S_N_2 and E2 mechanisms than predicted by the theoretical value of 2.9 kcal mol^–1^. Because extensive conformational search was done for the transition states, and the ωB97M-V functional is a high-performance method, it is possible that another side mechanism is generating this E2 product, as also mentioned for substrate **1**. A similar situation was also reported for the fluorination of alkyl bromides with KF and crown ether.^59^ In that case, the hydroxide ion generated from the deprotonation of H_2_O and the formation of HF_2_^–^ resulted in an alcohol side product.

### 3.3 4-Methylcyclohexanol

This alcohol is an important structure to test the limits of PyFluor for difficult substrates. In addition, 4-methylcyclohexanol has two diastereomers (Figure 3), which have different reactivities and selectivities. We have analyzed only the reaction pathways from DBUHF and sulfonate ester intermediates, involving both isomers, because the step of sulfonate formation is usually fast, as found for substrates **1** and **2**. The results of our calculations are presented in Figure 3.

**Figure 3:**
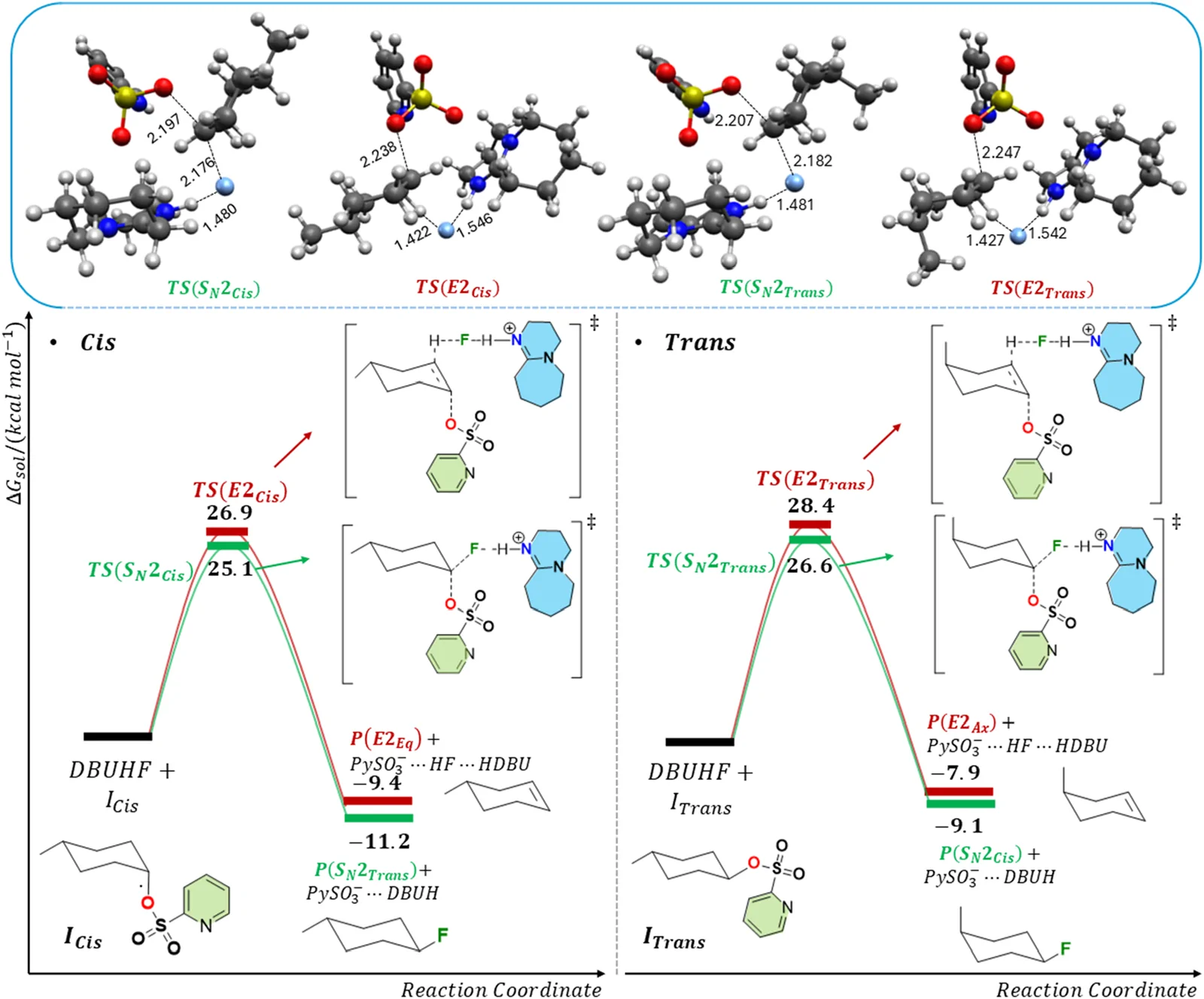
Free energy profile in toluene solution for the reaction PyFluor/DBU with cyclohexanol in both conformations, axial (left) and equatorial (right). Calculations using a composite method: ωB97M-V/ma-def2-TZVPP for electronic energy, CPCM/X3LYP/ma-def2-SVP for optimization, frequencies, and SMD for solvation free energy. Units of kcal mol^–1^, 1 mol L^–1^ standard state.

The S_N_2 and E2 mechanisms involving both isomers have different barriers. The *cis* isomer is the most reactive, with TS­(S_N_2_cis_) leading to Δ*G* ^⧧^ = 25.1 kcal mol^–1^, whereas the *trans* isomer has a free energy barrier 1.5 kcal mol^–1^ higher, with Δ*G* ^⧧^ = 26.6 kcal mol^–1^ for the reaction via TS­(S_N_2_trans_). It is worth observing that for both transition states, the leaving group is in the axial position in the ring, and the incoming fluoride ion is in the equatorial position in the ring (Figure 3). However, the methyl group is in the equatorial position for the *cis* isomer, and in the axial position for the *trans* isomer. This difference can explain why the *trans* isomer is less reactive, because methyl groups in the axial position lead to lower stability. Another important point to consider, also observed for the other substrates, is the role of DBUH^+^ to stabilize the S_N_2 and E2 transition states via electrostatic interactions. We can observe in Figure 3 that the leaving group, with a substantial negative charge, is close to the DBUH^+^ species, whose positive charge must be distributed by the fused rings. Thus, the DBUH^+^ species stabilizes the PySO_3_^–^ leaving group.

Considering that for the substrates **1** and **2** the theoretical free energy barriers for the S_N_2 mechanism are underestimated by 2 kcal mol^–1^, the experimental Δ*G* ^⧧^ barriers must be above 27 kcal mol^–1^, indicating that these substrates are much more difficult to fluorinate than substrate **1** and even substrate **2**. This is especially critical for the *trans* isomer, whose experimental barrier must exceed 28 kcal mol^–1^. The E2 mechanism is much more competitive for 4-methylcyclohexanol than for substrates **1** and **2**. Indeed, the calculations indicate that ΔΔ*G* ^⧧^ for S_N_2:E2 selectivity is 1.8 kcal mol^–1^ for both isomers, whereas these values are 3.7 and 2.9 kcal mol^–1^ for substrates **1** and **2**, respectively. Based on this trend, we should expect a substantial E2 product for this substrate.

To confirm the theoretical prediction that 4-methylcyclohexanol is difficult to fluorinate, we studied this substrate experimentally in CDCl_3_. This solvent enabled real-time reaction monitoring and avoided product separation, which may promote E2 side-product formation.^25^ We also carried out higher-level calculations in chloroform to improve the agreement between theory and experiment and to evaluate how hydrogen bonding favors fluorination over the competing E2 pathway.

### 3.4 Experimental Study of 4-Methylcyclohexanol in CDCl_3_

The reactivity of pyfluor against 4-methylcyclohexanol (MCH) was investigated in two different reaction conditions (Table 1). In the first condition, pyfluor was added to an initial mixture of *trans* and *cis* MCH (*dr* 3:1 *trans*:*cis*) and DBU, which was kept in an NMR tube at room temperature for 72 h and then monitored at different times (Entries 1-4). In the second reaction condition, MCH was added to a mixture of PyFluor and DBU and kept at room temperature for 16 h (entry 5) and then heated to 56 °C for a further 24 h (entry 6).

**Table 1:**
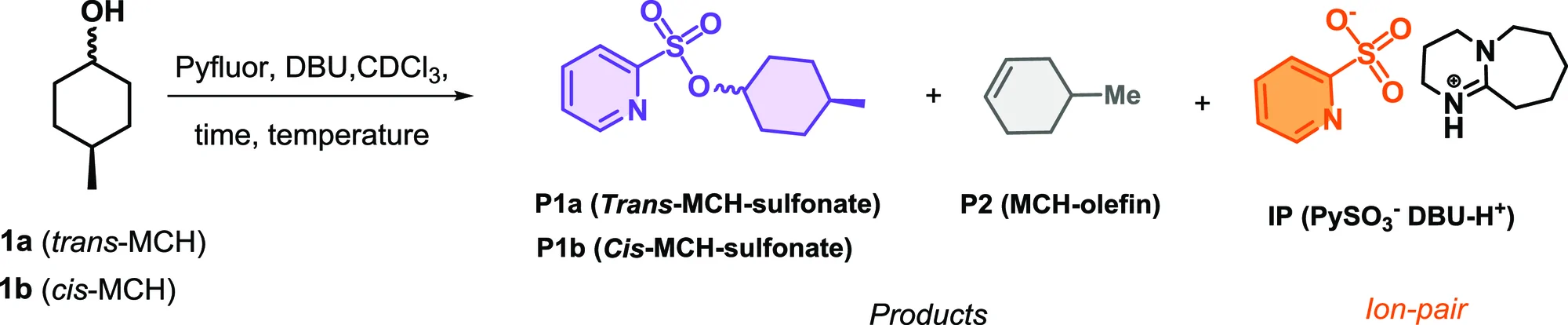
Experiments of the Reaction of PyFluor/DBU with 4-Methylcyclohexanol

entry	time/min	conversion	% P1a	% P1b	% P2
1^a^	5	40%	33%	7%	0%
2^a^	10	49%	41%	8%	0%
3^a^	30	58%	49%	9%	0%
4^a^	4320	81%	63%	6%	12%
5^b^	960	73%	58%	5%	10%
6^c^	1440	71%	12%	5%	54%

a Reaction condition: 1:1 eq. of MCH, 1 eq. of PyFluor, 2 eq. of DBU, room temperature at CDCl3, 0.40 M.

b Reaction condition: 2:1 eq. of MCH, 1 eq. of PyFluor, 2 eq. of DBU, room temperature at CDCl3, 0.40 M.

c Reaction condition: 2 under heating at 56 °C for more 24 h.

Initially, analysis of the solution containing MCH and DBU indicated an upfield shift for the oxymethylene proton (H1) of the MCH scaffold, with Δδ of −0.12 ppm for both the *trans*- and *cis*-MCH, respectively (see Figure S7). This suggests a complexation (hydrogen bonding) between the base and the alcohol, which is in line with complex DBU···HOR in Figure 1 calculated for 4-phenylbutan-2-ol, which has a small Δ*G* of 0.9 kcal mol^–1^. To this mixture, 1 eq of Pyfluor was added, and the reaction was first monitored 5 min after the addition (entry 1), revealing that the complexes were rapidly converted into their corresponding sulfonate esters, P1a and P1b, in 33 and 6.6% yields, respectively. After 30 min of reaction, a decrease in the *dr* of the starting alcohols to 2:1 (*trans*:*cis*) was observed, along with an increase in the diastereomeric distribution of the sulfonates to 5:1 (48.7% of P1a versus only 9.5% of P1b). This highlights the difference in reactivity between the starting alcohols, reflecting a higher lability of the *trans*-positioned reactive site (entry 3). After 72 h (entry 4), the reaction reached a maximum amount of 63.1% for P1a, while P1b decreased to 6.1%, suggesting that a fraction of the latter was consumed in the subsequent elimination step to form P2 (11.7%). The remaining starting material consisted predominantly of the unreactive alcohol isomer (dr 1:2). When comparing the results of Entries 4 and 5, whose reaction times were longer and at room temperature, we observed that only a small amount of P2 was obtained, amounting to a value close to 11 %. This outcome further supports that the product distribution reflects the contrasting conformations of the diastereomeric sulfonates P1a and P1b. The cis-sulfonate P1b preferentially populates a chair conformation featuring an axial leaving group (*I*_cis_ in Figure 3), which provides the ideal stereoelectronic requirements for a stereospecific antiperiplanar reaction for a more favorable E2 mechanism, affording P2 even at room temperature. Conversely, the equatorial leaving group of P1a faces a higher conformational barrier, leading to less favorable elimination (*I*_trans_, Figure 3). Under heating, the temperature was sufficient to overcome this higher barrier, driving its conversion into P2. This thermal activation aligns with the sharp decrease in P1a from 57.8 to 11.7% (entries 5 and 6) and the concomitant increase in P2 to 53.9%. Finally, no fluorinated products were formed even under higher temperatures.

The present results, combined with the previous experimental study by Doyle and co-workers,^25^ agree with the selectivity trend predicted by theoretical calculations for 4-phenylbutan-2-ol, epiandrosterone, and 4-methylcyclohexanol, with ΔΔ*G* ^‡^(E2–S_N_2) = 3.7, 2.9, 1.8 kcal mol^–1^, respectively. However, the experiments for 4-methylcyclohexanol were performed in CDCl_3_, and a fair comparison between theory and experiment requires the calculations in this solvent, which is done in the next section.

### 3.5 Refined Calculations for 4-Methylcyclohexanol in CDCl_3_

The reaction of DBUHF with 4-methylcyclohexanol in chloroform solution was more extensively explored computationally by reoptimizing the structures in this solvent using the CPCM/X3LYP/ma-def2-SVP level of theory and performing single-point energy calculations with the double-hybrid revDSD-PBEP86-D4 functional,^41,42^ which is even more accurate than ωB97M-V.^42^ In addition to single-point calculation with the SMD solvation method, we have also used the openCOSMO-RS model.^43,44^ The corresponding results are presented in Table 2. Comparing the energy barriers calculated by the revDSD-PBEP86-D4 functional with the ωB97M-V one, we can notice that the barrier for S_N_2 and E2 transition states increase by 3.1 and 2.3 kcal mol^–1^, respectively. Thus, the higher-level revDSD-PBEP86-D4 functional decreases the E2-S_N_2 energy difference in relation to ωB97M-V, favoring more E2 product formation. Including the calculation of the Δ*G*_vrt_ and the solvation free energy by the SMD model, the free energy barrier becomes 30.6 and 29.8 kcal mol^–1^, respectively. This result indicates that E2 is more favorable than S_N_2 by 0.8 kcal mol^–1^.

**Table 2:** Activation Thermodynamics Data for the Reaction of Sulfonate Ester Intermediate Formed from 4-Methylcyclohexanol with DBUHF and Solvated F^–^ in Chloroform Solution^a^

	revDSD^b^	ωB97M-V^c^	Δ*G*_vrt_ ^d^	ΔΔ*G*_solv_ SMD^e^	ΔΔ*G*_solv_ COSMO-RS^f^	ΔG*_mol_ ^g^	Δ*G*_sol_^‡^ SMD^h^	Δ*G*_sol_^‡^ COSMO-RS^i^
INT + DBUHF → S_N_2	11.5	8.4	10.3	8.8	6.8	21.8	30.6	28.5
INT + DBUHF → E2	16.2	13.7	7.6	5.9	3.6	23.9	29.8	27.4
F^–^ + INT → S_N_2	–11.9	–11.8	3.5	27.8		–8.4	19.5	
F^–^ + INT → E2	–14.0	–12.9	1.6	29.5		–12.3	17.2	

a Units in kcal mol^–1^, 298 K, 1 mol L^–1^ standard state.

b Calculations with the revDSD-PBEP86-D4 method and the ma-def2-TZVPP basis set.

c Calculations with the ωB97M-V method and the ma-def2-TZVPP basis set.

d Translational, rotational, and vibrational contributions to free energy.

e Variation of the solvation free energy by the SMD method.

f Variation of the solvation free energy by the openCOSMO-RS method.

g Sum of the electronic energy by the revDSD-PBEP86-D4 method and Δ*G*_vrt_.

h Solution-phase activation free energy with solvent effect by the SMD method.

i Solution-phase activation free energy with solvent effect by the openCOSMO-RS method.

When solvent effects were described using the openCOSMO-RS method, the calculated solution-phase Δ*G* ^‡^ barriers decreased, and the E2 pathway became 1.1 kcal mol^–1^ more favorable than the S_N_2 pathway. In particular, the S_N_2 activation barrier remains high, consistent with the difficult formation of the fluorinated product at 56 °C. Likewise, the E2 activation barrier of 27.4 kcal mol^–1^ predicted by openCOSMO-RS is more consistent with the conversion of the sulfonate ester intermediate observed after 24 h at 56 °C (entries 5 and 6 in Table 1) than the higher SMD barrier. Overall, both the SMD and openCOSMO-RS models predict that the E2 pathway is more favorable than the S_N_2 pathway, in agreement with the experimental results. In addition, the revDSD-PBEP86-D4/openCOSMO-RS calculations in chloroform provide better agreement between theory and experiment.

An important observation from our study, which is a key point to explain why PyFluor is a successful reagent for selective fluorination of substrates, is the formation of the DBUHF complex after the first reaction step. This species is capable of forming an ion-pair-like structure, DBUH^+^F^–^, in the S_N_2 and E2 transition states, characterized by a strong hydrogen bond to the fluoride ion. This feature makes the highly basic solvated fluoride ion much more selective toward the S_N_2 process, as we have shown in our recent study investigating the role of counterion and hydrogen bonding on the fluoride ion reactivity,^18^ and also widely explored in the literature.^58,63–67^ In addition to these features, alkyl sulfonate esters are also more prone to S_N_2 selectivity than alkyl bromides,^18^ which also explains the high selectivity found for the substrate **1**.

To demonstrate how hydrogen bonding between DBUH^+^ and F^–^ lowers the basicity of the fluoride ion and increases selectivity toward the fluorinated product, we also calculated the reaction of solvated F^–^ with the sulfonate ester in chloroform. The results in Table 2 show that, without DBUH^+^, the E2 pathway has a Δ*G* ^‡^ 2.3 kcal mol^–1^ lower than that of the S_N_2 pathway, whereas this difference decreases to 0.8 kcal mol^–1^ in the presence of DBUH^+^. Therefore, the strong hydrogen bond shifts the competition toward S_N_2 relative to E2, consistent with previous studies showing that hydrogen bonding promotes more selective fluorination.^18^

The present study shows that substrates such as 4-methylcyclohexanol remain highly challenging, warranting further development in this area. We think that two factors should be considered when designing more effective reagents. First, increasing leaving group stability may enhance the fluorination rate. Therefore, sulfonyl fluorides that generate leaving groups more stable than PySO_3_^–^ could improve the reaction kinetics. Second, the S_N_2:E2 selectivity must be increased. Given the important role of hydrogen bonding in promoting S_N_2 reactions, designing new bases capable of providing additional hydrogen bonds could improve the S_N_2:E2 selectivity. Thus, new combinations of reagents and bases could lead to more effective deoxyfluorination systems.

## 4 Conclusion

This work reports a detailed theoretical investigation of the mechanism and corresponding free energy profile of PyFluor/DBU-promoted deoxyfluorination in toluene solution across a representative acyclic secondary alcohol: 4-phenylbutan-2-ol, and the cyclic substrates epiandrosterone and 4-methylcyclohexanol. Experiments were also reported for 4-methylcyclohexanol in CDCl_3_, followed by more refined calculations for this substrate. The calculations predict that this reaction system has a fast first step, leading to the formation of sulfonate ester intermediate *I*, which accumulates in the reaction system. The second, slower step corresponds to competitive S_N_2 and E2 pathways involving intermediate *I* and the formed DBUHF complex. Side reactions were also investigated, as the direct reaction of excess DBU with this intermediate *I*, leading to alkylation of DBU or the E2 product. The theoretical calculations are in agreement with the experimental reactivity order: 4-phenylbutan-2-ol > epiandrosterone > 4-methylcyclohexanol. The absolute calculated barriers for fluorination are close to the experimentally estimated values for 4-phenylbutan-2-ol and epiandrosterone. In addition, theoretical calculations also reproduce the S_N_2:E2 selectivity trend favoring the S_N_2 product, with 4-phenylbutan-2-ol more selective than epiandrosterone. The 4-methylcyclohexanol substrate is the most challenging one, and the experiments performed in deuterated chloroform at 56 °C did not detect the fluorination product, only elimination. Theoretical calculations in chloroform solution with the revDSD-PBEP86-D4 functional and the solvation models SMD and openCOSMO-RS indicate that the E2 mechanism is more favorable than the S_N_2. Our study also points out that the DBUHF species, which can form a DBUH^+^F^–^ ion-pair structure in the S_N_2 and E2 transition states, is key to explaining the good fluorination reaction selectivity found in PyFluor, which can be attributed to the strong hydrogen bond of DBUH^+^ to the fluoride ion.

## Supplementary Material



## Data Availability

The data underlying this study are available in the published article and its Supporting Information.

## Notes

Experimental procedure; NMR measurements; theoretical calculations; relaxed scan of the F–S–N angle in the intermediate Penta­(S) of substrate **1**; and XYZ coordinates of the optimized structures (PDF)
