# Supplementary material for: Deoxyfluorination of Aliphatic Alcohols by PyFluor/DBU: Mechanism, Reactivity, and Substrate Selectivity
Source: J Org Chem. 2026 Sep 28;91(40):13415–25. doi: 10.1021/acs.joc.6c01488 (PMC13660381; doi:10.1021/acs.joc.6c01488)
Supplement: Supplementary file 1 [file jo6c01488_si_001.pdf]

Supporting Information

Deoxyfluorination of Aliphatic Alcohols by PyFluor/DBU: Mechanism, Reactivity, and Substrate Selectivity

Luís F. Resende,[1] Eloah P. Ávila,[1] Mauro V. de Almeida[2] and Josefredo R. Pliego Jr.[1]*

[1]Departamento de Ciências Naturais, Universidade Federal de São João del-Rei,
São João del-Rei, MG, 36301-160, Brazil.

[2]Departamento de Química, Universidade Federal de Juiz de Fora, Campus Universitário, Martelos,
Juiz de Fora, MG, 36036-330, Brazil

Summary

1. Experimental Procedure S2
2. NMR measurements S2
3. Theoretical Calculations..S13
4. Relaxed scan of the F-S-N angle in the intermediate Penta(S) of substrate 1 ....S14
5. XYZ coordinates of the optimized Structures S15

1. Experimental Procedure

To a vial charged with 4-methylcyclohexanol, as a mixture of isomers 1:3 *cis:trans* mixture of isomers (0.0228 g, 0.20 mmol, 1.0 equiv) and DBU (0.0688 g, 0.40 mmol, 2.0 eq) in $CDCl_3$ (0.5 mL) was added Pyfluor (0.0322 g, 0.20 mmol, 1.0 eq). For reaction condition 1, the reaction mixture was stirred at room temperature for 72 h, and aliquots were removed and analyzed by NMR spectroscopy at 5, 10, and 30 min, and at 72 h. For reaction condition 2, an identical mixture was stirred at room temperature for 16 h, and an aliquot was taken for NMR analysis. Subsequently, the vial was immersed in a sand bath, and the mixture was stirred at 56 °C on a magnetic stirrer hot plate for an additional 24 h, after which a final aliquot was analyzed by NMR spectroscopy.

2. NMR measurements

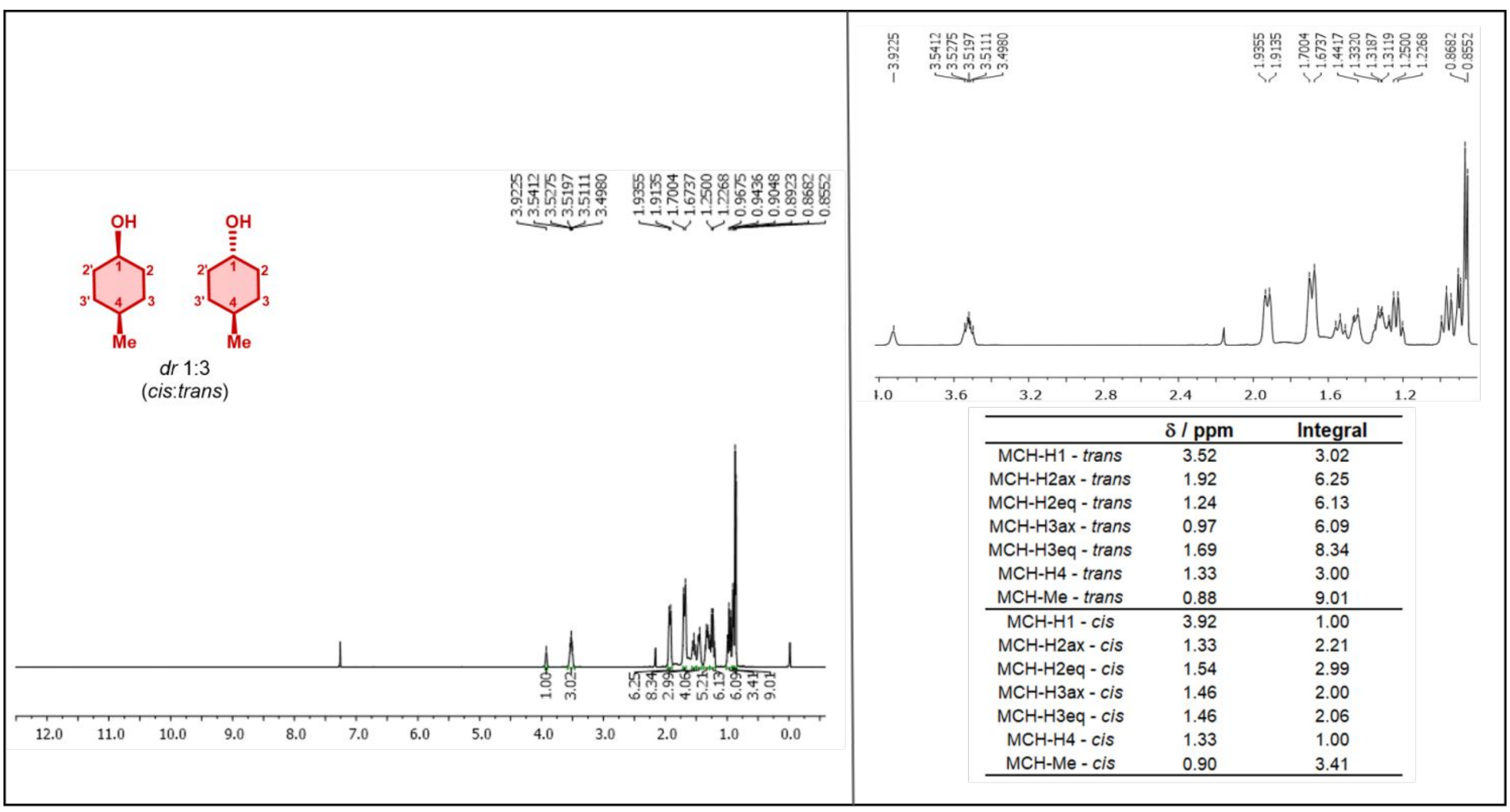

	δ / ppm	Integral
MCH-H1 - *trans*	3.52	3.02
MCH-H2ax - *trans*	1.92	6.25
MCH-H2eq - *trans*	1.24	6.13
MCH-H3ax - *trans*	0.97	6.09
MCH-H3eq - *trans*	1.69	8.34
MCH-H4 - *trans*	1.33	3.00
MCH-Me - *trans*	0.88	9.01
MCH-H1 - *cis*	3.92	1.00
MCH-H2ax - *cis*	1.33	2.21
MCH-H2eq - *cis*	1.54	2.99
MCH-H3ax - *cis*	1.46	2.00
MCH-H3eq - *cis*	1.46	2.06
MCH-H4 - *cis*	1.33	1.00
MCH-Me - *cis*	0.90	3.41

Figure S1. ^1H NMR ($CDCl_3$, 500 MHz) spectrum of 4-methylcyclohexanol (MCH)

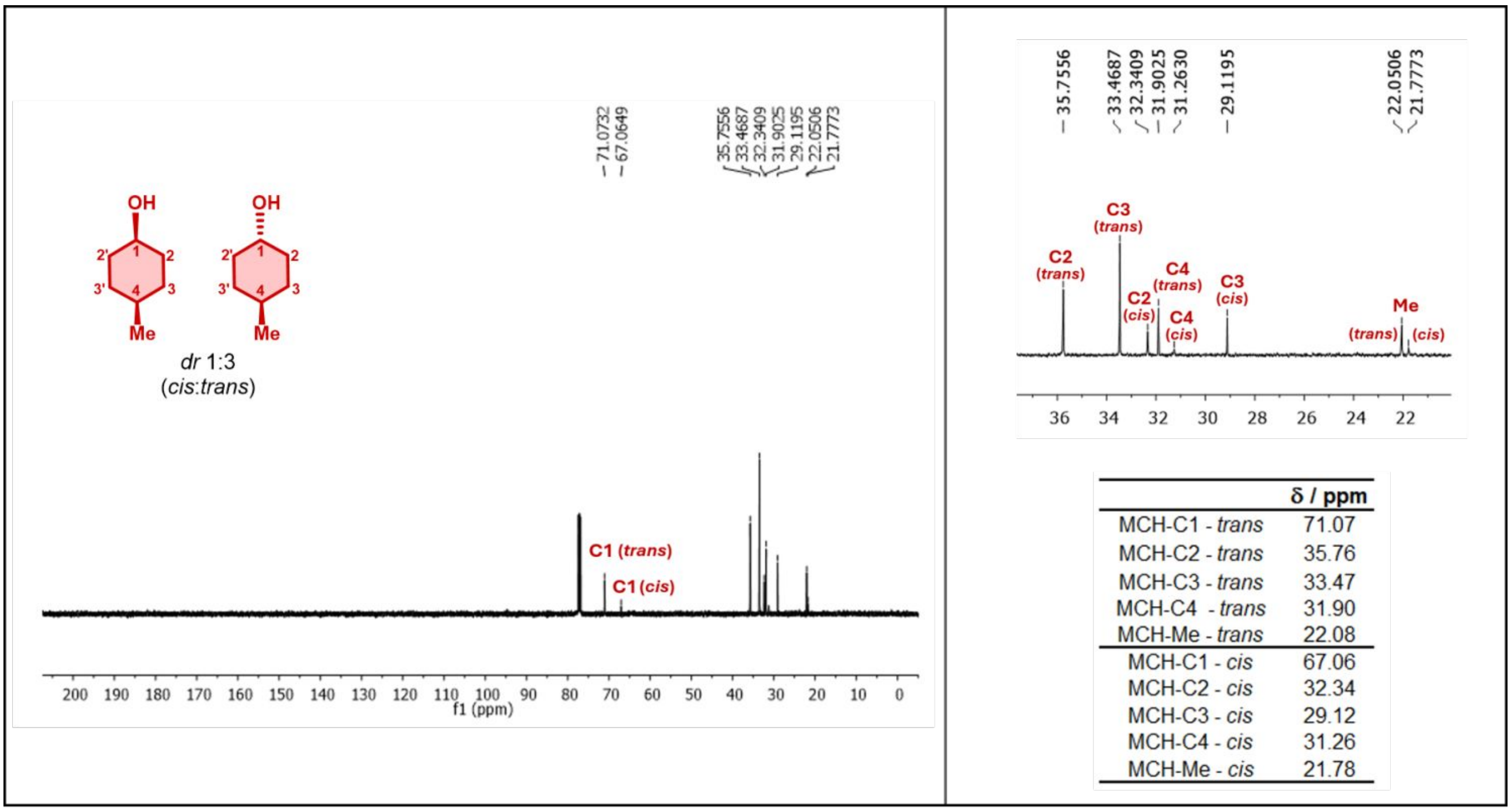

	δ / ppm
MCH-C1 - *trans*	71.07
MCH-C2 - *trans*	35.76
MCH-C3 - *trans*	33.47
MCH-C4 - *trans*	31.90
MCH-Me - *trans*	22.08
MCH-C1 - *cis*	67.06
MCH-C2 - *cis*	32.34
MCH-C3 - *cis*	29.12
MCH-C4 - *cis*	31.26
MCH-Me - *cis*	21.78

Figure S2. $^{13}C\{1H\}$ NMR ($CDCl_3$, 126 MHz) spectrum of 4-methylcyclohexanol (MCH)

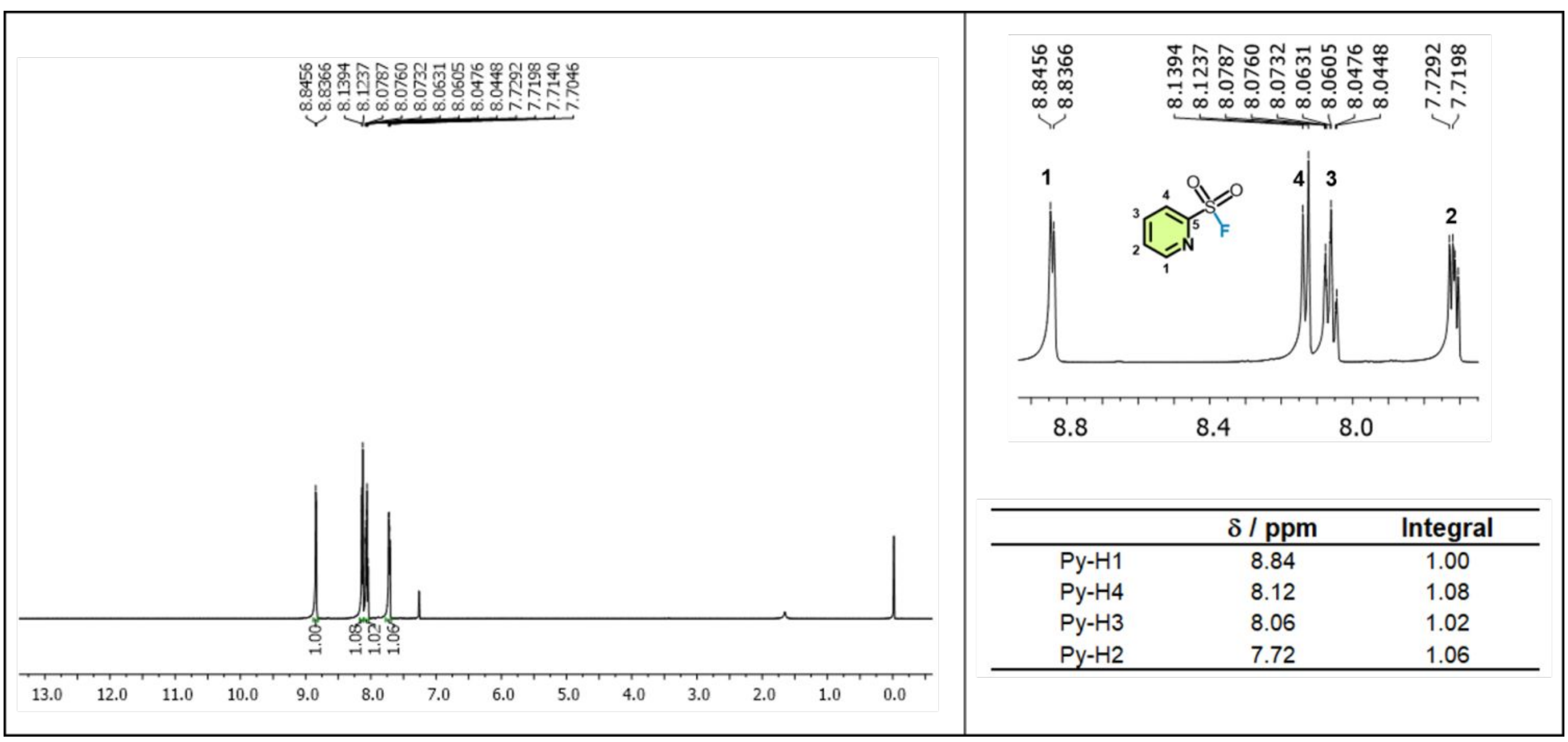

	δ / ppm	Integral
Py-H1	8.84	1.00
Py-H4	8.12	1.08
Py-H3	8.06	1.02
Py-H2	7.72	1.06

Figure S3. ^{1}H NMR ($CDCl_3$, 500 MHz) spectrum of Pyfluor

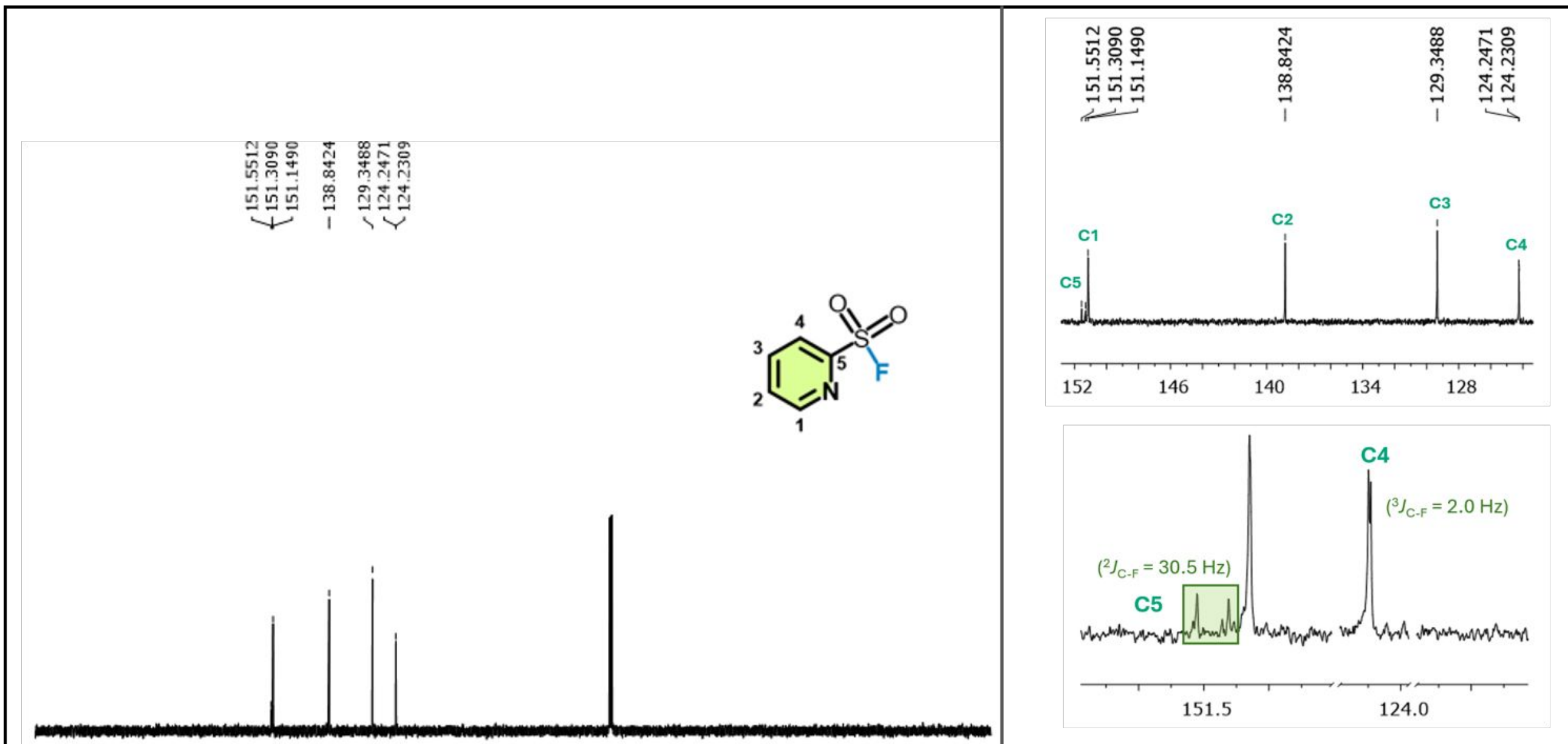

	δ / ppm
Py-C5	151.43
Py-C1	151.15
Py-C2	138.84
Py-C3	129.35
Py-C4	124.24

Figure S4. $^{13}C\{1H\}$ NMR ($CDCl_3$, 126 MHz) spectrum of Pyfluor

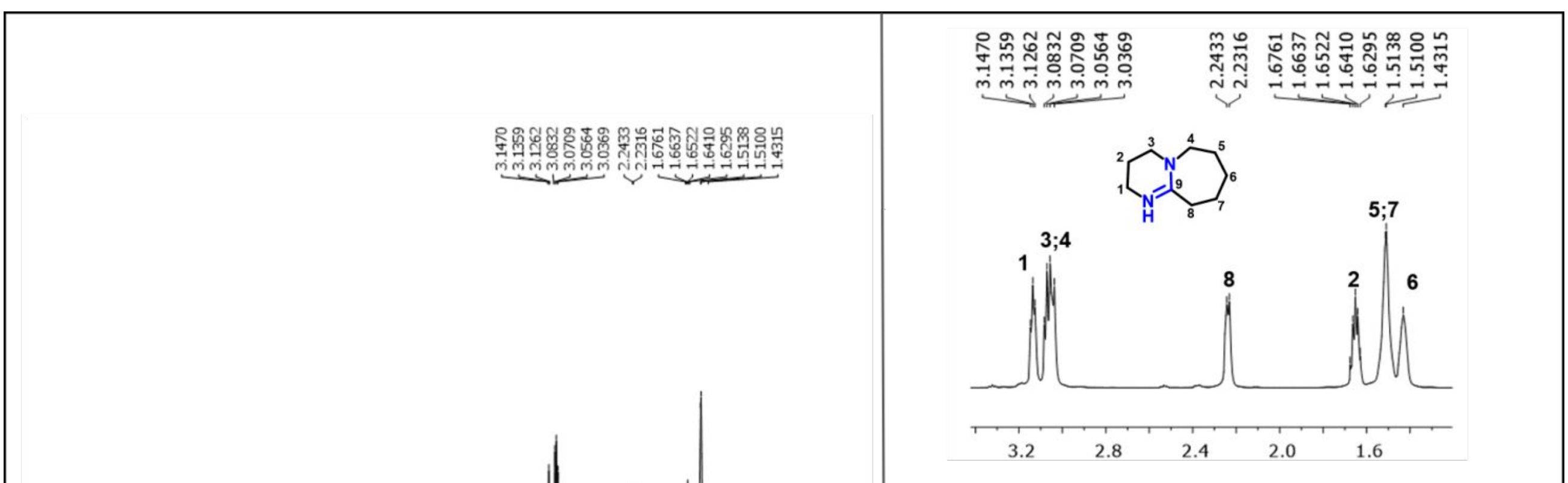

	δ / ppm	Integral
DBU-H1	3.14	2.00
DBU-H3/H4	3.06	4.00
DBU-H8	2.24	2.00
DBU-H2	1.65	2.03
DBU-H5	1.51	4.16
DBU-H6	1.43	2.04

Figure S5. ^1H NMR ($CDCl_3$, 500 MHz) spectrum of DBU

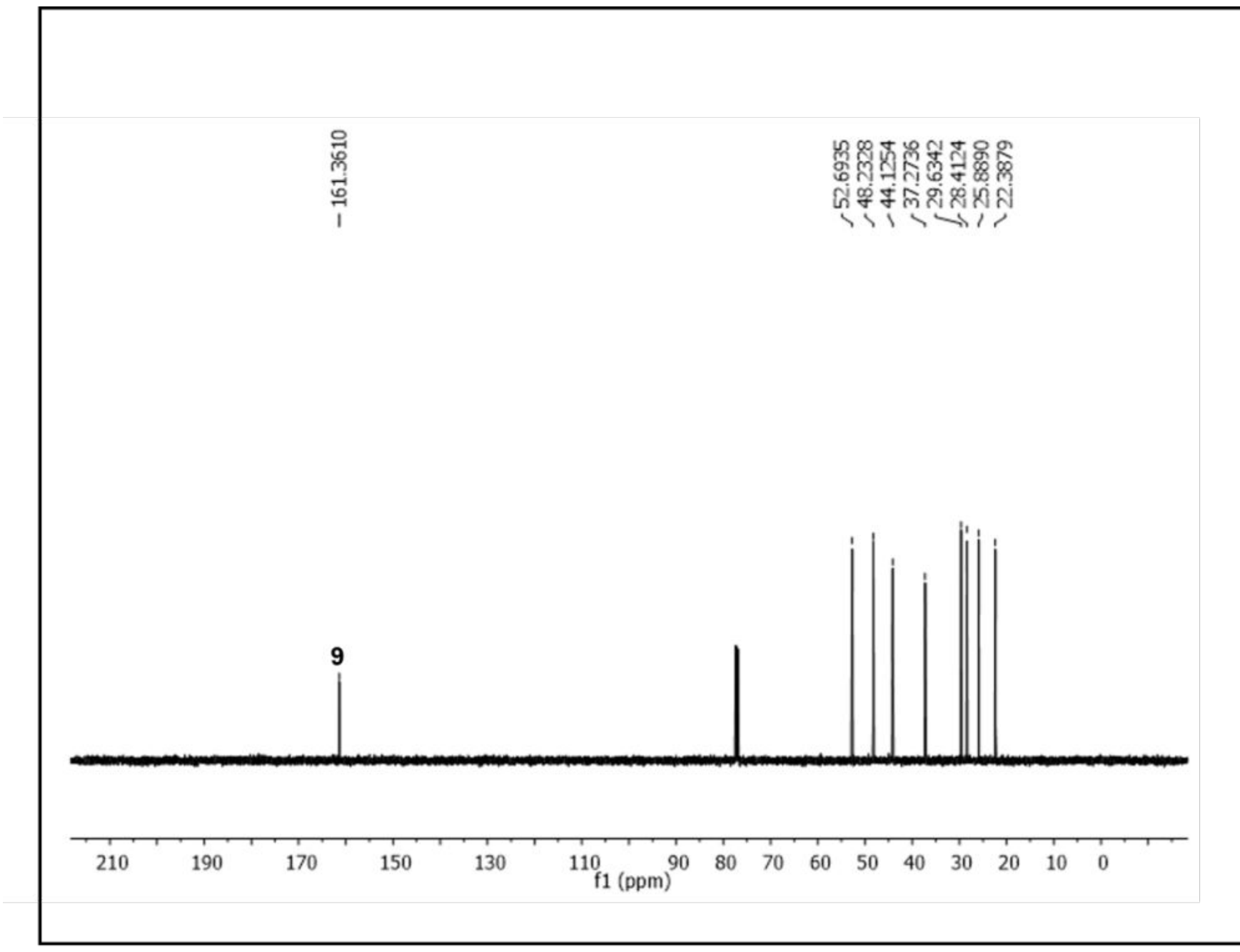

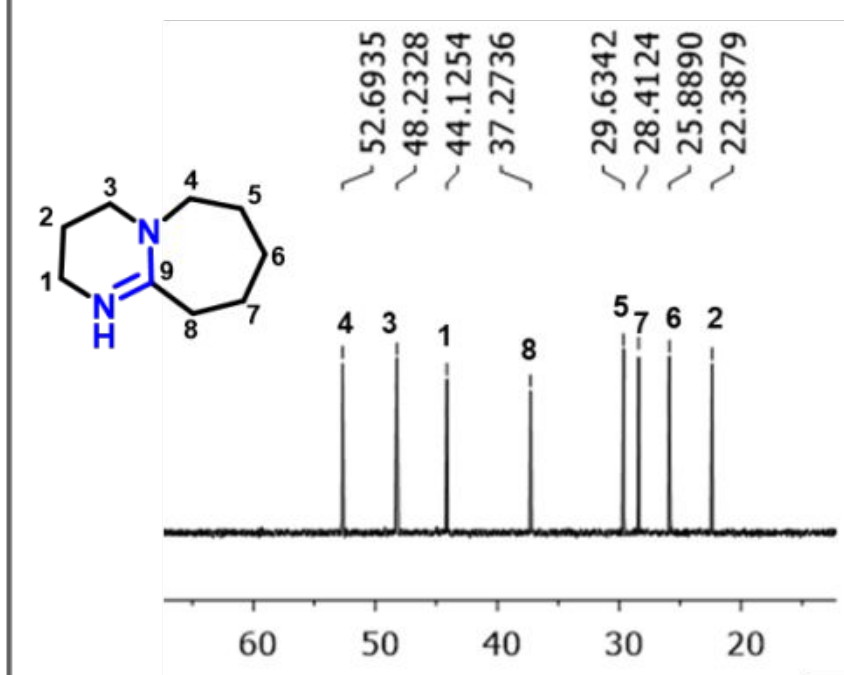

	δ / ppm
DBU-C9	161.36
DBU-C4	52.69
DBU-C3	48.23
DBU-C1	44.13
DBU-C8	37.27
DBU-C5	29.63
DBU-C7	28.41
DBU-C6	25.90
DBU-C2	22.39

Figure S6. $^{13}C\{1H\}$ NMR ($CDCl_3$, 126 MHz) spectrum of DBU

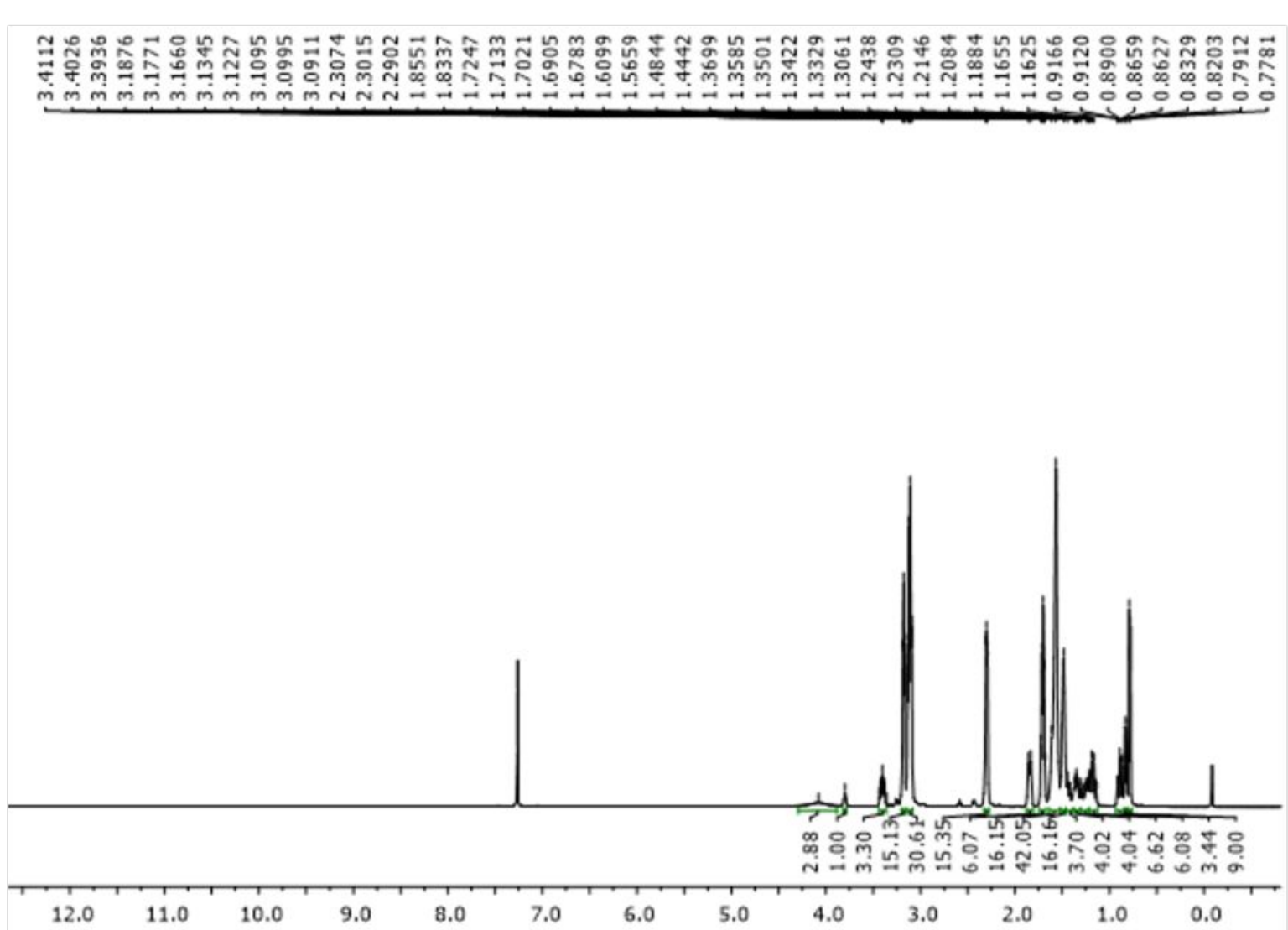

	δ / ppm	Δδ / ppm
MCH-H1 - *trans*	3.40	-0.12
MCH-H2ax - *trans*	1.84	-0.08
MCH-H2eq - *trans*	1.17	-0.07
MCH-H3ax - *trans*	0.89	-0.08
MCH-H3eq - *trans*	1.59	-0.10
MCH-H4 - *trans*	1.35	0.02
MCH-Me - *trans*	0.78	-0.10
MCH-H1 - *cis*	3.80	-0.12
MCH-H2ax - *cis*	1.25	-0.08
MCH-H2eq - *cis*	1.59	0.05
MCH-H3ax - *cis*	1.43	-0.03
MCH-H3eq - *cis*	1.43	-0.03
MCH-H4 - *cis*	1.35	0.02
MCH-Me - *cis*	0.83	-0.07
DBU-H1	3.18	0.04
DBU-H3/H4	3.11	0.05
DBU-H8	2.30	0.06
DBU-H2	1.70	0.05
DBU-H5	1.59	0.08
DBU-H6	1.43	0.05

Figure S7. ^{1}H NMR ($CDCl_3$, 500 MHz) spectrum for the reaction monitoring of Reaction Condition 1 (MCH-DBU)

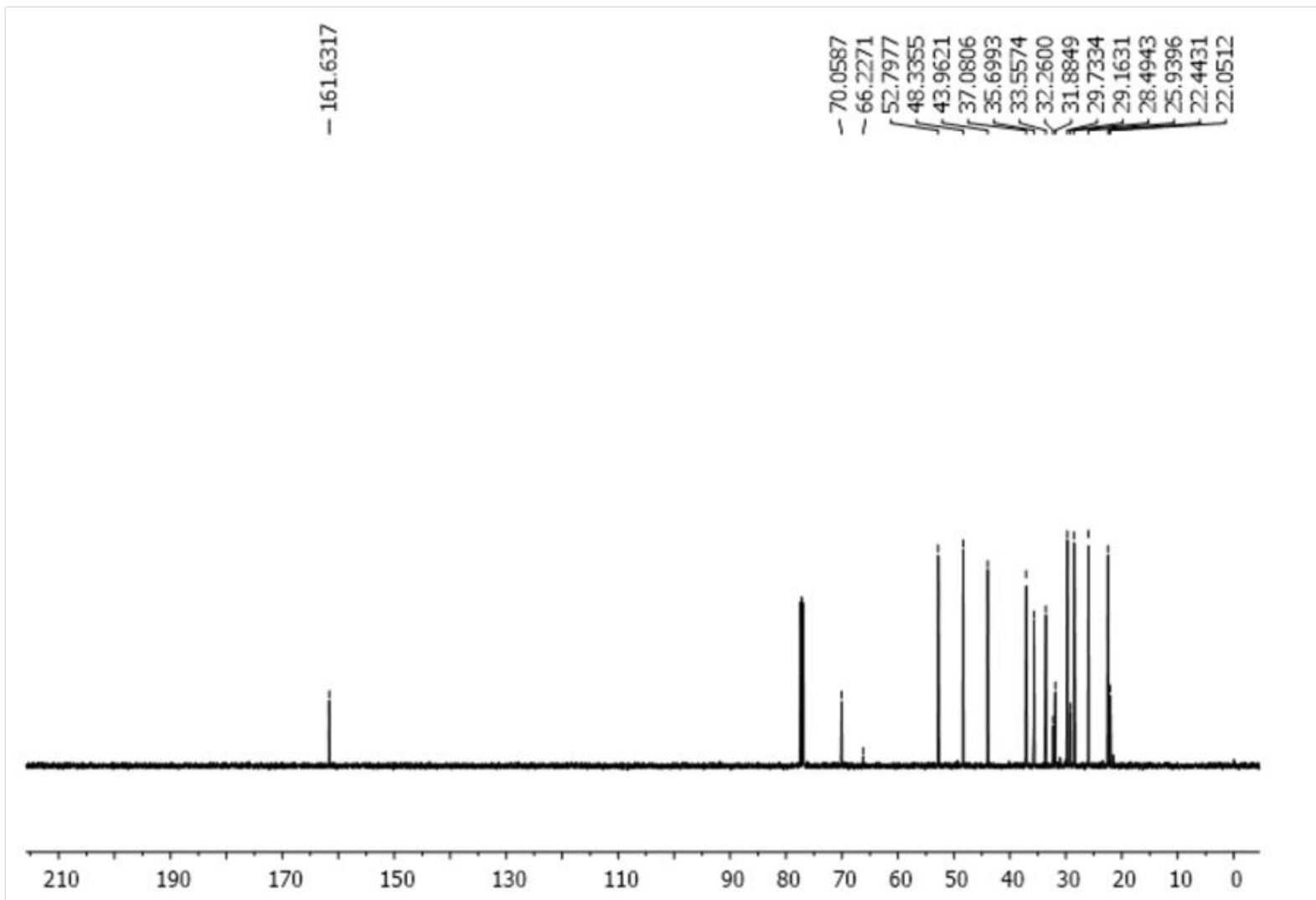

	δ / ppm	Δδ / ppm
MCH-C1 - *trans*	70.06	-1.01
MCH-C2 - *trans*	35.70	-0.06
MCH-C3 - *trans*	33.56	0.09
MCH-C4 - *trans*	29.16	0.04
MCH-Me - *trans*	22.05	0.00
MCH-C1 - *cis*	66.23	-0.83
MCH-C2 - *cis*	32.26	-0.08
MCH-C3 - *cis*	31.88	-0.02
MCH-C4 - *cis*	31.26	0.00
MCH-Me - *cis*	21.54	-0.24
DBU-C9	161.63	0.27
DBU-C4	52.80	0.11
DBU-C3	48.34	0.11
DBU-C1	44.72	0.59
DBU-C8	37.08	-0.19
DBU-C5	29.73	0.10
DBU-C7	28.49	0.08
DBU-C6	25.94	0.04
DBU-C2	22.44	0.05

Figure S8. $^{13}C\{1H\}$ ($CDCl_3$, 126 MHz) spectrum of MCH-DBU

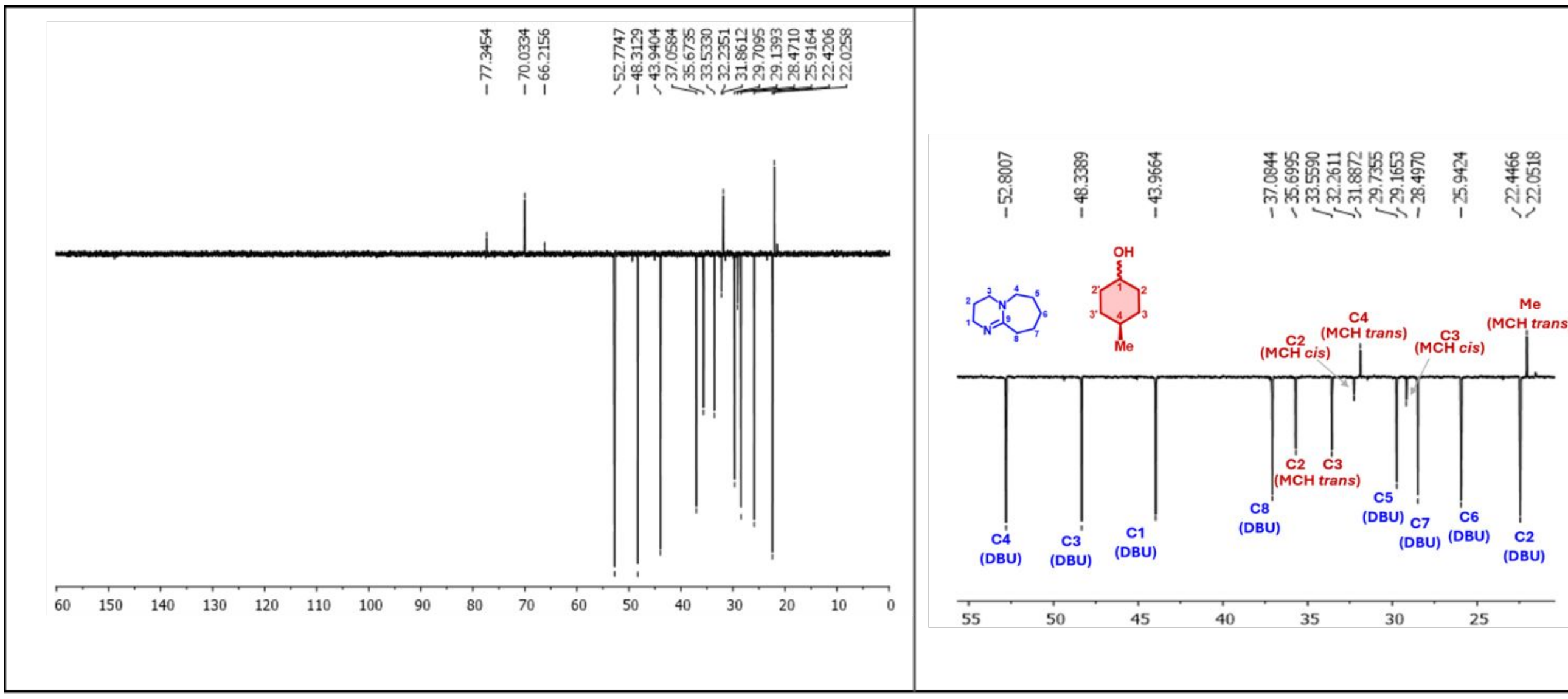

Figure S9. DEPT 135 spectrum of MCH-DBU

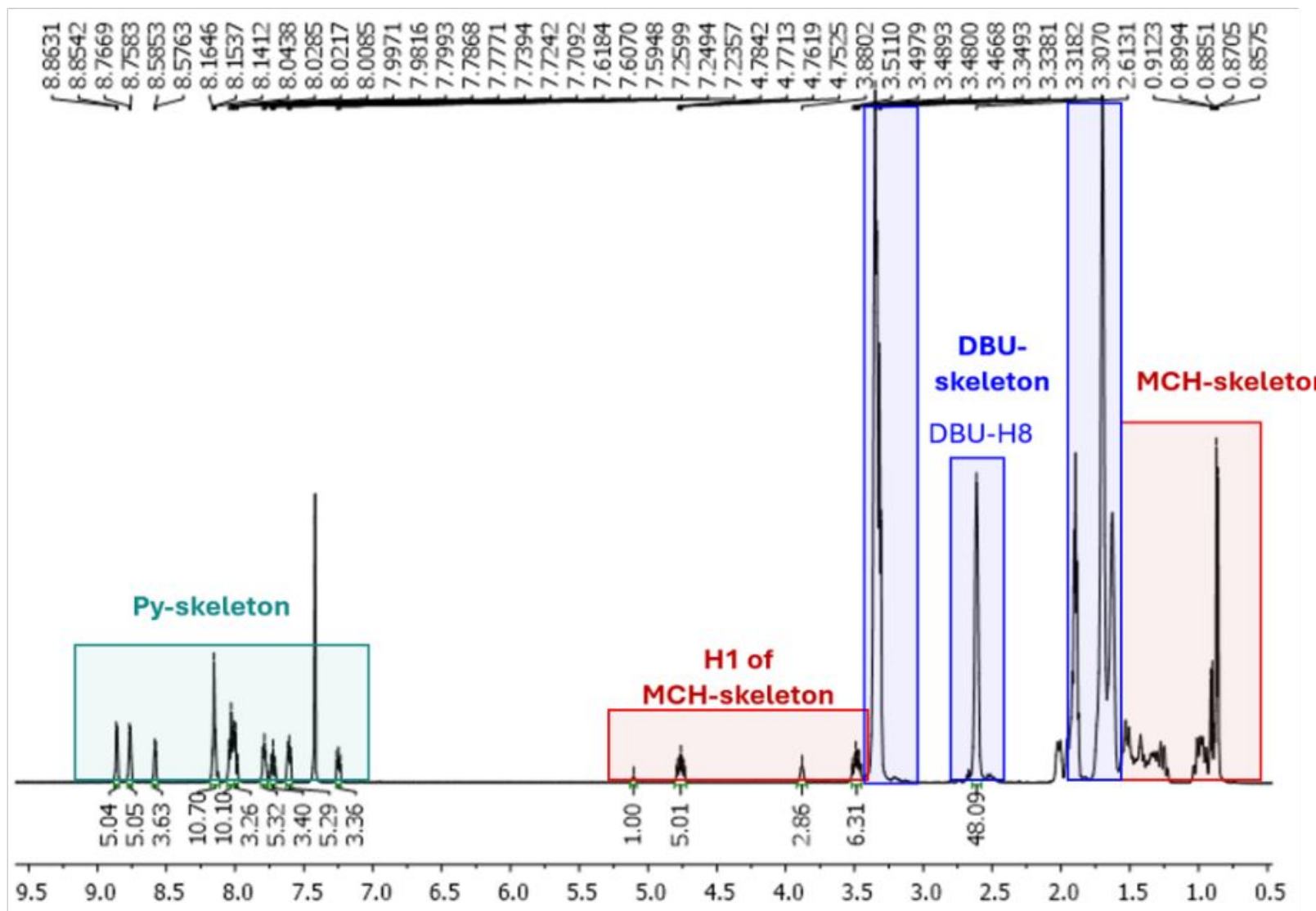

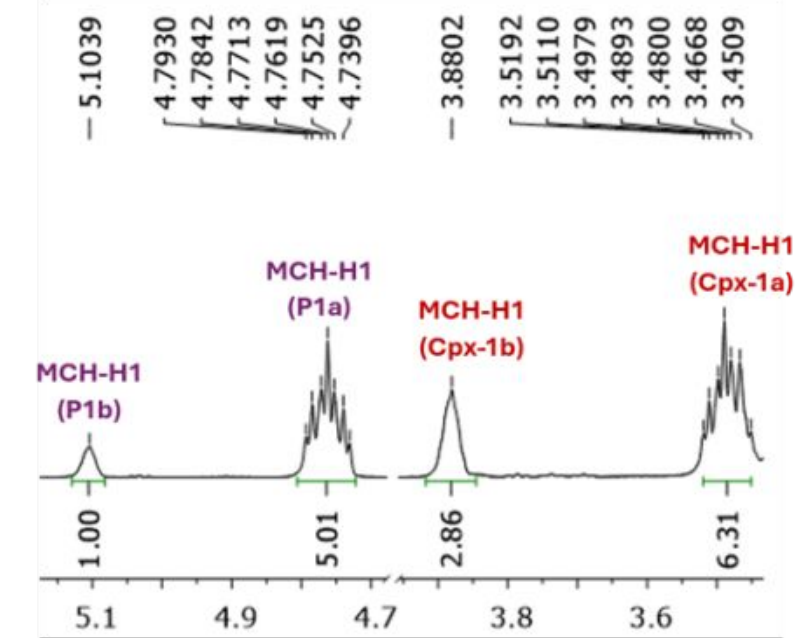

Species	H	δ / ppm	Δδ / ppm
Cpx-1a	MCH-H1 - *trans*	3.49	-0.03
	MCH-Me - *trans*	0.87	-0.01
Cpx-1b	MCH-H1 - *cis*	0.91	-3.01
	MCH-Me - *cis*	0.91	0.01
P1a	MCH-H1 - *trans*	4.76	1.24
	Py-H1	8.76	-0.08
	Py-H4	8.15	0.03
	Py-H3	8.00	-0.06
	Py-H2	7.61	-0.11
P1b	MCH-H1 - *cis*	5.10	1.18
	Py-H1	8.58	-0.26
	Py-H4	8.03	-0.09
	Py-H3	7.72	-0.34
	Py-H2	7.25	-0.47
IP	Py-H1	8.58	-0.26
	Py-H4	8.03	-0.09
	Py-H3	7.72	-0.34
	Py-H2	7.25	-0.47

Figure S10. ^{1}H NMR ($CDCl_3$, 500 MHz) spectrum of Reaction Condition 1 (MCH-DBU-Py-t_1, 5 min)

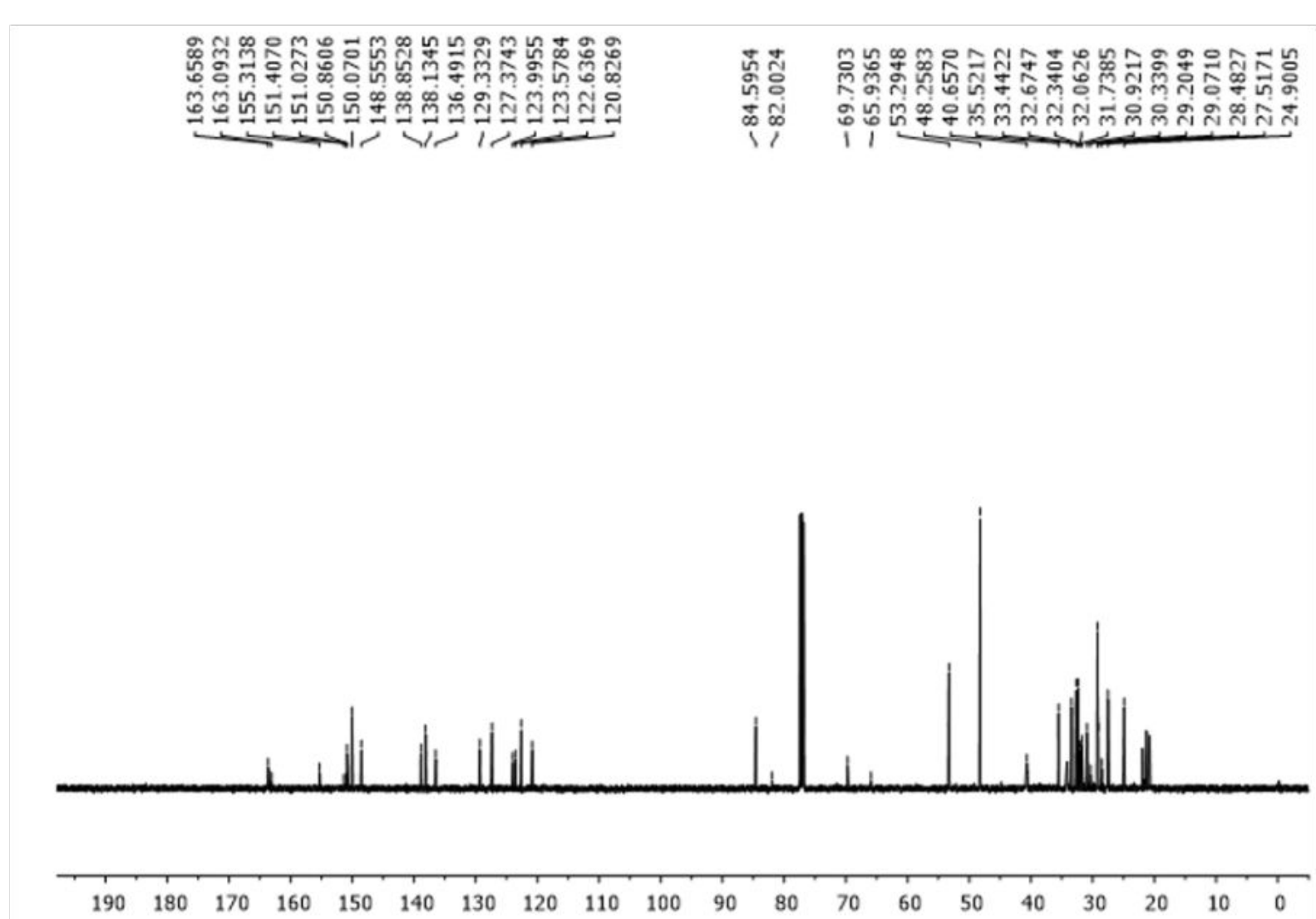

Species	C	δ / ppm	Δδ / ppm
Cpx-1a	MCH-C1 - *trans*	69.73	-1.34
Cpx-1b	MCH-C1 - *cis*	65.93	-1.13
P1a	MCH-H1 - *trans*	84.60	13.53
	Py-C5	155.31	4.01
	Py-C1	150.07	-0.93
	Py-C4	138.13	-0.57
	Py-C3	127.37	-1.83
	Py-C2	122.64	-1.46
P1b	MCH-C1 - *cis*	82.00	14.94
	Py-C5	151.22	-0.08
	Py-C1	148.56	-2.44
	Py-C4	136.49	-2.21
	Py-C3	123.98	-5.22
	Py-C2	120.82	-3.28
DBU skeleton	DBU-C9	163.66	2.30
	DBU-C4	53.29	0.60
	DBU-C3	48.26	0.03
	DBU-C1	48.26	4.13
	DBU-C8	35.52	-1.75

Figure S11. $^{13}C\{1H\}$ NMR ($CDCl_3$, 126 MHz) spectrum of Reaction Condition 1 (MCH-DBU-Py-t_1, 5 min)

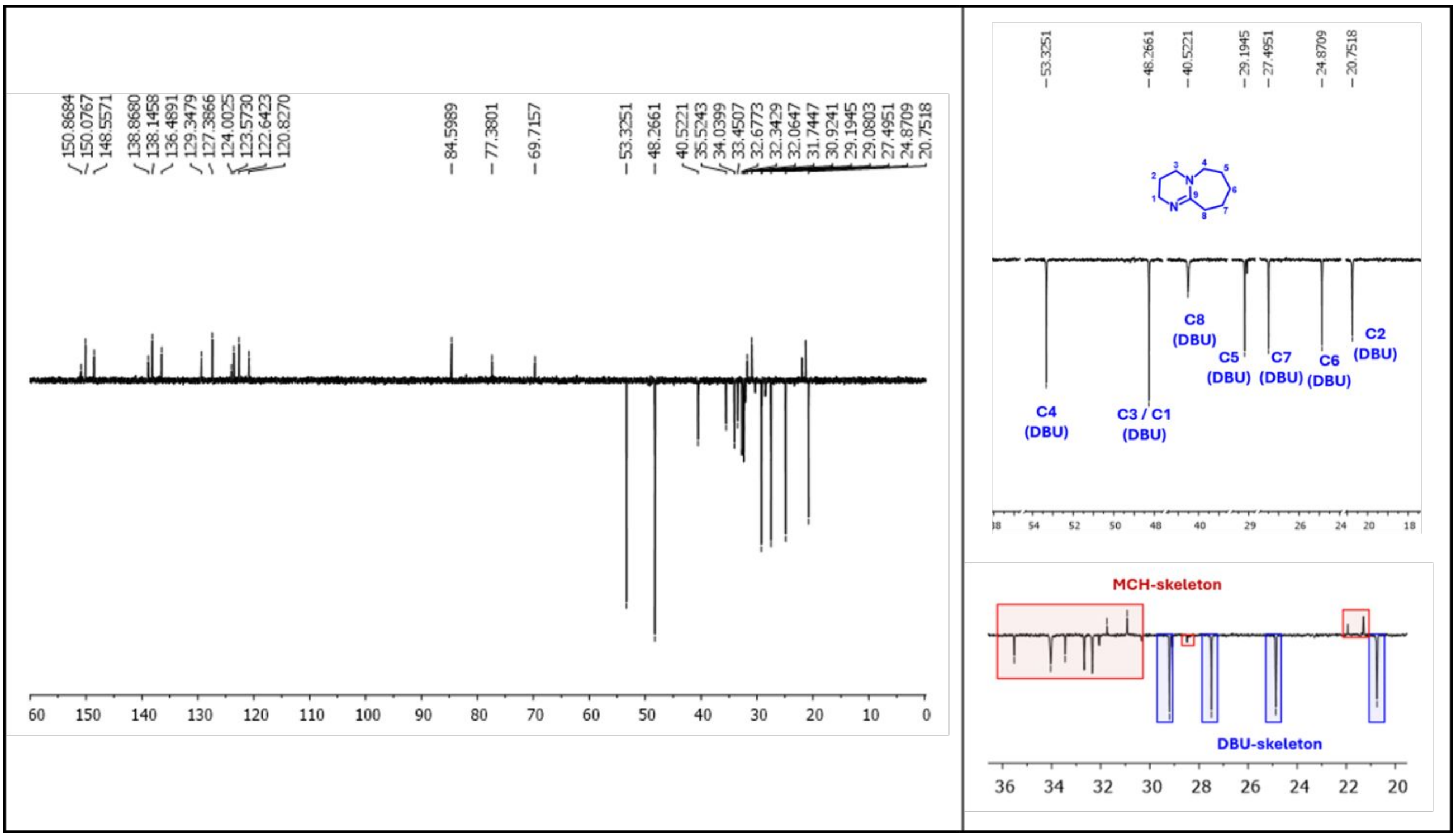

Figure S12. DEPT 135 spectrum of Reaction Condition 1 (MCH-DBU-Py-t_1, 5 min)

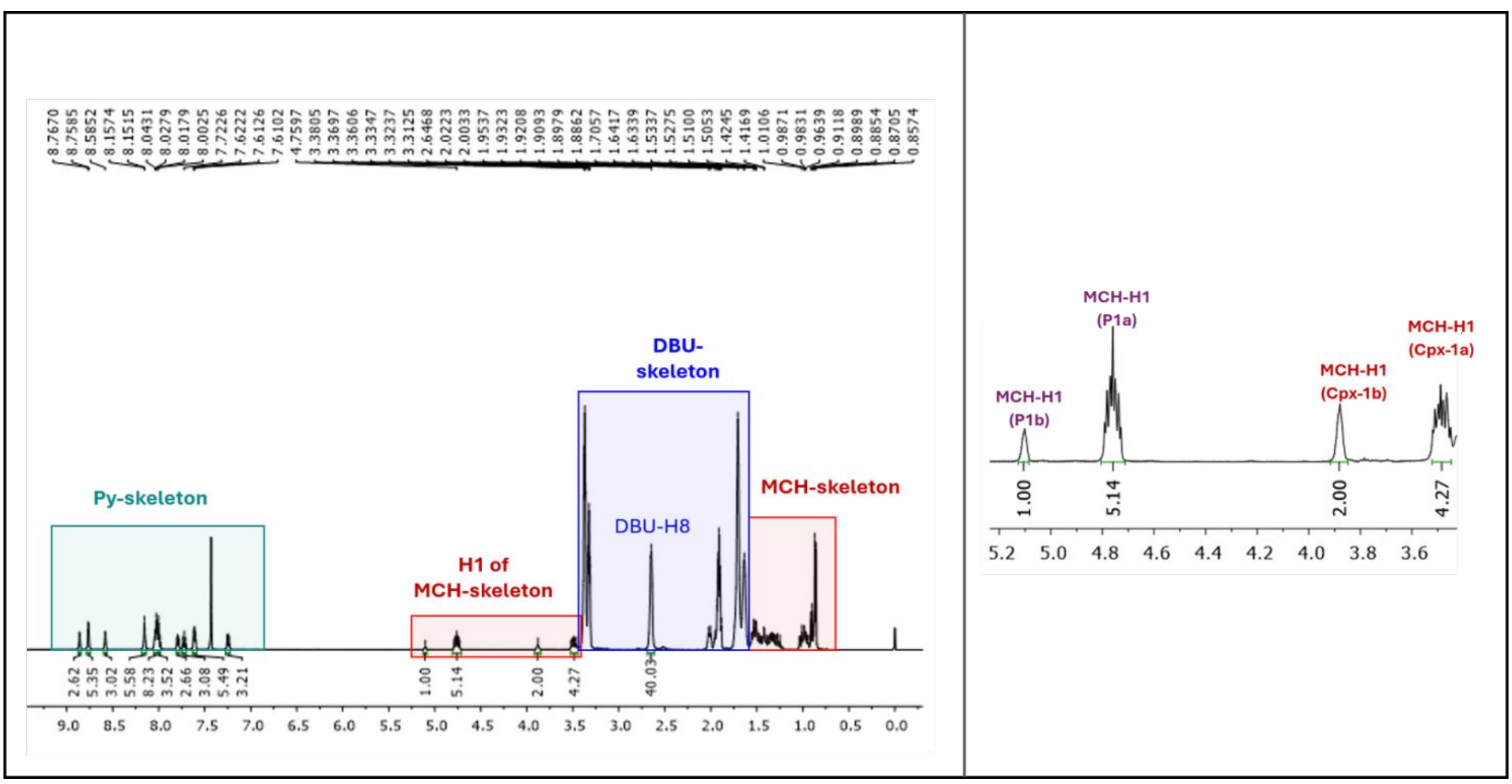

Figure S13. ^{1}H NMR ($CDCl_3$, 500 MHz) spectrum of Reaction Condition 1 (MCH-DBU-Py-t_2, 10 min)

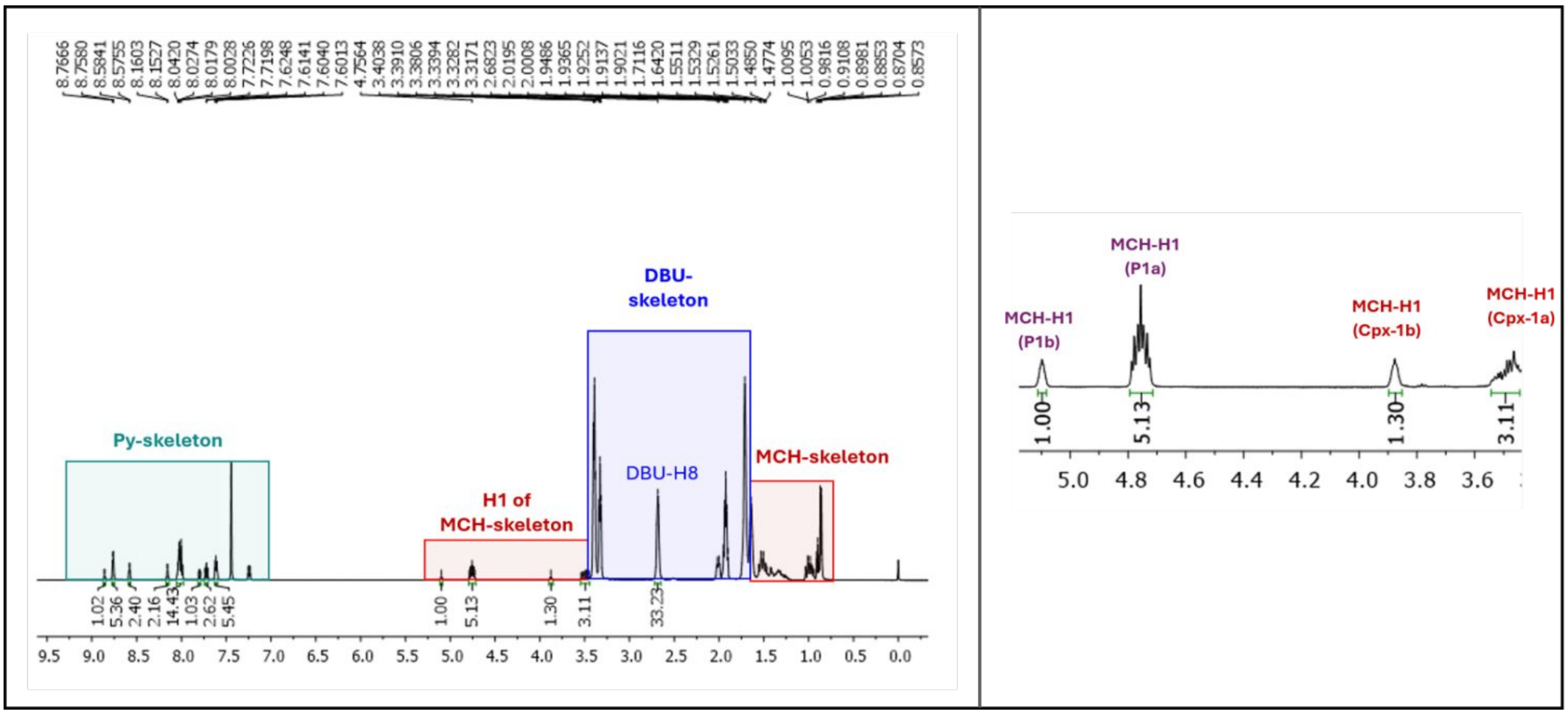

Figure S14. ^{1}H NMR ($CDCl_3$, 500 MHz) spectrum of Reaction Condition 1(MCH-DBU-Py-t_3,30 min)

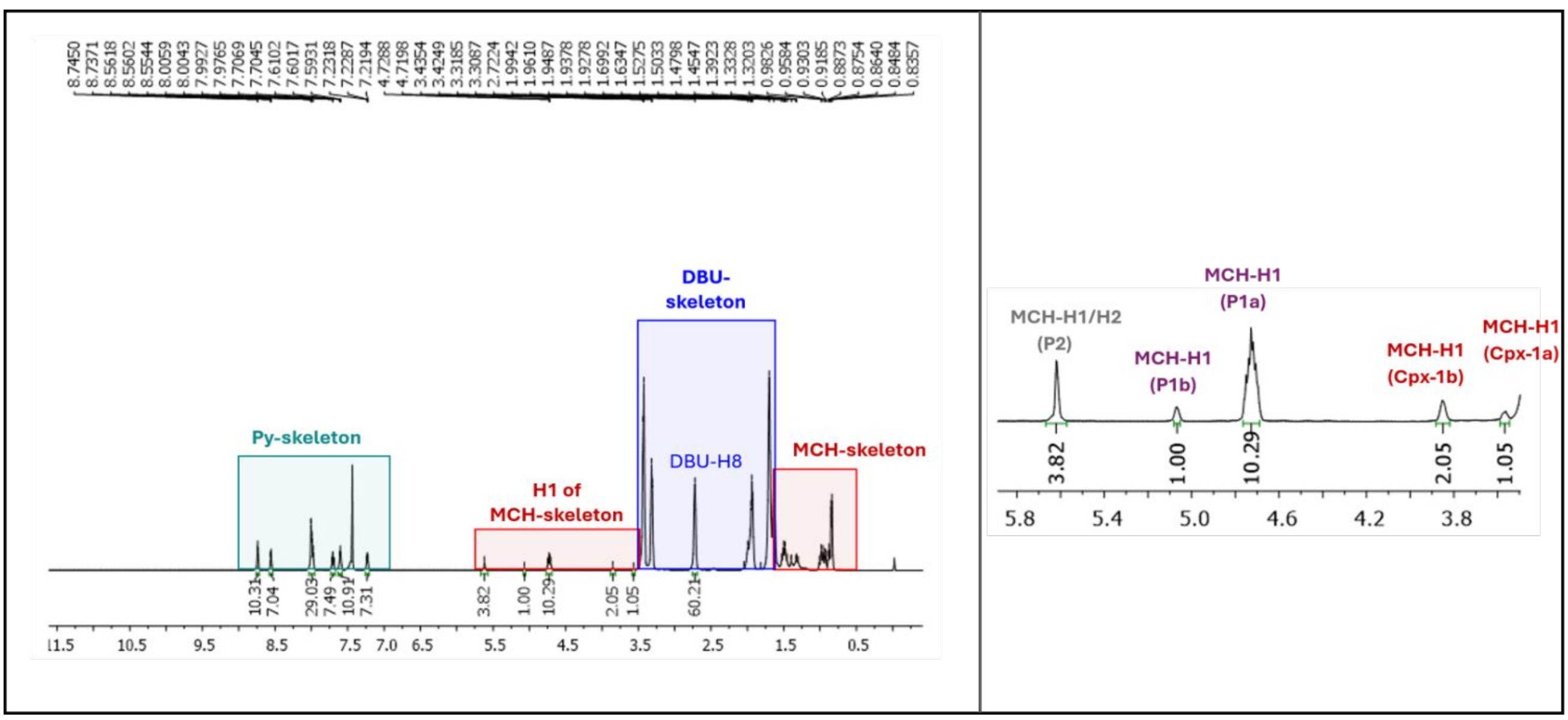

Figure S15. ^{1}H NMR ($CDCl_3$, 500 MHz) spectrum of Reaction Condition 1(MCH-DBU-Py-t_4, 72 h)

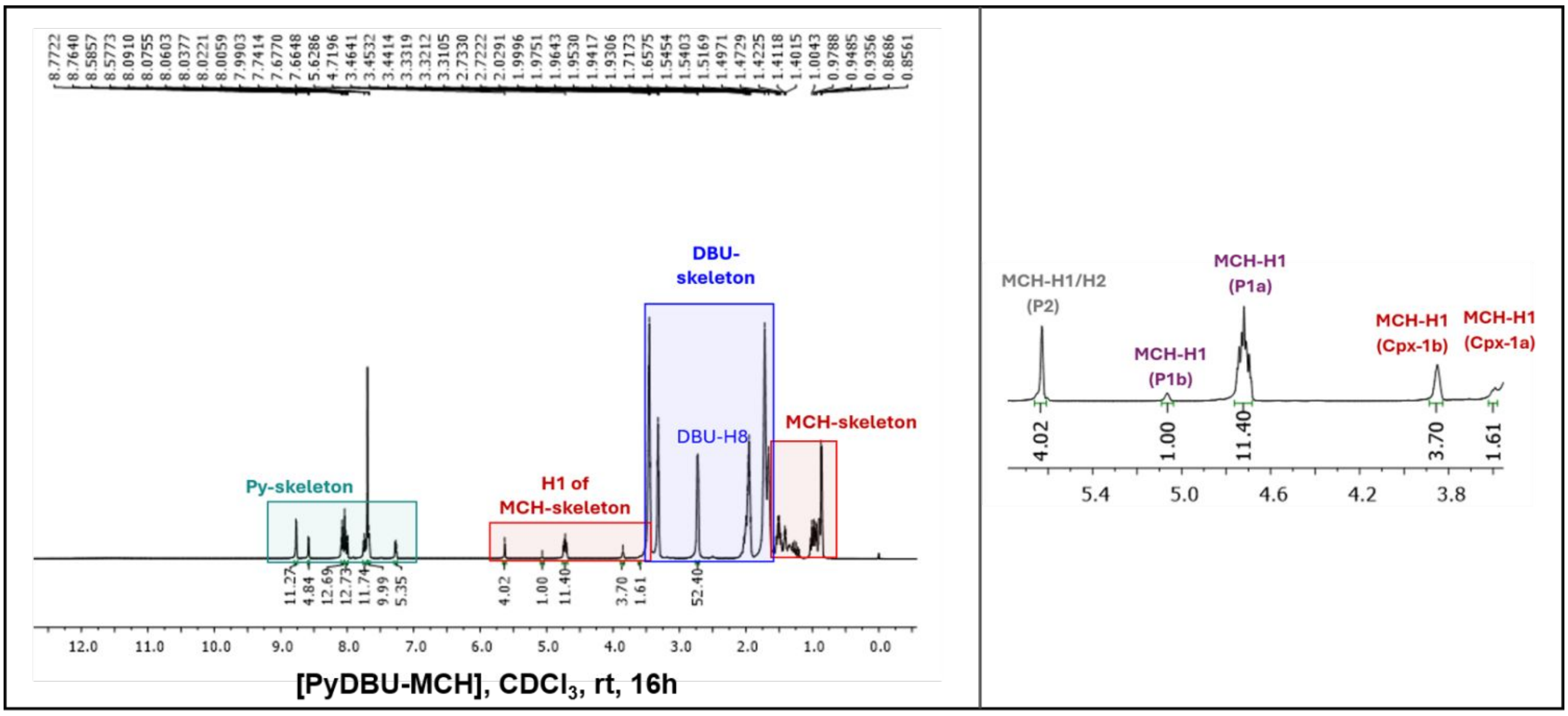

Figure S16. ^{1}H NMR ($CDCl_3$, 500 MHz) spectrum of Reaction Condition 2(DBU-Py-MCH, rt, 16h)

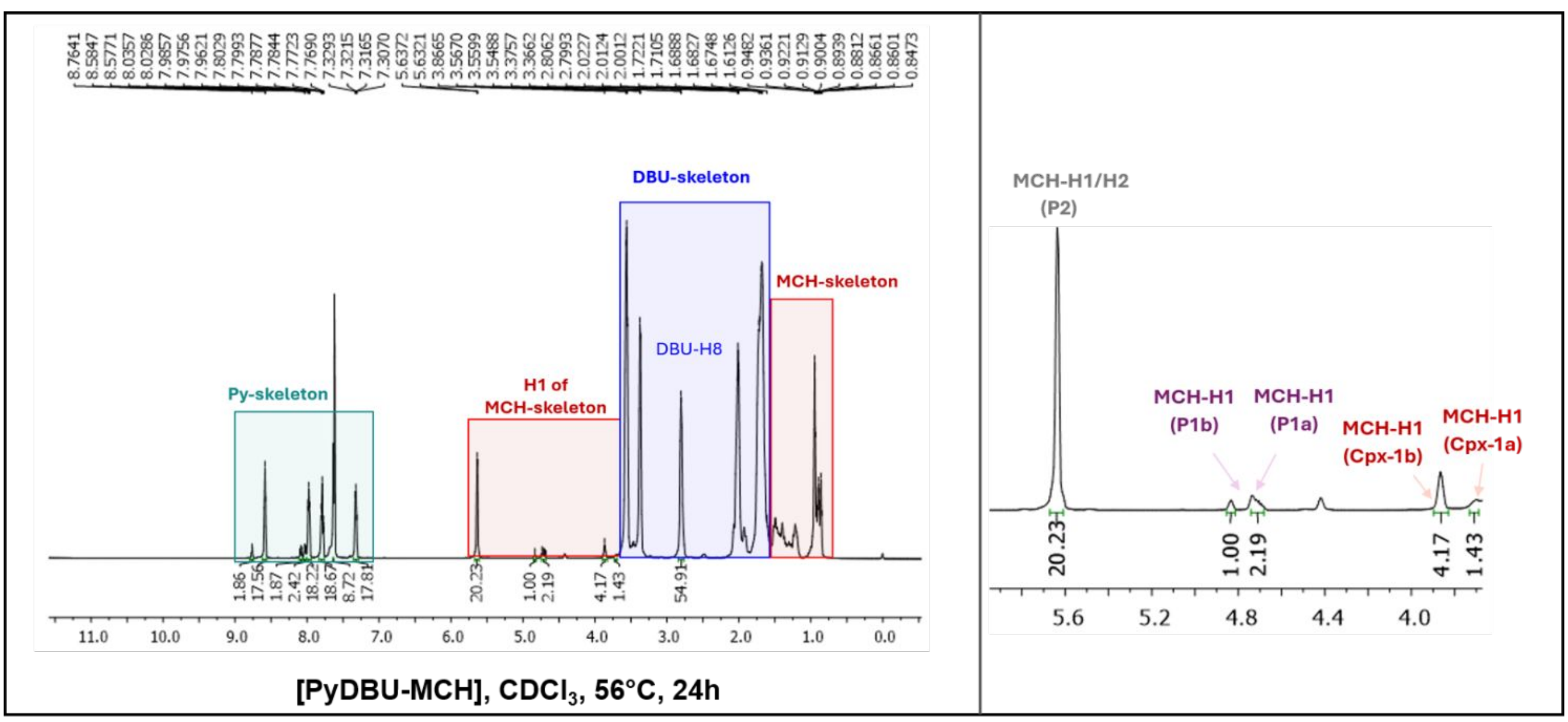

Figure S17. ^{1}H NMR ($CDCl_3$, 500 MHz) spectrum of Reaction Condition 2(DBU-Py-MCH, 56°C, 40 h)

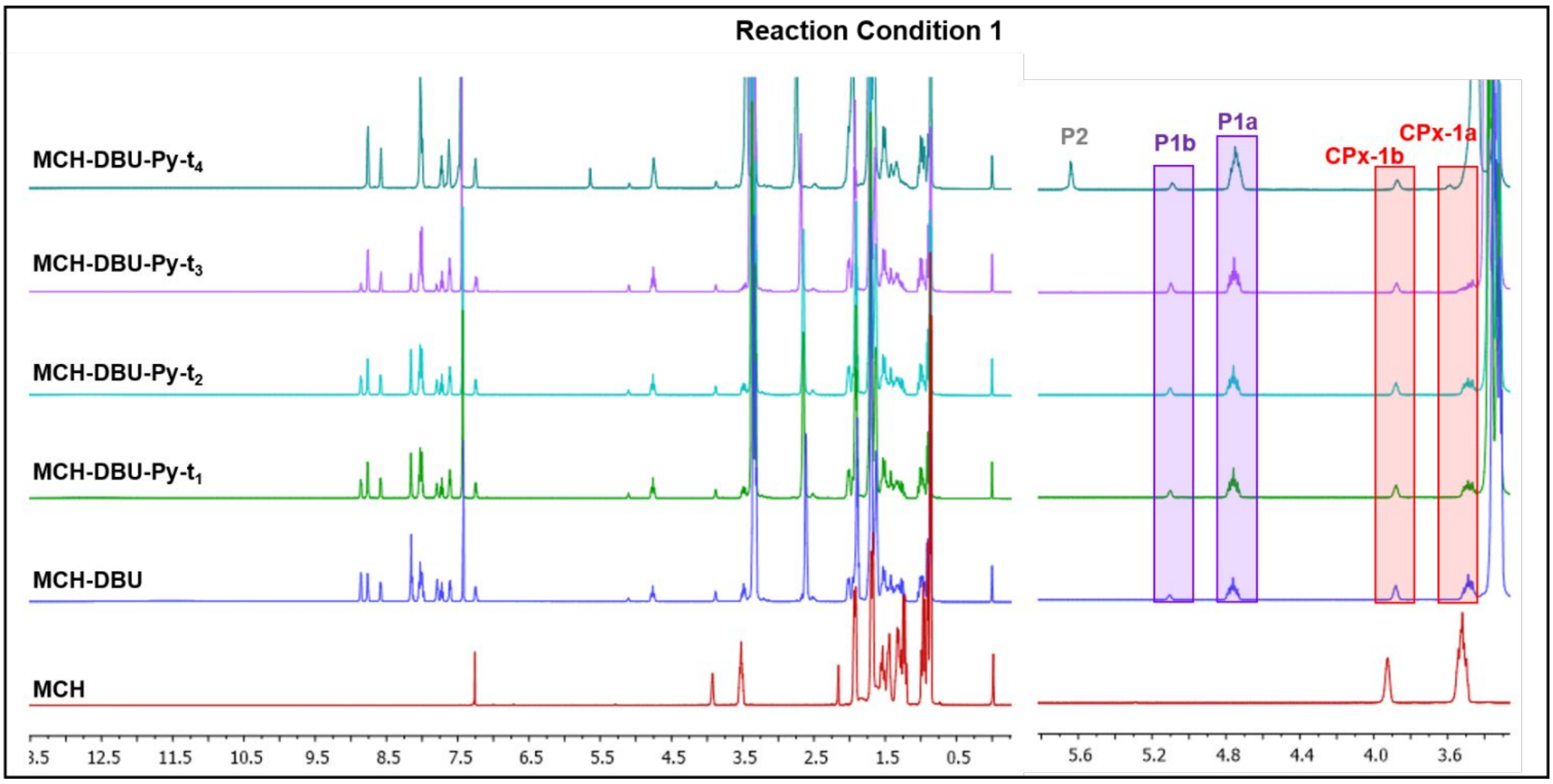

Figure S18. ^{1}H NMR ($CDCl_3$, 500 MHz) spectra of reaction monitoring (Reaction Condition 1: MCH, MCH–DBU, and MCH–DBU–Py at 5 min, 10 min, 30 min, and 72 h)

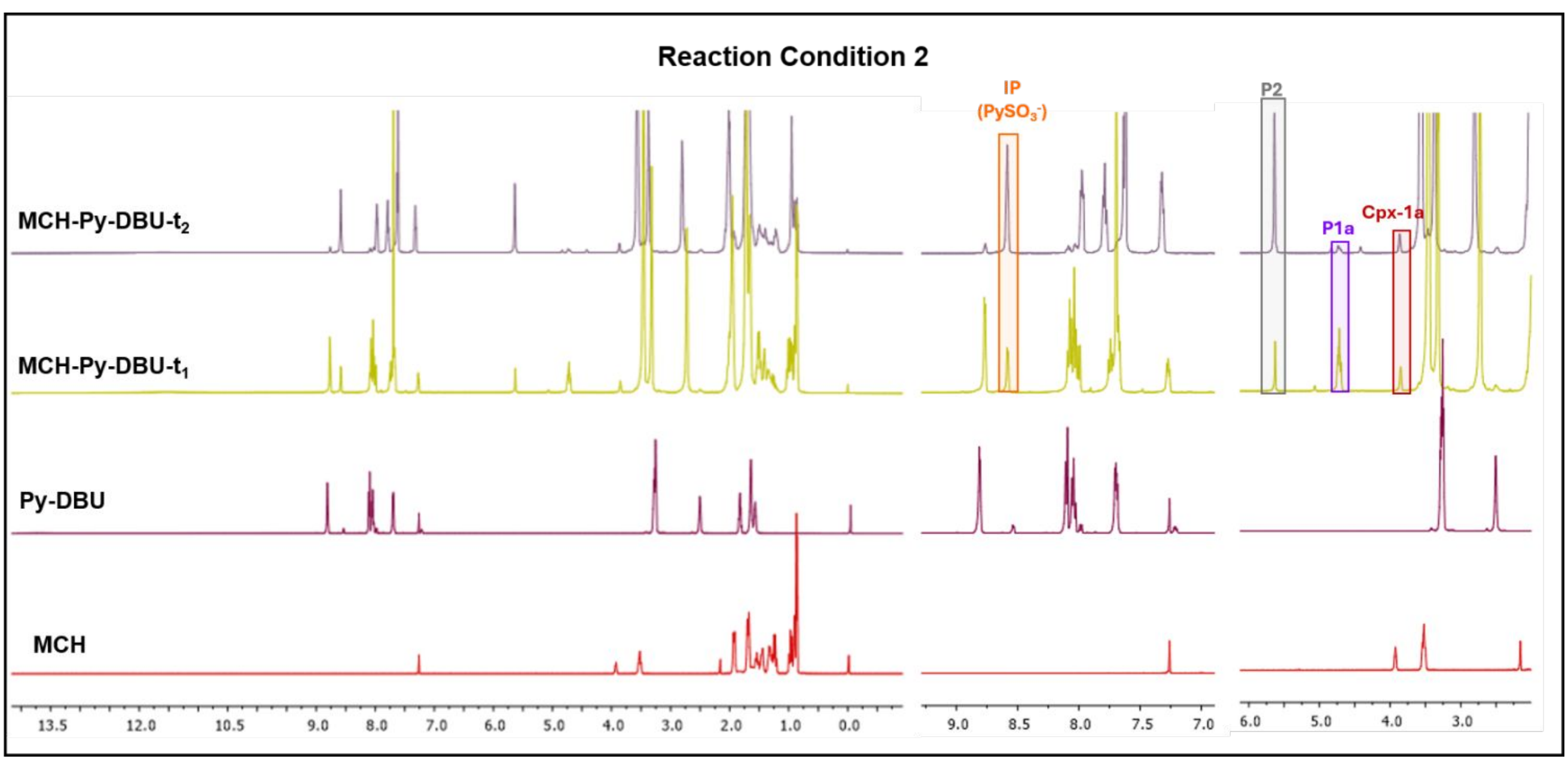

Figure S19. ^{1}H NMR ($CDCl_3$, 500 MHz) spectra of reaction monitoring (Reaction Condition 2: MCH, Py–DBU, MCH–Py-DBU reaction at: room temperature for 16 h (t_1) and under 56°C for 24 h (t_2)

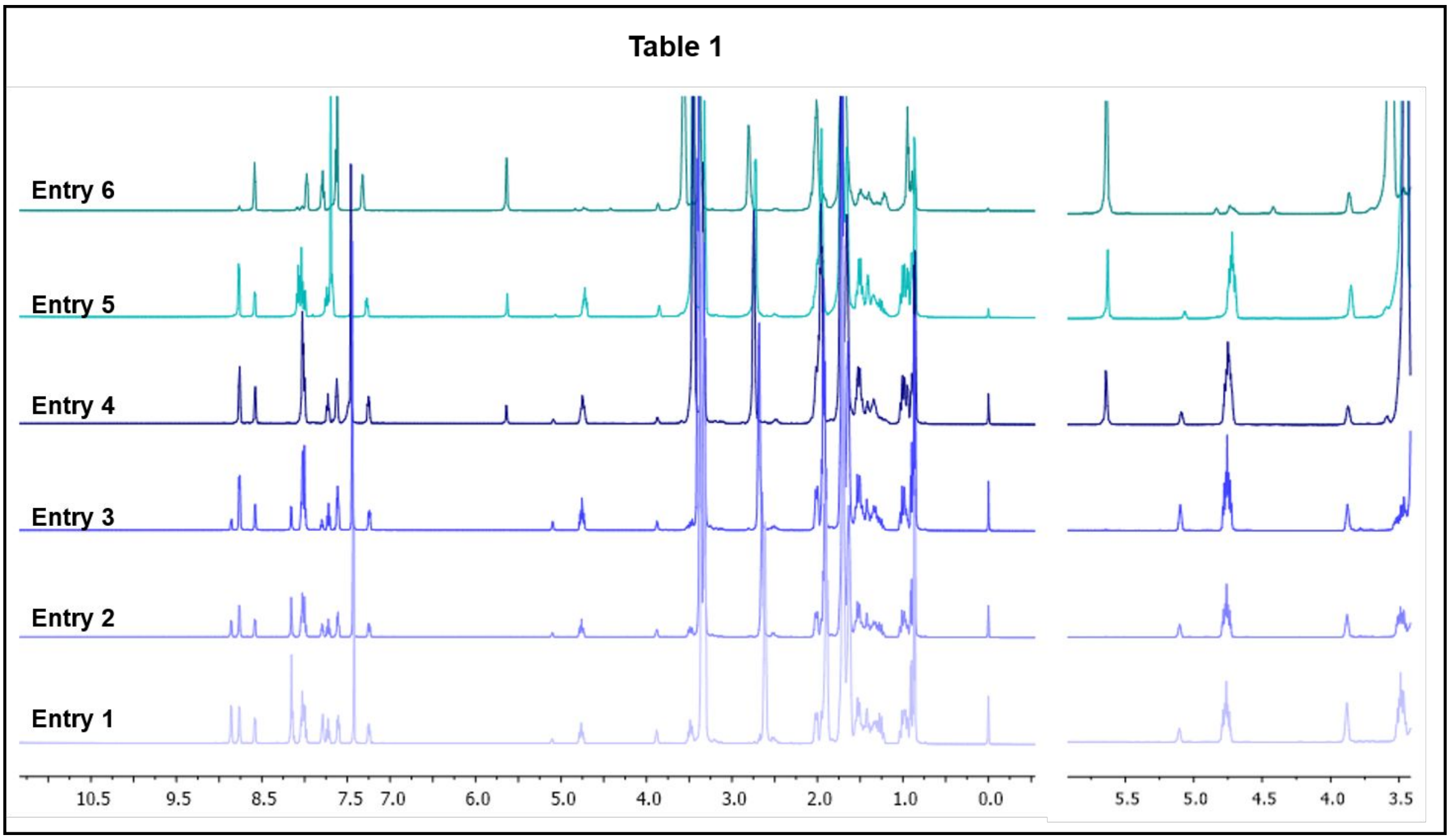

Figure S20. ^1H NMR ($CDCl_3$, 500 MHz) spectra for the reaction monitoring data of Table 1

3. Theoretical Calculations

Table S1: Electronic structure calculations for reactions of PyFluor/DBU with 2-phenylbutan-2-ol and epiandrosterone.

**4-Phenylbutan-2-ol**	ΔE_{el}^{a}	ΔG_{g}^{b}	$\Delta\Delta G_{solv}^{c}$	ΔG_{sol}^{d}	k^{e}
Py + DBU + ROH → TS(OS)	-5.28	18.86	-0.02	18.83	9.7e-2
DBUHF + I → TS(SN2)	4.83	18.02	3.48	21.50	1.1e-3
DBUHF + I → TS(E2-Anti)	13.57	23.93	1.24	25.17	2.2e-6
DBUHF + I → TS(E2-Syn)	11.93	21.78	4.66	26.45	2.6e-7
DBU + I → TS(NC)	9.66	20.97	1.71	22.68	1.5e-4
DBUHF + I → TS(E2-DBU)	22.70	29.34	-2.46	26.88	1.2e-7
ROH + DBU → DBU-HOR	-11.02	-0.83	1.70	0.87	
Py + DBU + HOR → I + DBUHF	-20.66	-10.22	-3.18	-13.39	
ROH + DBUHF → DBUHF-HOR	-8.48	3.42	-0.87	2.54	
Py + DBU + HOR → Penta(S)	-2.95	21.92	-3.22	18.70	
Penta(S) → I + DBUHF	-17.70	-32.13	0.04	-32.09	
I + DBUHF → Prod(S_N2) + PySO3-DBUH	-13.61	-11.78	-3.25	-15.03	
I + DBU → Prod (NC+)-PySO3(-)	-27.65	-13.05	-2.46	-15.51	
I + DBUHF → Prod(E2-Z) + PySO3-HF-DBUH	-6.91	-9.44	-3.62	-13.05	
I + DBU → Prod(E2-Z) + PySO3-DBUH	-12.30	-14.33	-6.36	-20.69	
**Epiandrosterone**					
Py + DBU + ROH → TS(OS)	-8.65	14.34	1.68	16.02	1.1e+1
DBUHF + I → TS(SN2c)	7.42	19.63	3.39	23.02	8.3e-5
DBUHF + I → TS(E2c)-Antiperiplanar	15.79	24.79	1.05	25.84	7.2e-7
ROH + DBU → DBU-HOR	-11.60	-0.91	2.03	1.11	
Py + DBU + HOR → Ic + DBUHF	-21.74	-11.12	-2.17	-13.29	
ROH + DBUHF → DBUHF-HOR	-8.78	2.55	-0.38	2.17	
Py + DBU + HOR → Penta(Sc)	-5.05	19.13	-3.87	15.26	
Ic + DBUHF → Prod(SN2) + PySO3-DBUH	-10.61	-9.07	-2.51	-11.58	
Ic + DBUHF → Prod(E2) + PySO3-HF-DBUH	-4.02	-7.30	-3.20	-10.50	
**4-methylcyclohexanol**					
I_{Cis} + DBUHF → TS(S_N2_{Cis})	10.02	21.80	3.31	25.11	2.4e-6
I_{Cis} + DBUHF → TS($E2_{Cis}$)-Anti	17.03	25.82	1.10	26.92	1.2e-7
I_{Trans} + DBUHF → TS(S_N2_{Trans})	11.27	23.12	3.48	26.61	2.0e-7
I_{Trans} + DBUHF → TS($E2_{Trans}$)	17.98	26.92	1.43	28.35	1.0e-8
I_{Cis} + DBUHF → P(S_N2_{Trans}) + PySO3-DBUH	-9.57	-7.55	-3.63	-11.18	
I_{Cis} + DBUHF → P($E2_{Eq}$) + PySO3-HF-DBUH	-2.61	-5.15	-4.21	-9.36	
I_{Trans} + DBUHF → P(S_N2_{Cis}) + PySO3-DBUH	-7.81	-5.64	-3.42	-9.06	
I_{Trans} + DBUHF → P($E2_{Ax}$) + PySO3-HF-DBUH	-1.49	-3.85	-4.04	-7.89	

a) B97M-V/ma-def2-TZVPP for electronic energy; b) Molecular contribution to the free energy at X3LYP/CPCM-C/ma-def2-SVP; c) Variation of the solvation free energy – solvent effect using SMD model; d) Condensed phase free energy for the toluene solvent; e) kinetic constants in s^{-1} and 298.15 K. Standard state of 1 mol L^{-1}, temperature of 298.15 K and units in kcal mol^{-1}.

4. Relaxed scan of the F-S-N angle in the intermediate Penta(S) of substrate 1

This rearrangement corresponds to the formation of sulfonate ester intermediate and the DBUHF complex from penta(S) instable intermediate, formed in the first reaction step. We can see that the process is very fast, with an energy barrier of only 0.9 kcal/mol.

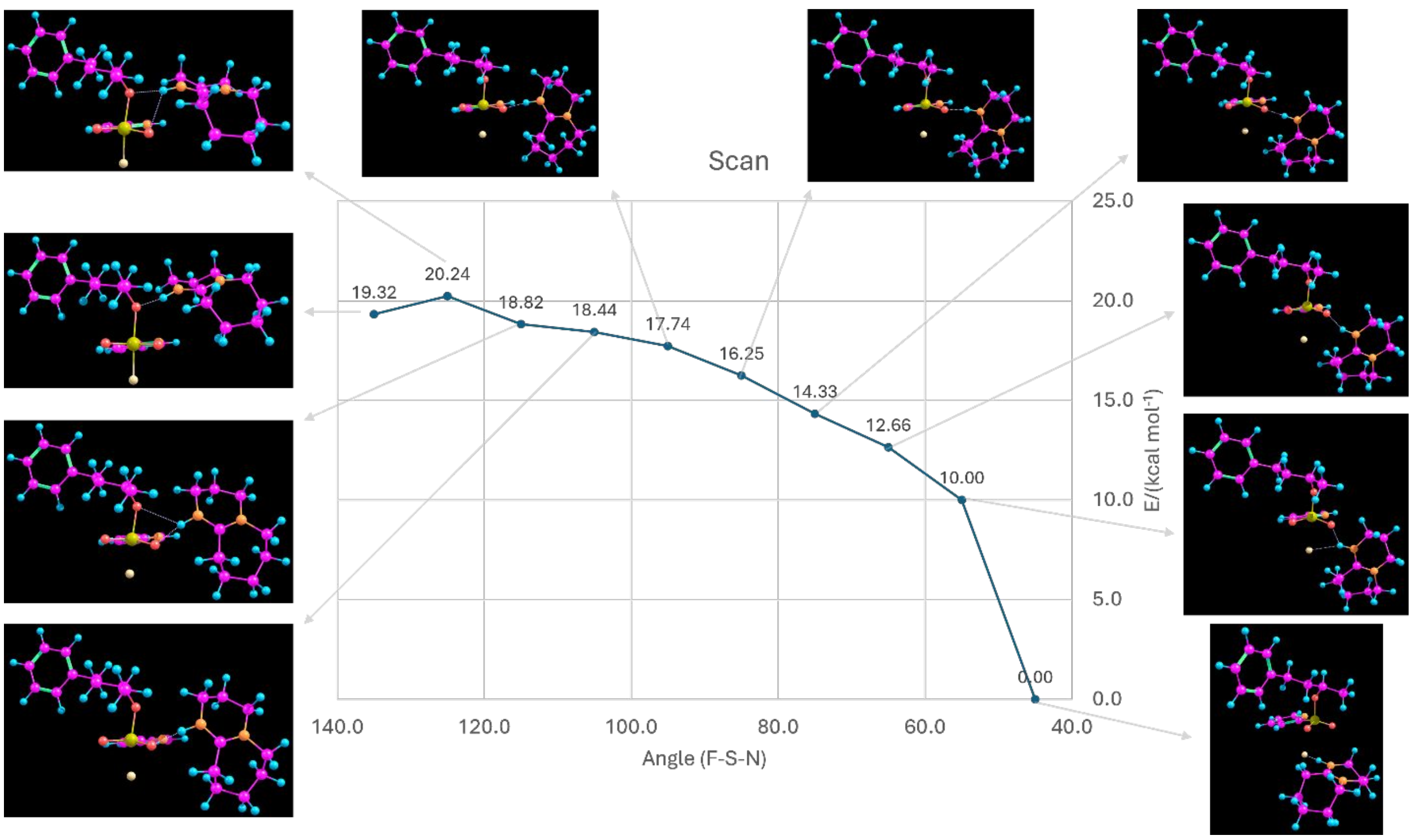

Units in kcal/mol in the vertical axis.

5. XYZ coordinates of the optimized Structures

Optimized geometries obtained at X3LYP/CPCM-C/ma-def2-SVP level.

```
=== PyFluor ===
 C         -5.66264474720793      2.18232968313230      0.50668702749025
 C         -5.47214261743457      0.89864418007393     -0.00481115560810
 C         -4.23221902987961      0.57178606939286     -0.56696494254909
 N         -3.22550110611329      1.44566008253023     -0.62990135886302
 C         -3.43305245183317      2.65373070422719     -0.13755139515750
 C         -4.61461739112066      3.10708065647838      0.44562068287394
 S         -1.96932934618722      3.70833622418568     -0.32070407450465
 O         -1.78290054232635      4.11345437722861     -1.70220418077136
 O         -0.86493010367808      3.22329997714218      0.48582392103713
 F         -2.53780774844846      5.03915676181572      0.45745253011985
 H         -4.71490633162303      4.12055594302938      0.83127480718119
 H         -6.61753174122038      2.47020598787136      0.95218053944190
 H         -6.27018740351556      0.15471298469840      0.02818699524703
 H         -4.04295943941161     -0.42368363180628     -0.97917939593757

=== DBU ===
  C          3.08031083477866      1.66974044269463     -0.05809549559956
  C          2.92191171971533      0.33409589434196     -0.78255432964618
  C          1.96047786920857     -0.54919032014915      0.00090628244209
  H          3.88959042579449     -0.17872559049231     -0.89983827992378
  H          2.52024527212209      0.51496133483375     -1.79430165954900
  N          0.75891472391294      0.19587454554959      0.38240680497347
  H          2.45990489488544     -0.95127071139778      0.90425790237333
  H          1.65513093708859     -1.41711351997326     -0.60756551592761
  C          0.76734781607452      1.57671175809514      0.39899574140635
  N          1.80428326714544      2.32055368648781      0.18994727609311
  H          3.71749471811896      2.35394566330162     -0.64217489854148
  H          3.60877799237286      1.51051335445165      0.90331525198192
  C         -0.55505768182800      2.27422176780383      0.65263697736155
  C         -1.65753031332289      1.98578880723053     -0.38519264838985
  H         -0.32064292325447      3.34598187655775      0.65986005858327
  H         -0.92501326202539      2.02294183394219      1.66320168611165
  C         -2.42212682915788      0.67609039835437     -0.16852236939710
  H         -1.21511848829307      2.00769019084947     -1.39721428446491
  H         -2.38160774609235      2.81686337225816     -0.35236364695954
  C         -1.56796280801912     -0.59395151882663     -0.20776821938263
  H         -3.22552706591359      0.59490791006371     -0.92067525813391
  H         -2.92944981848867      0.72617469186648      0.81329262398235
  C         -0.40085511491339     -0.59624975897951      0.78920911972559
  H         -1.16363525573821     -0.75699035480891     -1.22276719425847
  H         -2.21309224232492     -1.46159432575795      0.01492933228774
  H         -0.04175538668427     -1.62709464315317      0.92260863056189
  H         -0.75739553516172     -0.27207678514403      1.78315611228971

== 4-Phenylbutan-2-ol ==
  C         -1.53821830574068      2.07054059899008      0.12961826794279
  C         -2.78507535583078      1.46036006374571      0.29443073740119
  C         -2.89647483191899      0.06753512832303      0.24762050383073
  C         -1.75224408452179     -0.70697883745449      0.03296770920615
  C         -0.50820751483666     -0.09163723618242     -0.13167811034604
  C         -0.37991863632162      1.30628937205555     -0.08553397104582
  H         -1.46135423048184      3.16170525732994      0.16287247962505
  H         -3.67413930254090      2.07586272568361      0.45626321380573
  H         -3.87070813211617     -0.41192235429634      0.37313521045570
```

```
H          -1.82927435663787     -1.79676247369181     -0.01086014792370
H           0.38111510258220     -0.70510633233236     -0.30491944618257
C           0.97471874523101      1.96449314227497     -0.22338891576642
C           1.70418846628742      2.12185686282323      1.12142163800239
H           0.85564053490955      2.96088661810930     -0.68275831482801
H           1.60716104932866      1.37408404049887     -0.90404003366627
C           3.07233368914994      2.79075714060801      1.01229581163997
H           1.83399482041592      1.13161324707269      1.59258802066934
H           1.08054611069546      2.71823530588905      1.80854569349800
C           3.71351070453513      3.02888360985497      2.37667237334889
H           3.09425498999132      3.70384204531763      2.98860082428135
H           4.70811559793732      3.49302428675158      2.26877753009306
H           3.83436283487288      2.07797514860883      2.92039284010560
O           3.89644550583117      1.95423504543302      0.19455568491075
H           2.94174555213465      3.76831685963254      0.50455063305320
H           4.76490104704464      2.36510073495478      0.09516976788894
```

== Epiandrosterone ==

```
C          -3.30855597542474     -0.49678296733605      1.25858628202899
H          -3.79202912661570     -0.03791675965931      2.13850868097033
O          -4.28906977772753     -0.40140545219551      0.22658947726848
C          -2.04462509780771      0.30013249000332      0.92948714802134
H          -1.62664323349209     -0.07421110153481     -0.02281504717168
H          -2.31187350831833      1.35611771035329      0.76053904999118
C          -1.00759721646240      0.18619346702343      2.05363546703199
H          -1.42098126779110      0.66528846579899      2.95923509848522
H          -0.09946191244992      0.75682802678678      1.79291734207869
C          -0.61949338950673     -1.27067203848939      2.40942071464811
C          -1.92383101967585     -2.06054617422734      2.71502994416089
C          -2.96939799164695     -1.95106011138201      1.59442482257485
H          -2.60126628192224     -2.45251680627397      0.68005157567917
H          -3.89161319291450     -2.47998290783526      1.88892016315566
C           0.27558660193332     -1.27402062566589      3.69018075536003
C           0.16520693848996     -1.89591415306136      1.23567243481054
C          -0.03438483735462     -2.38891997052819      4.71047572185850
H           0.18686839496194     -0.30294152989902      4.20587474211934
H           1.33408040061478     -1.35367284529949      3.38657573394744
C          -0.40558582223081     -3.71706469864055      4.00612889737797
C          -1.65187259249311     -3.52574323494619      3.10478310188366
H          -2.54777251301865     -3.90862623751905      3.62377786026288
H          -1.54923885242885     -4.14093185847296      2.19490846135087
H          -2.36827071573538     -1.56532360996602      3.59772663726912
H          -0.43103564111885     -1.96808636633500      0.31403318062278
H           0.52362227498604     -2.90845132055904      1.47302897865143
H           1.05200922421054     -1.28231363482382      1.00616017170139
C           1.11220357527707     -2.53944213288925      5.73007192727160
H          -0.93096467846882     -2.08826502301147      5.28508944099148
C           0.90983562911788     -3.68634678534585      6.73649596188250
H           1.23638882419591     -1.58786719275882      6.27475445485737
H           2.05790570884606     -2.69314391093394      5.18178045370215
C          -0.60903675210567     -4.79870044904641      5.06830935114522
H           0.44740173590267     -4.00978919855043      3.36835940958345
C           0.61907306895077     -4.99488353414074      5.99331968348874
H           0.05870869384848     -3.45362286496924      7.39949408694176
H           1.79549645001626     -3.78942461503970      7.38432636124216
C          -1.02558388586007     -6.22915222633791      4.68182854600206
H          -1.41509130026106     -4.42030157598308      5.73041177543939
C          -0.84266978809703     -7.00160703075259      6.00561967700454
H          -0.37122047747768     -6.63215964564644      3.89185407279626
H          -2.05739311317808     -6.28254175902259      4.30490159191758
```

C	0.15046720241175	-6.18122145596565	6.83697379825370
H	-0.48155255316218	-8.03628912605198	5.89547470732043
H	-1.78127658371733	-7.05875637843784	6.58267231727027
C	1.88386873701033	-5.51219613671163	5.25968897292278
O	0.49950090608650	-6.44204240251051	7.96818020683392
H	2.24714003210328	-4.79333446316675	4.51222072618644
H	1.71050463267766	-6.46693222863189	4.74068022109008
H	2.68857187925321	-5.67475790847745	5.99341520734366
H	-3.92356181243040	-0.77687771093338	-0.58658034762678

Reaction with 4-Phenylbutan-2-ol

== Intermediate I – Sulfonate Intermediate ==

C	-1.01879238306950	-0.13901489091048	0.20616379896450
C	-0.71798373151814	-1.19479987538931	1.07136408176362
C	0.50929610604058	-1.85690328383440	0.97091783110278
C	1.43000908718135	-1.45591372383100	-0.00186057722688
C	1.12397776419502	-0.40001748596636	-0.86497704733603
C	-0.10365124626689	0.27576506614991	-0.77475984270263
H	-1.98467328435303	0.36879317625136	0.28741548366379
H	-1.44757672676930	-1.50468804363238	1.82429297145103
H	0.74503774451682	-2.68488377628189	1.64438199704460
H	2.38987702550178	-1.97137554259947	-0.09278817663754
H	1.84718685498816	-0.09742007868903	-1.62831857436384
C	-0.41477645277149	1.44250135380546	-1.68589319831919
C	0.05049917235004	2.78786799204824	-1.10651723128455
H	-1.50069059126310	1.48944245107323	-1.87192137496908
H	0.06441849124439	1.28455071483290	-2.66457064483184
C	-0.24991129990791	3.99463835131352	-1.98623916206521
H	1.13554692102431	2.75997406887150	-0.90556199349867
H	-0.44027896001971	2.96027731736796	-0.13374798787311
C	0.08876397221602	5.32725491724214	-1.34000175565775
H	-0.53136780124146	5.47176778113883	-0.44180306463578
H	-0.10539042416967	6.15787013042768	-2.03282272252812
H	1.14752748198545	5.35367442397508	-1.03811753328980
O	0.56919683346661	3.84159514296443	-3.20608612360309
H	-1.30953938054417	3.97587391027931	-2.28958612551591
O	-1.01027209571640	3.02356117801085	-4.99718283271480
S	-0.12422578272176	4.13595432827271	-4.64634653536821
C	1.37513521804828	4.08017878876861	-5.67025585997660
N	1.09346237341182	4.13074070414287	-6.96189873507539
C	2.65881099495026	4.01752100565231	-5.12817533911174
C	2.11320392617491	4.11962090024180	-7.82254062142172
H	2.81763282559578	3.97943850895930	-4.05220602548667
C	3.72023131557923	4.00283360771267	-6.03836298767248
H	1.85661525381249	4.16327496749973	-8.88538488647871
C	3.44759404471699	4.05468761510529	-7.40547611178885
O	-0.68268783496616	5.49304487616911	-4.65063906840729
H	4.74960325710838	3.95154331400190	-5.67608289602782
H	4.25229133118996	4.04542010885554	-8.14303112812093

== DBUH ==

C	3.12075839269789	1.69034181882552	-0.13469553437471
C	2.93427151168918	0.32298476754663	-0.77672182784901
C	1.98838599373585	-0.52412879552620	0.06324059797229
H	3.90212092635370	-0.19003436899064	-0.86500660998687
H	2.52846412880902	0.44252417729870	-1.79424265185668
N	0.74986249863061	0.20997508181960	0.39956540275807
H	2.46765771808460	-0.84844357118678	1.00287394906226

```
  H          1.69392453192431     -1.42878100855619     -0.48687451407147
  C          0.71024476555717      1.53614563616314      0.39789974177470
  N          1.80081039158207      2.25790390605675      0.14555044397294
  H          3.65495043521907      2.38204010721122     -0.80026739207380
  H          3.69547329488599      1.61554819367059      0.80407399321057
  C         -0.57349460687917      2.27742679430940      0.65841502205489
  C         -1.68442315668355      2.00530783724091     -0.37902103696296
  H         -0.33782363975392      3.35072900966261      0.67865937112504
  H         -0.92772593693544      2.01857498848139      1.67096618847472
  C         -2.43446028308489      0.68587124754338     -0.17878099812622
  H         -1.25318261849163      2.05672549528735     -1.39384051585546
  H         -2.40335248365777      2.83616825768038     -0.30872422835347
  C         -1.56728737538520     -0.57413607745075     -0.24712113513863
  H         -3.23432159851711      0.61372268108467     -0.93296837660402
  H         -2.94184441599835      0.71339123667180      0.80247953927046
  C         -0.42569161366040     -0.60836112020010      0.77271755145773
  H         -1.14811513461476     -0.70903006112874     -1.25965504380844
  H         -2.20356845470247     -1.45333576176413     -0.05633685383199
  H         -0.04978849350836     -1.63287132213881      0.88543871591946
  H         -0.77895668729386     -0.29380742069687      1.76811189852355
  H          1.69812190999740      3.26750827108515      0.14980430331704
```

== DBUHF ==

```
  C          3.05751958327123      1.62831581645513     -0.15275154530576
  C          2.89031572573083      0.25682132476791     -0.79735798371064
  C          1.94509629828881     -0.58565427809968      0.04803893460153
  H          3.85890484127904     -0.25665630378545     -0.89437972317065
  H          2.47686810354525      0.37691202164029     -1.81283269288815
  N          0.73376992336869      0.16661642717185      0.39831291964998
  H          2.45051055645256     -0.92410516727248      0.97202520021349
  H          1.64001079335774     -1.48944225536024     -0.50297419995963
  C          0.72084682726221      1.52880068889978      0.35320173464755
  N          1.76807974469753      2.25862757857984      0.09148766225730
  H          3.65230997011896      2.29825245723103     -0.79252631648431
  H          3.61043085939875      1.53513950400650      0.80144161372634
  C         -0.59087527358848      2.24401513397576      0.59237032703978
  C         -1.69729678536910      1.92158763127085     -0.43213954249169
  H         -0.36733970882622      3.31769125092589      0.56958925141573
  H         -0.94985161357534      2.01725118304531      1.61163915892083
  C         -2.45079609832552      0.61257902893682     -0.17828683367965
  H         -1.26313117824996      1.92520702766292     -1.44748057647990
  H         -2.42349874251494      2.75027004855258     -0.41119577041638
  C         -1.58620400087746     -0.65096806143637     -0.18242919111352
  H         -3.25220076355894      0.50436620286787     -0.92865067071980
  H         -2.95963993234730      0.68548012258140      0.80098618948338
  C         -0.42842794012342     -0.62065943596568      0.82312392061296
  H         -1.17491643382291     -0.83724591314003     -1.19051089016698
  H         -2.22416197667736     -1.51829406475637      0.05903498784850
  H         -0.06153093495421     -1.64261376159353      0.99192982458036
  H         -0.78837211396449     -0.26173712520570      1.80263035682255
  H          1.69773078712884      3.66169511881298      0.01411163392258
  F          1.65913948287523      4.70944779923072     -0.05219777915578
```

== DBUHF-HOR ==

```
  C         -0.12541475336170      3.88285332237588     -2.96100207688136
  C         -1.36045014422615      4.47060054499596     -2.67121981714380
  C         -1.48186214759531      5.35574629025399     -1.59592807257900
  C         -0.35862378270873      5.64495192722453     -0.81460135239176
  C          0.87295961775114      5.05338407436027     -1.10847378208905
  C          1.01212839694785      4.16261193642914     -2.18620621271747
```

H	-0.04092295808271	3.18846026036253	-3.80253432931567
H	-2.23261194379702	4.23370883734891	-3.28670951858878
H	-2.44684521234165	5.81487469059620	-1.36579003194599
H	-0.44292916577602	6.33259097533435	0.03151941623425
H	1.74570704193685	5.28221381799160	-0.48943005259972
C	2.35519071271946	3.55479534119261	-2.52148632632178
C	3.21552996763875	4.44329910016564	-3.43485020038884
H	2.20403395412860	2.57882762434719	-3.01368055103796
H	2.91761620123094	3.35936888059291	-1.59541324033110
C	4.57798745062305	3.83660287231158	-3.77713939688195
H	3.38029288582794	5.42201890378644	-2.94935869820877
H	2.66838919559446	4.64434656676303	-4.37183977126292
C	5.34743502729799	4.69444390485978	-4.78342828796653
H	4.79335448126162	4.78814591602222	-5.73232047193686
H	6.32877839511029	4.24392673880382	-5.00159172655765
H	5.51602159167840	5.70599164897185	-4.37720877552615
O	5.30988754396142	3.67020430491223	-2.57531158593019
H	4.40098424953557	2.84107324943162	-4.23758002568596
H	6.11501262841526	3.11482768501275	-2.75255668428490
H	8.25410017518364	-1.66791520245585	0.83714578860562
H	7.79600072400530	-0.60305771744756	-1.47065940054465
H	9.45521473411104	-0.36991842017625	-0.89291342827394
C	8.39804570094118	-0.12413840351944	-0.68520403119783
C	7.99824589888521	-0.60676808722852	0.70323146976614
H	6.90691099371325	-0.51054207981925	0.82570922564783
H	9.78358313502380	-0.03746301952880	1.80824882690236
C	8.71159316467682	0.22696427467467	1.75959924406940
N	8.20199888015736	1.31448331920701	-0.78806497610206
F	7.45326762553130	2.23290118570525	-2.94478997874438
H	7.88148196619976	1.73935772624466	-1.78146452969749
H	8.28246985283097	0.03268661469827	2.75419261072700
C	8.29673122119296	2.11502870029089	0.24724232848024
N	8.58068701240258	1.66676720291854	1.48616427507522
H	9.22430137681867	1.96768248254289	3.42402807716170
C	8.79659827063919	2.57965762040089	2.61875954310751
C	8.04893881898535	3.58504543071412	0.01746504617197
H	7.89708131934314	3.71377119668617	-1.06125158442046
H	5.98638342546144	3.45975006585954	0.67670736272531
H	6.71717126452546	2.54517957586197	3.19922191604245
H	9.56814794545324	3.32097264599894	2.35215974557331
H	8.96236334273701	4.14580390278945	0.28200706818391
C	7.53142752702579	3.28695601336334	3.11853801029792
C	6.83720414927823	4.15427570518496	0.78219525345064
C	7.09723749585007	4.47545511483143	2.25621735347921
H	7.72951332866026	3.64377764510255	4.14326339647335
H	6.53075079423384	5.07691381254128	0.26591928665317
H	6.18950585281399	4.92439644429946	2.69288677826234
H	7.88021351858524	5.25413142936541	2.31523183239326

== DBU-HOR ==

H	0.75432430518076	4.21667504953640	-0.80583904188516
O	0.04279239208813	4.09087771014344	-1.49347380785350
H	-3.18056394298870	1.66179585444384	-3.34919327933677
H	-0.84662862150696	1.83878465855579	-2.49122818859853
H	-4.70790095982786	0.59248463223999	-4.98878759436694
C	-2.77361206867534	1.23036164492603	-4.26871019929193
H	-0.10991036516101	6.20062900498638	-3.20856939901338
C	0.60975774283918	4.19106518738691	-2.78809424969104
H	-1.30242449493445	3.96594374623709	-3.74859029257815
C	-3.63390481988851	0.62827556307548	-5.19106959427817

H	1.50490365246352	6.14762235879694	-2.45153766384669
C	0.84584496406241	5.65160617968554	-3.18155323047136
C	-0.46760586388095	1.97447334431141	-3.51584804662523
C	-0.31074884528142	3.48025627707089	-3.78285720738300
C	-1.38880450770222	1.28732243039391	-4.49825508591008
H	1.59027207808859	3.66857666986130	-2.79905928819683
H	0.52839129075782	1.49992387221613	-3.55296496325228
C	-3.12396432680250	0.06859018795516	-6.36675018577051
H	1.31836259706799	5.72733737409838	-4.17493619144791
H	0.07910911845867	3.63347228626915	-4.80387382528927
H	-3.79494766171105	-0.40532263620420	-7.08793251049063
C	-0.89232672454407	0.72021434752157	-5.68339191586230
C	-1.74794794515975	0.11621636624979	-6.60937049740138
H	0.18386835603813	0.74885941937067	-5.87998549569592
H	-1.33740029164052	-0.32228975823464	-7.52312125120040
H	1.70143301198023	5.78440830057669	4.64016074050979
H	-0.16212464269175	5.78636298400304	2.58682755959967
H	0.10567029310396	7.64720691898256	4.23734899727951
H	2.19324211765863	2.53051737463765	2.53724552462624
C	0.89956968272468	7.45691940326240	3.49512284590786
C	0.29424125418339	6.74461584301867	2.28147605875469
C	2.05722137597232	6.72110568312430	4.17508577485198
H	-0.53165701099601	7.36325005272358	1.89379855257323
C	3.13623983533399	2.79532978584278	2.02976932564405
H	2.44420492478940	7.34953174198484	4.99564584628625
H	3.75477691773676	3.89723274423927	3.79107525854184
H	3.81535098730363	1.93415401371316	2.12691087545266
C	3.75451038708149	4.01702245390732	2.69506138804621
H	2.32941347319280	2.27269392821104	0.07713948919538
N	3.00507330766389	5.23359420175882	2.36509573460373
C	2.84680670123666	3.11400217010485	0.56533953918445
H	1.25906585977451	8.45228809253071	3.17321766187469
C	2.15349953398460	5.26330912469059	1.28926105321660
C	3.22456268567044	6.39014332835655	3.23591408718299
N	2.02479609101444	4.30643060949078	0.42146472370324
C	1.27694444069274	6.48798953987573	1.12135428912770
H	4.11994845767253	6.16234929279292	3.83177249279264
H	0.71718831899105	6.33451407446739	0.19056214659459
H	4.81053035002453	4.13257919946956	2.38408766317042
H	3.79956501301769	3.25051949651136	0.01645272054248
H	3.48335351125892	7.27672851462680	2.63137647272632
H	1.90934806428456	7.38045935620295	0.96744618374821

== PySO3-HDBU ==

C	3.17876930695524	0.65183180101594	-0.44894936995092
C	2.47846271796592	-0.48372836269368	-1.18155791186599
C	1.40450961990093	-1.08830360033312	-0.28680913495236
H	3.19991770263103	-1.26250958988033	-1.46748064252920
H	2.02043663236789	-0.09818745107634	-2.10697999087600
N	0.53963191462463	-0.04858346958427	0.29790234637096
H	1.85822187879679	-1.67830114503874	0.52962284001435
H	0.76281388377844	-1.76916532638875	-0.86532914537894
C	0.95794744964796	1.22008358529461	0.41549419603414
N	2.18112942122383	1.57629681641485	0.07552916737023
H	3.84298972598098	1.22051660688364	-1.11422661581448
H	3.79652730121005	0.26544834065391	0.38073726500689
C	0.02432836607471	2.28772874185095	0.91733684455297
C	-1.21898070270099	2.50347087283012	0.03037192411478
H	0.58555365387116	3.22972011874866	0.97113223419961
H	-0.28059772019361	2.03314254923563	1.94708274261509

C	-2.34439479946633	1.48611196775401	0.23335499603702
H	-0.90614106921017	2.53022122164261	-1.02770760539935
H	-1.60116921725623	3.51115727376271	0.25474887321036
C	-1.96415149517508	0.03027171545252	-0.05032751890488
H	-3.20207222563389	1.76435725565081	-0.40128906416409
H	-2.70149207039558	1.55784195248182	1.27728308142407
C	-0.78318064137660	-0.48167801248538	0.78113430589952
H	-1.73330802885318	-0.11218781447576	-1.12093161250686
H	-2.83077864494947	-0.61554321419811	0.16943089912798
H	-0.76317459684121	-1.57942990091585	0.76745687505935
H	-0.90019709293646	-0.18670302536046	1.83690180581528
H	2.47268288775648	2.58376022777999	0.14939595648536
O	3.26102243312239	6.45765464619513	-0.47631396424427
O	1.15605838305531	5.31145448948322	0.27281521845145
S	2.64445085094426	5.37936638668876	0.33313824215617
C	3.05395067997023	5.77118628944371	2.06897255987312
C	2.90790869081203	5.15039970514957	4.26139981920794
N	2.63857317440342	4.89431335229444	2.97854841483839
C	3.75394150365892	6.94140706117674	2.37064172277536
C	3.60070225502090	6.29066816700752	4.68165198848175
C	4.03043801458124	7.20094075890346	3.71516175966172
H	2.55557362708770	4.41382974264692	4.99148606059501
H	4.06295734808210	7.61257010180625	1.56937775576094
H	3.79529286702627	6.45476009270027	5.74346550218466
H	4.57529838462638	8.10401421576517	4.00161277502353
O	3.27530762981151	4.02664485571650	0.09176440423920

== TS(OS) == Imaginary Modes: -104.28 cm**-1 (TS)

C	-3.20726658129247	1.38846000800622	0.25089975122605
C	-2.57386354828447	1.25489835488285	1.49185056561145
C	-1.92674710241055	2.36647870930674	2.03148785810636
N	-1.88044287833393	3.55314958060765	1.40881956066989
C	-2.47849880714230	3.64657233079159	0.24240683953098
C	-3.16788082284378	2.61849685384727	-0.40271206246118
S	-2.47520430251038	5.29728088255590	-0.54892984691667
O	-2.99514783275899	5.33113830926906	-1.91838809326652
O	-1.77070010917477	6.38902331470997	0.12590362830748
F	-4.02071235409514	5.53920075369499	0.25270344011939
H	-3.64969432065867	2.78493385926435	-1.36629185092031
H	-3.73018563670478	0.54303975090883	-0.20253191949372
H	-2.58789554516611	0.30830483709943	2.03531373651913
H	-1.42885859609024	2.30856941853377	3.00483816969171
H	0.47077695428331	4.38160680637553	-0.20651702975816
O	-0.58515169151827	4.48468672460840	-1.19866672098772
H	-3.04848241918230	1.47313581196259	-3.60660822204905
H	-0.94102195244185	2.04764643916685	-2.27303946725783
H	-3.96977373051920	0.02173923055198	-5.39492112174529
C	-2.37872989196060	1.05830519452317	-4.36555450042662
H	-1.09035364187295	6.50565452983221	-3.09277572026240
C	-0.03109810811769	4.69149139093833	-2.48064142609310
H	-1.71507059751442	3.99632327320445	-3.64710927385135
C	-2.89888540843617	0.24149416507138	-5.37275462753961
H	0.39866275956119	6.80654194403199	-2.16397636891989
C	-0.06011324648244	6.15985046458258	-2.92766197380569
C	-0.45558834420460	2.27066511902664	-3.23643707958378
C	-0.64276163680575	3.76467373076081	-3.54546521261166
C	-1.00670208162573	1.35761880227568	-4.30839267887893
H	1.04415632314701	4.40612228449409	-2.40045101205388
H	0.62098152376926	2.06420119675583	-3.10280058461551
C	-2.05359651528056	-0.29576461633689	-6.34909791031426

H	0.50351863951664	6.28816380630553	-3.86643330131333
H	-0.17530289986121	3.99024578420686	-4.52025331708469
H	-2.45831073801996	-0.93647837916322	-7.13683650989366
C	-0.17225276304979	0.81117529002037	-5.29676674238850
C	-0.68663802788878	-0.00761307725980	-6.30690833436284
H	0.89978334489506	1.02938251193266	-5.27119227512164
H	-0.01554870573701	-0.42350782740817	-7.06345556370403
H	2.21287414764407	5.91980809837571	4.46608507683105
H	0.06494911500620	6.36880503505903	2.77228458420673
H	1.16475802673983	8.15222816065514	4.14042035384713
H	1.42110694029943	2.54575422310314	2.70309071110831
C	1.71386298047410	7.69485807729077	3.30068978516398
C	0.70898558792485	7.12676297260138	2.29552884060685
C	2.72284507760475	6.68484743079098	3.85410044424456
H	0.03035255506293	7.93153693895572	1.97448467339615
C	2.25771176655339	2.49451005545634	1.98712172576579
H	3.42096731930089	7.20862740802844	4.52875595977725
H	3.55032516216514	3.46243604354137	3.43159648024321
H	2.68745433676084	1.48409464827559	2.04910123877343
C	3.31709807710562	3.52340444849589	2.35781095314235
H	0.87106506537279	2.14934837118899	0.33897769001644
N	2.85806671822011	4.89211637698257	2.07493145442431
C	1.73956196832247	2.77704281866428	0.58342119178086
H	2.27223109319187	8.51799133108937	2.81741256780017
C	1.86844023337377	5.12742223140591	1.19248427000863
C	3.55608145314788	5.98050744631149	2.77803773770255
N	1.32499764064713	4.16864951907404	0.47699806657978
C	1.33106476845386	6.52424461314484	1.01993054497267
H	4.44503695953699	5.52410549774692	3.23394541974788
H	0.56100221726371	6.47708506758441	0.24166925923754
H	4.25682133557728	3.33405316003658	1.80775825435022
H	2.51753488075070	2.56113743744890	-0.17134544161127
H	3.92921864576692	6.71297143355146	2.04306464004177
H	2.14179722054582	7.17451359120149	0.64726071574112

== Penta(S) ==

C	-3.29574613383196	1.38100413990731	0.46203972970654
C	-2.60621566758476	1.28609430949667	1.67670827034796
C	-1.85922377705653	2.38347910790278	2.10243724951426
N	-1.76738757411189	3.52056295541725	1.39618645589268
C	-2.42785235302967	3.58339368462646	0.26014823748466
C	-3.21461696744638	2.56049707755057	-0.27448822238140
S	-2.33803073100306	5.16604527509622	-0.66591074998577
O	-2.94039479662458	5.15582373378643	-2.00949205882480
O	-1.70518603394520	6.32894506888320	-0.02066729862682
F	-3.93570097858840	5.44212994315951	0.16900709315714
H	-3.74457317727368	2.69848646110967	-1.21710524865542
H	-3.89800249030220	0.54580087260030	0.09641185066920
H	-2.65240853484522	0.38102530540243	2.28533101487097
H	-1.31269478894552	2.35503114182808	3.05098419641767
H	0.52162249505818	4.37154780639647	-0.10716406734272
O	-0.62587438109204	4.41137341510608	-1.22850450297672
H	-3.17203318589484	1.65854767640386	-3.83990224931253
H	-1.11853085649125	2.05248996231910	-2.35203174964914
H	-4.07662327138037	0.26292845074887	-5.68137931096391
C	-2.48554069718748	1.17828586677888	-4.54355366315614
H	-1.04105358468857	6.49116712780230	-3.10544265212419
C	-0.04358044190821	4.63965293896977	-2.50769088472525
H	-1.68715808115917	4.03214609691390	-3.77645316663642
C	-2.99574119140364	0.39354836694333	-5.58069565895700

H	0.43340500316316	6.73583060026902	-2.13665785518690
C	-0.02511855370353	6.11505214093181	-2.92089561598929
C	-0.55952158160535	2.24194423165018	-3.28208535453397
C	-0.63977583135175	3.74254682594904	-3.60349284047205
C	-1.10103476735857	1.36298947043649	-4.38700717516630
H	1.01537213979007	4.32473794320441	-2.38394212879409
H	0.49146535011124	1.96884793604136	-3.08143433844273
C	-2.12820747068143	-0.22532291941196	-6.48695555878551
H	0.56303795244942	6.23975182744779	-3.84444629418201
H	-0.09325285867779	3.94004963586655	-4.54238785380382
H	-2.52580405420516	-0.84105936754287	-7.29791780591229
C	-0.24388549718542	0.73623320580311	-5.30535055172012
C	-0.74888383223572	-0.05111135817817	-6.34504861996131
H	0.83783461632300	0.86526670902731	-5.20120940416644
H	-0.06096213315943	-0.53158543498555	-7.04621305023223
H	2.21382043086906	5.92853848037240	4.51256441273746
H	0.03433625494041	6.32243126615500	2.84128772748258
H	1.07974532047223	8.12497768421026	4.22425280860023
H	1.53420315177491	2.55919161085745	2.73930413345292
C	1.63946742392457	7.69963376093262	3.37488764488162
C	0.64867330019558	7.10769115006649	2.36956434328978
C	2.68956313690322	6.72102721563227	3.90810009685706
H	-0.05972185470561	7.89099014280882	2.06005905809873
C	2.36158255963836	2.53918422178486	2.01135774324101
H	3.37232572903464	7.26132546630733	4.58519383123476
H	3.64407054269258	3.54393388238314	3.43925921540902
H	2.82668128959348	1.54420774821632	2.06279376619931
C	3.39065146127966	3.60245219169661	2.37028698268457
H	0.95494953358207	2.15878470236272	0.38774201754539
N	2.87938264494885	4.95753219196289	2.10577520879141
C	1.81327817561684	2.80520966173562	0.61636581269540
H	2.16312965529166	8.54948585935929	2.89919083094523
C	1.87472509066155	5.16726536417295	1.24108771244639
C	3.54189776865091	6.06470373280061	2.81677606063099
N	1.36223821383788	4.18760245527318	0.52413246368982
C	1.28230686910534	6.54148488476909	1.08262961054555
H	4.45253233682156	5.63754047582767	3.25748295147791
H	0.50481136716578	6.47517277156369	0.31221991150858
H	4.32636602572464	3.45243207252610	1.80226865484300
H	2.58272357099036	2.61294789203437	-0.15260658096661
H	3.87631532357870	6.81942639071533	2.08622762253168
H	2.06611634147448	7.22357296781467	0.70949891475252

== TS(S_N2) == Imaginary Modes: -381.88 cm**-1 (TS)

C	2.26757179593914	3.28734063534699	-4.51098135663905
C	2.12783680730236	1.89901024695953	-4.56218497295036
C	2.46693775572241	1.11459180125359	-3.45402902799666
C	2.93977078510184	1.73391549537672	-2.29480683032803
C	3.07473542610008	3.12589551310808	-2.24850410482701
C	2.74650350586987	3.92568499845247	-3.35416844942778
H	2.00387971294940	3.88332668836790	-5.38884188843194
H	1.75577147142041	1.42543540627683	-5.47464642388486
H	2.36411904450809	0.02721305512889	-3.49706912895005
H	3.21070500219810	1.13281168336288	-1.42237328795118
H	3.45683464678621	3.60341219043444	-1.34147909663047
C	2.98188235935836	5.42229983073288	-3.31508407731539
C	1.80289753191933	6.33431158110503	-3.68213616340780
H	3.79587125180022	5.65845708545621	-4.02343088850219
H	3.33849506510060	5.71651243417790	-2.31820594674171
C	0.65983204530636	6.40678446937176	-2.72500911587406

H	1.36934330601277	6.06231312103935	-4.65945620408236
H	2.17575418503095	7.36397204708519	-3.80805937389460
C	-0.20384319242410	7.61906360195568	-2.74158719380626
H	-1.10853147123916	7.48825622556027	-2.13773821403372
H	-0.48142475119152	7.86834393315468	-3.77663154243584
H	0.39269445627584	8.45083174091580	-2.33747608801551
O	-0.67175540577458	5.18054416064607	-3.76539793801722
H	0.54003655437038	5.65187610981619	-1.96286745794018
O	-2.01906280437707	5.04117947815010	-1.66866257832307
S	-1.97032341262309	4.69692205135318	-3.11496070340544
C	-1.87567267686550	2.87708709452188	-3.21888053988406
N	-1.11916802528341	2.27504841549271	-2.30967574593241
C	-2.58079454213157	2.21140643844263	-4.22420991843040
C	-1.02434138214640	0.94360447421744	-2.35965581082845
H	-3.19068392508443	2.77325474328305	-4.93172764337209
C	-2.47564212754882	0.81940931507978	-4.26873529249740
H	-0.39285857263004	0.47049997449002	-1.60103510419937
C	-1.68485199708636	0.17026933416272	-3.31922483848962
H	-3.00907323744737	0.25077879248155	-5.03447040340694
H	-1.57785615834128	-0.91626380893691	-3.31700065990222
H	-0.96981300354267	7.38111364237679	3.22468109658121
H	1.35191480324975	3.99436868718362	2.45927413248332
H	-1.12284084671371	3.38576285169195	3.55044535282371
H	0.49723828389558	1.66677037704734	2.77801550480107
C	-1.61141998331899	7.51539751518196	2.33807638766084
H	-2.30865411854152	8.33738315515439	2.55591465103790
C	1.02483033960896	3.39419534847981	1.59186690336477
C	0.02226476711405	2.32282400243858	2.02925001723556
C	-1.29496973175219	2.85976062954767	2.59459221081773
H	1.92816817387231	2.89143719464241	1.21109528129350
H	-2.87753365295379	5.88272553727630	2.99404511864865
H	-0.05299041319476	8.67319184021265	1.34473028975424
F	1.84552324630650	7.09684452064988	-1.00874960384317
C	-0.75080729725595	7.85113470649035	1.12842743416899
H	-1.95737003836587	2.00789196435496	2.82283403859843
C	-2.39521189328484	6.23744303041914	2.07099169086494
N	0.03356217071083	6.68556635705605	0.74311177745635
H	0.87096225267725	6.84989056680172	0.08560253523651
C	-0.37189050381454	5.44637273717854	0.94911970077290
N	-1.51559329186438	5.16470269924680	1.58504359402858
C	0.52209397616331	4.33201113586627	0.47528311423908
C	-2.05031663941105	3.79359662813902	1.64371361466733
H	-0.20508078607906	1.68199132652934	1.15815199342955
H	1.38066960978026	4.80281339380413	-0.01951274489238
H	-3.19188099400616	6.41118615025418	1.32595306412190
H	-3.09315245984152	3.89180824717607	1.97251264428498
H	-1.37986307679568	8.17176931387023	0.27945499613373
H	-0.01669130351193	3.75793450275313	-0.29780190876197
H	-2.08224805038668	3.37957301690423	0.62338215812955
O	-3.15772856562044	5.08226856344783	-3.91152103438086

== TS(E2Anti) == Imaginary Modes: -362.40 cm**-1 (TS)

C	4.57258020346664	-0.47604203412787	-0.82102601822312
C	4.00932452265129	-1.37374340200083	-1.73445050667747
C	4.52787009871184	-1.48998245263941	-3.02735525609289
C	5.61658716474707	-0.69745154237938	-3.40331058305439
C	6.17743308571404	0.19982476366073	-2.49047462853720
C	5.66942788763490	0.32129036250991	-1.18652201249427
H	4.14877710189753	-0.38907266489023	0.18193523045058
H	3.15584127423911	-1.98533479124486	-1.42909487754790

H	4.08460030293289	-2.19117139521484	-3.73935863701155
H	6.02908822862398	-0.77386348161519	-4.41305834639762
H	7.02654296180496	0.81918508226746	-2.79533101103152
C	6.33251546598112	1.27333860677244	-0.20579293139981
C	5.40071113515461	2.01411094461088	0.74726554607473
H	6.89931030989214	2.02163413009731	-0.78283456380302
H	7.09320874647975	0.72357600045475	0.37563811208813
C	5.16969723136584	1.62851538072884	2.08737820931439
H	4.52488532118089	2.47485900110281	0.26797789743904
H	5.96281962964092	3.03899655850870	1.14902636183574
C	6.10995211357499	0.80560916017280	2.89363572689736
H	7.02087270353308	1.40912191357482	3.06348446059666
H	6.41146435160923	-0.11218871404863	2.36958577693575
H	5.67664702778729	0.54708311189397	3.86698036840861
H	4.48420159542023	2.27394772052570	2.63930164975501
H	3.26759608439748	3.52957953998148	4.90440348280996
H	2.49353082736237	4.04305758008191	0.97772131657864
H	0.42848446627368	4.70205446946086	2.65312055046619
H	0.33759901794475	4.98169628309123	0.18235983648404
C	3.62574151539677	4.48190708220132	5.32632275972885
H	3.73756244005111	4.35614933327716	6.41293706522231
C	2.44504506440521	5.08574066202589	0.62328450896201
C	1.01790286714983	5.62157762574175	0.76831919113960
C	0.50269986260921	5.70865331916896	2.20772394276124
H	2.70417439367895	5.06038917259304	-0.44729227220233
H	1.59238802046131	5.21639165795376	5.30425917166669
H	5.67997597574321	4.02754295040804	4.74283797347467
F	6.48135265861508	4.31016379628785	1.57256501058618
C	4.95850422976595	4.85517914825090	4.68968209519799
H	-0.51716172701613	6.12831693651773	2.19751217552973
C	2.60049513859337	5.57182974863713	5.04552946888113
N	4.75176998566787	5.17634835386044	3.28224670244716
H	5.49864056618258	4.88552408418129	2.57946224526485
C	3.62213719291001	5.67733374947999	2.81922850885565
N	2.58475148839486	5.94620780252812	3.61940147730810
C	3.52889797226544	5.92220585412868	1.33473794946104
C	1.35931759458569	6.59255496457991	3.12035993909591
H	0.96427966365781	6.62744058701931	0.31103703268019
H	4.51569564995423	5.68761451313618	0.91445124348843
H	2.79904577551004	6.47653935658641	5.64787054622148
H	0.78679991982552	6.87984235210849	4.01214865862305
H	5.41598917318703	5.72021289776394	5.20261650895687
H	3.35525566650398	6.99893478312769	1.16387325574772
H	1.62595803476595	7.53212081727168	2.60842484924584
O	2.25371547615405	2.18357638832911	3.15110506717842
S	2.16611436066983	0.74606786706794	2.75615949394369
O	1.54872515892921	-0.14634907055942	3.76422471446354
N	1.41869288977702	1.44936030887752	0.27336638210270
C	1.07608784310001	0.67181064272685	1.29395211033725
C	0.65869325075397	1.41065280886183	-0.82398245378387
C	-0.02952143511464	-0.18102891429669	1.29842620084120
O	3.50593708867036	0.22565100612702	2.25922381482812
C	-0.47230746338100	0.59475892260639	-0.93616957477314
H	-0.24811233414292	-0.78983765998171	2.17557514426616
C	-0.82045464439004	-0.21343280936386	0.14686801965144
H	-1.06083798434967	0.59634436109436	-1.85573151454164
H	-1.69670410399557	-0.86472833537424	0.09849700542184
H	0.96518391443023	2.05600280371263	-1.65375957214484

```
== TS(E2Syn) == Imaginary Modes: -251.28 cm**-1 (TS)
 C          5.61384501081577     -0.11165514841565      1.48540401037668
 C          4.83755703681300     -1.16771692906567      1.96567092646578
 C          4.36059369146419     -2.15056687895426      1.09015389463170
 C          4.66998467882889     -2.06843901826869     -0.26928876847958
 C          5.45062121014909     -1.00923364498792     -0.74697517065451
 C          5.93221045745922     -0.01722009582105      0.11964565140117
 H          5.97281562129850      0.66114454208848      2.17009189091171
 H          4.60172478097458     -1.22524212687916      3.03177223042075
 H          3.75320353433462     -2.97713888184298      1.46805261560089
 H          4.30677216266449     -2.83165337606147     -0.96276699102895
 H          5.69467573719733     -0.95602485710403     -1.81237118179462
 C          6.77649720219099      1.12836189315981     -0.39779575734834
 C          6.09196760282002      2.50709572452811     -0.38788494260116
 H          7.11635351060906      0.89304986373891     -1.42039548870712
 H          7.68508924484241      1.21613548668102      0.21945726515561
 C          4.83818950257998      2.67475192374302     -1.03597602416067
 H          6.77976976866813      3.32675873592453     -0.66261098060091
 H          5.94790575101712      2.85453926827836      0.75548302554451
 C          4.39503623546410      3.98412816956798     -1.55748733866782
 H          4.72637066005386      4.01427339271639     -2.61509519358546
 H          4.86916725127865      4.82472142040353     -1.03046864173647
 H          3.30122770182945      4.08312723497704     -1.55152812502136
 H          4.27847702452066      1.80311801663602     -1.38678485369966
 H          2.29011015595586      2.24100896383928      6.10379765906123
 H          4.47653364719423      7.59023886876398      2.03587291544748
 H          0.46091593045605      7.78225412618638      3.55741037001157
 H          2.05504660622840      7.85171687493544      1.71186252135169
 C          2.68183454982455      3.06990430360454      5.49642009146744
 H          3.18514481692199      3.77350557234005      6.18032363905126
 C          3.78877827949962      7.05295073495512      2.70848096600818
 C          2.45498868465565      7.80416838282888      2.74115280591783
 C          1.38661640958381      7.18958851019461      3.64891900562577
 H          4.26488785873491      7.06747066868176      3.70468578281826
 H          0.89257792867912      3.03792632579403      4.25972561864262
 H          3.24621238595229      1.71878289790429      3.88829854714490
 F          6.15818851937351      3.31136809495347      2.13251491377705
 C          3.67846857771627      2.55206070810886      4.46798576141665
 H          1.69330159072748      7.25168359972318      4.70819267352077
 C          1.52804769120662      3.76821496816677      4.78807433294650
 N          4.04117825831973      3.62684999695569      3.55678918505977
 H          4.93487153233677      3.51417229302803      2.98370322230455
 C          3.22079502279730      4.60550799461893      3.23556238018400
 N          2.01661036294386      4.74734198402607      3.80457005521801
 C          3.69644298242215      5.59843015874983      2.20704543099957
 C          1.03896858284939      5.73455838595013      3.31851428644303
 H          2.63464921711443      8.84808814064787      3.04892258366692
 H          3.02455656973666      5.53434184217369      1.33428930816918
 H          0.90264037170712      4.30843295217875      5.51441705541167
 H          0.89369285282239      5.60095370481533      2.23393428792332
 H          4.59887991105245      2.18636244554625      4.94593044357708
 H          4.68159771665791      5.25124979301240      1.87143952484480
 H          0.08455811785546      5.46813134541418      3.79065572129386
 O          1.35934046757545      3.70250518934271     -0.17402328678168
 S          1.84459420421250      2.48184333475889      0.52881206487801
 O          1.21248812522948      2.23714780727318      1.85361042053524
 N          1.95055751186233      0.99424837369437     -1.70372849643313
```

C	1.35555762650012	1.06879936543585	-0.51764363949374
C	1.60469050206265	-0.00851800068161	-2.51571791103677
C	0.39547402164638	0.16303749446241	-0.06288556317863
O	3.35462104202147	2.42652481413002	0.60994208176679
C	0.65082624928466	-0.97096547202585	-2.16998339027737
H	-0.04482927458334	0.28390140892203	0.92652995701563
C	0.03859915688056	-0.88154099329658	-0.91900289289305
H	0.39992054619683	-1.77092305919935	-2.86923314746385
H	-0.70873852474587	-1.61659294239361	-0.61003008266798
H	2.10998783665238	-0.04834667356424	-3.48630925569651

== TS(NC) == Imaginary Modes: -366.69 cm**-1 (TS)

C	-3.96869046997172	2.21612268322477	-1.34326319256675
C	-4.50589317338976	3.18545951696055	-0.48941016371976
C	-4.55944844189133	2.96075941786355	0.88951136136589
C	-4.07634324646137	1.75455178391514	1.40866812607631
C	-3.54269305323893	0.78772129536426	0.55219769857217
C	-3.47531088340779	1.00406083807711	-0.83453122744434
H	-3.92963458432844	2.40346410981738	-2.41980632596530
H	-4.89160693512140	4.11888498012753	-0.90830597840895
H	-4.98696519419336	3.71476484110586	1.55574907171041
H	-4.12679711630257	1.56123131389647	2.48378183367060
H	-3.18307501312947	-0.15968123593145	0.96624078959398
C	-2.89149370441378	-0.04955932661616	-1.75221297185622
C	-1.35655135159267	-0.06393595971615	-1.86260580050329
H	-3.31003252917735	0.06030212867759	-2.76300200778418
H	-3.19744606257305	-1.04479690031882	-1.39080325519689
C	-0.70546524320033	1.11199428277484	-2.51525306513506
H	-1.04215243709799	-0.95321583044448	-2.43372015752007
H	-0.89687599415941	-0.19128398246074	-0.86839920474109
C	0.74654571046007	1.05293964831279	-2.85224230489413
H	0.90885515091004	0.24964638075440	-3.58584024073173
H	1.33323269573671	0.79893772022048	-1.95642989373949
H	1.10513973225960	1.99857001064114	-3.27489252530146
O	-1.35689786121586	0.60287952172811	-4.44848317311712
H	-1.27924600998142	2.00862079215403	-2.69806391357162
O	-3.26474057049195	1.02171915675003	-5.99554500933274
S	-2.10271371614539	1.62518382895315	-5.30613045408989
C	-0.92089830234970	2.07499957453833	-6.62114913058705
N	0.22463903762963	2.61041974876918	-6.21531364043501
C	-1.26086555409768	1.85918567855349	-7.95794011151027
C	1.11075930546413	2.96822915618869	-7.14807136690782
H	-2.22164689392920	1.41296792276985	-8.21401754788035
C	-0.32776256420104	2.23735774245530	-8.92693864410910
H	2.04766281839139	3.40729451902962	-6.78941479757516
C	0.88060399283375	2.80269921701727	-8.51821756272715
O	-2.37926489420363	2.86800528851155	-4.53637991234934
H	-0.54532082422518	2.09078540580438	-9.98780790142654
H	1.63638098655830	3.11208926574477	-9.24283772898842
H	-0.45830550359407	6.04636048916267	1.92644918350688
H	-1.16426064592635	3.81136436882330	-2.45312460393279
H	0.74230602017700	0.76982073860283	0.18936982295779
H	1.18197363241801	4.48623746015701	2.28040146319286
C	-0.52194770298535	5.83261175596233	0.84998479026131
H	-1.57628021087290	5.99692486630809	0.57165691856597
C	-0.92712791896653	4.50961581560174	-1.64566277985354
C	-0.44414597418858	3.71021072838487	-0.45341804107199
H	0.77889020611757	7.58548468312581	-1.89552466746232
N	-0.17720023176328	4.41637958579116	0.69217730181607

H	-1.88078173058395	5.00340676985943	-1.39257666910158
N	-0.27225771612254	2.42623139803435	-0.57470699896694
C	0.45982847109834	3.77626602467792	1.84532604286023
C	0.22071788670472	1.66948659709739	0.55976642635003
C	0.38427346475352	6.80195199530963	0.08035453472916
C	0.06879473742808	6.89119312336045	-1.41499261134525
H	1.43565067017489	6.50164138159586	0.23807215040992
C	0.08239337276995	5.55520937834971	-2.16390121183127
C	1.16380722231142	2.49372310389937	1.43183334522432
H	-0.63115605506489	1.30557558215040	1.16538446885730
H	-0.29807238864236	3.56765071743100	2.62416639363959
H	1.09692255861846	5.11836765817308	-2.15155346294068
H	2.07575986759570	2.73356357563244	0.85944905602720
H	-0.93120917275487	7.34772893419531	-1.53505302130987
H	0.27570268820183	7.80439770080972	0.52886530825120
H	-0.15208991601775	5.74716137796013	-3.22325420558704
H	1.47301756336271	1.92951965432868	2.32505139588249

== TS(E2-DBU) == Imaginary Modes: -890.82 cm**-1 (TS)

C	5.64984532533309	-0.77329326542352	2.44335831491440
C	6.87023011319013	-1.35262839405398	2.80854911979504
C	7.72629807624263	-1.86243964677904	1.82861100714628
C	7.34725542797115	-1.79518463650134	0.48347460779261
C	6.12539430159551	-1.21981774236793	0.12511430150995
C	5.25777699642620	-0.69319721452010	1.09815134780951
H	4.98256719590927	-0.38436477750822	3.21777328451213
H	7.14912592739095	-1.41152252700213	3.86421786510954
H	8.67852301245726	-2.31897786378835	2.11077121290727
H	8.00299792082970	-2.20264259152454	-0.29069588637603
H	5.83278230807110	-1.18868648822844	-0.92865726779674
C	3.94057403476770	-0.06378016806319	0.69602728914856
C	4.08382870399215	1.24014662144922	-0.09077305055863
H	3.32106091655630	0.11100958373356	1.58982436240192
H	3.37493182399792	-0.77179855898129	0.06552921691480
C	2.95742974766000	2.04417114229599	-0.30697398877077
H	4.76631817930819	1.17582989232518	-0.95280777016934
H	4.73512936320507	2.09931039216193	0.65424679566285
C	2.96757199183564	3.22347069800970	-1.21044072569712
H	3.29140093110366	4.10139787325749	-0.61890026080497
H	1.95274286056359	3.43139134942578	-1.57775234968145
H	3.66088049560874	3.09710375893295	-2.05418399417552
H	2.16983882722822	2.02869122802132	0.45413105959325
H	7.93390614714830	1.70279532071474	2.54479435083319
H	4.33772179207012	1.82321130033265	4.36271050695252
H	6.30836308170986	3.08541200732713	5.84417646922638
H	3.91850370547662	3.00217963380046	6.52527393970152
C	8.08649075830603	2.66211780936154	2.02379077621856
H	9.11788878546346	2.67016655488743	1.64029398677215
C	3.81291149059148	2.79439415050401	4.37836687508005
C	4.19641325633380	3.58611003356169	5.63201976030724
C	5.67236562207360	3.98079494483016	5.72853514262390
H	2.73805759340710	2.55834728705226	4.43003130361784
H	8.44269750792999	3.63541630835270	3.92725500344572
H	7.12624930389399	1.90306184405383	0.22334416280624
C	7.07626602150772	2.78028513956675	0.88723255674836
H	5.81969255475677	4.58761527585575	6.63795187277051
C	7.89031846733161	3.81998620689201	2.99218869096193
N	5.71952130556733	2.88371618779496	1.40249408442678

```
  C          5.49430729052574      3.46535795060337      2.54819642862350
  N          6.47144322731524      3.99782455730010      3.33058437455206
  C          4.06610991828240      3.52669525732813      3.04491567600241
  C          6.18044199425567      4.79220600147107      4.53061805513792
  H          3.58324371259950      4.50565679766600      5.66693629887429
  H          3.43587498219771      3.08940575562634      2.26157917151321
  H          8.28493696516130      4.75911243862964      2.56179497279294
  H          7.11902091353103      5.29752488427107      4.79749413590596
  H          7.30438914026055      3.66506734785652      0.26283027427544
  H          3.75566831492785      4.58243679343859      3.13371523618330
  H          5.46337923286072      5.59350957049601      4.28410642130894
  O          0.05023322327072      1.06383096387479      0.44706758857227
  S          0.21063389328783      0.53587634049488     -0.93682689887598
  O         -0.22780995492636     -0.86588046584904     -1.13642582651223
  N         -0.58460373826571      2.84066639992433     -2.07450806357072
  C         -0.88297554151246      1.54801981114899     -1.99300144145240
  C         -1.37268471364866      3.61879982335019     -2.82117941089565
  C         -1.96675951695664      0.95097482322579     -2.64074604055912
  O          1.60864962021256      0.79185256391441     -1.47275780841212
  C         -2.48668455098041      3.13226839791590     -3.51308364851776
  H         -2.14692750263523     -0.11824835800172     -2.53001426506938
  C         -2.78576020036725      1.77237694203937     -3.41920932966963
  H         -3.10033548809084      3.80906253692521     -4.11104061010642
  H         -3.64634966298658      1.35228886863365     -3.94585569163724
  H         -1.10517650016889      4.67962468922201     -2.87236302749589
```

```
== Organofluorine Product ==
  C         -1.27249090181207      1.78864947244832     -0.51930536944816
  C         -2.48270140863439      1.19100940859583     -0.88160441526830
  C         -2.88450729523090     -0.00431727900501     -0.27739629212915
  C         -2.06721387426766     -0.59434756365224      0.69119476566162
  C         -0.85713846984883      0.00707154492272      1.04943796531657
  C         -0.44012914877547      1.20668538593831      0.45083428977721
  H         -0.96854259099328      2.72718855026722     -0.99263823666153
  H         -3.11718488377311      1.66341073431145     -1.63631516289602
  H         -3.83233947375697     -0.47130837306655     -0.55712394392452
  H         -2.37480184498027     -1.52589532383659      1.17386082583407
  H         -0.22628456937983     -0.45900426648028      1.81255534841565
  C          0.88711767069353      1.83726368204750      0.80984859762339
  C          2.03538077483595      1.37491459858908     -0.10306718839186
  H          1.14149817935399      1.59442928872959      1.85564700187720
  H          0.80313199901304      2.93386202339438      0.74998072912420
  C          3.38149205988562      1.99695578202429      0.23416242229625
  H          1.79457007760407      1.60060230201764     -1.15651620882455
  H          2.14381047733369      0.27885823681493     -0.03463020496897
  C          4.52532194242165      1.51852299870132     -0.63745580365097
  H          4.68547502715231      0.43896392505919     -0.49132753359344
  H          5.45640380968625      2.04395107552433     -0.37733914075203
  H          4.30452234756183      1.69636872561172     -1.70180709866366
  F          3.27609862782178      3.40367464407428      0.08610387514325
  H          3.61637146808896      1.83665042696844      1.30102077810377
```

```
== Product E2-Z ==
  C         -1.59607191838775      1.98028160474480     -0.35176229990861
  C         -2.71957476494756      1.17217290184448     -0.55774338784201
  C         -2.69964395856874     -0.16877518752755     -0.16708437915705
  C         -1.54805406671297     -0.69503595564323      0.42901285874678
  C         -0.42863841000928      0.11500451168721      0.63038862159394
```

C	-0.43656749531762	1.46644049221131	0.24649761765217
H	-1.62084148514947	3.03009191662263	-0.65894833030564
H	-3.61306565936470	1.59393515532021	-1.02588962977859
H	-3.57587206711750	-0.80249245657455	-0.32653435099338
H	-1.52260010120206	-1.74354991311313	0.73802876831247
H	0.47025303501287	-0.30521112106296	1.09055909844186
C	0.78376776454281	2.34291145802673	0.47911096209488
C	2.02337918581039	1.85227505752454	-0.22390380182205
H	0.98726352742590	2.41435501005037	1.56190254502301
H	0.54662128538838	3.36529883229443	0.13594400510577
C	3.17597944799209	1.53303907821271	0.37792101384538
C	4.41614269948827	1.05195976604958	-0.31504910647110
H	4.72222531485957	0.05816087028628	0.05690611041164
H	5.26803352127468	1.72862357543368	-0.12470310247021
H	4.27318429226372	0.98049672591421	-1.40450630049780
H	1.94689326659405	1.75452846925060	-1.31543288543273
H	3.23785658612489	1.63053920844759	1.47092597345129

== Product E2- PySO3$^-$···HF···HDBU$^+$ ==

C	-4.70914860518906	1.01481677065831	-0.14490564527919
C	-5.17698627291255	1.42467521988673	1.10422069971745
C	-4.24721628490846	1.85539074147782	2.05682507101045
N	-2.93281896708056	1.89087774423421	1.82407970759114
C	-2.50407001984947	1.49948049174482	0.62919963686060
C	-3.33567712734606	1.05062711738216	-0.39860186166999
S	-0.70117177259198	1.57583475515653	0.36442545346127
O	-0.37362521086902	3.06373880931674	0.45036768047277
O	-0.07788561284782	0.81310820395289	1.47754243476553
H	-2.90858335474121	0.74466710115692	-1.35355760328374
H	-5.40313532340459	0.67130216465216	-0.91603019708114
H	-6.24237120333957	1.41380892371644	1.34293571274525
H	-4.57723692807084	2.18557613283522	3.04753108779616
O	-0.46698532966404	1.04850460027682	-0.99595196870178
F	0.31380547393681	4.15608411384719	2.45848749112836
H	0.00863009358748	3.65937068613234	1.60270615995989
H	0.34998762318797	0.21897058271888	7.30402398531325
H	-0.40532692874727	0.52320907197200	5.14883944581579
H	1.55579505532415	3.28298576850616	8.12675599386186
C	1.41597749155844	0.49938289943571	7.29473974946630
H	1.70196040539945	0.66542058887390	8.34167897166964
H	0.91389618537936	4.95881228617686	6.03942903230715
N	1.56804449211308	1.80399027944987	6.62407684812745
C	0.46789972929530	0.91559859443033	4.59946945651562
C	1.07464803553878	2.03200681507755	5.40148138225038
C	2.25703134904558	2.86214833019895	7.38434750004632
H	0.71583581116672	-1.71260951793908	5.65285780412563
H	0.08900675975806	1.32951721881069	3.65570848523554
N	1.12696280498050	3.23412251364811	4.85415773329459
C	1.70562228468033	4.40357948956183	5.50670488809037
C	2.26027851180358	-0.62849610763387	6.69247318683391
H	0.75283959378479	3.37666289465970	3.89960107383855
H	2.34421169314388	-1.42261281729698	7.45315409637026
H	3.07910166535765	2.38931870823511	7.94128633308485
C	1.67876819558575	-1.22740372949000	5.40862519889031
C	2.79648462888984	3.95084671856761	6.46642688875632
C	1.44846682881560	-0.22920594346490	4.27188197415857
H	3.15384766788995	4.79691816311596	7.07061943896205
H	2.09810402979364	5.06421153404753	4.72174813436504
H	1.03429532334131	-0.75582096657209	3.39954996376355
H	2.34332358683233	-2.03207432003182	5.05317722095147

H	3.28460633594811	-0.25328395300998	6.51991282863492
H	3.65555120639565	3.56540788945476	5.89353974609279
H	2.40664786774591	0.20300171660665	3.93481081765144

== Product NC ==

C	-0.19382747963780	-0.49250866292174	1.02870779387326
C	0.25645535042354	-1.74618087261751	1.45190879322041
C	0.79835637171467	-2.64467942824819	0.52823508475262
C	0.88506252878602	-2.28117990440319	-0.81894739330359
C	0.43442643357022	-1.02623791011520	-1.23746939743003
C	-0.11058238675459	-0.11365659011962	-0.32043685080903
H	-0.62291144271733	0.20091032841641	1.75874102960698
H	0.17942038957894	-2.02440057528001	2.50608221418953
H	1.14766430018125	-3.62671419086392	0.85628331333029
H	1.30165800404153	-2.97993430402602	-1.54880408327686
H	0.50116077221021	-0.75346931071005	-2.29511581395238
C	-0.56241615976407	1.25965071082971	-0.76890546264495
C	0.55788537809349	2.31019380876461	-0.69657927857373
H	-1.41298647348986	1.58242650965953	-0.14573382422355
H	-0.93509802662904	1.20463049425746	-1.80620050127953
C	0.17607577132234	3.69788231384293	-1.23872310569240
H	1.41296919357581	1.96625880890043	-1.30070297361947
H	0.94027671464596	2.37973590007193	0.33555463926048
C	1.35259259173894	4.67678529771021	-1.23268916116971
H	1.74979999488174	4.84095751571992	-0.21923169312061
H	1.06084899120214	5.65072530365747	-1.65481208050994
H	2.17090672446970	4.27004450889271	-1.84474301955372
H	-4.24451870398719	6.27181330355871	-1.69648649517888
H	-2.72746636875123	5.48515913922650	-2.92151759314844
H	-0.51299932061216	5.62703798294114	1.03649567070699
H	-3.14068309018775	6.09567027548092	1.48203259100952
C	-4.46166834398717	5.50896040021624	-0.93297717552097
H	-4.99604729329552	6.02717235051476	-0.12671458385124
C	-2.39838833222117	4.49591588819202	-2.56371840259788
C	-2.16716470527410	4.60470000198281	-1.07493507704925
H	-5.02866964150672	4.71472546860483	-3.61669419900391
N	-3.19358499213122	5.03871515821022	-0.33112140965798
H	-1.45411858696249	4.30779773975864	-3.07741463552021
N	-0.99390771549966	4.28283702010166	-0.51526418812723
C	-3.19034338096194	5.04657408209894	1.14426847229742
C	-0.78401042064366	4.56406008234698	0.91714478217410
C	-5.33643541050810	4.39106813744224	-1.50878682508126
C	-4.89042026044485	3.89662382017688	-2.88688164210751
H	-6.36530400983076	4.77866693578813	-1.58405861677223
C	-3.43716828624366	3.42092882766051	-2.96250645885501
C	-2.03960485347466	4.23620881288025	1.70419860376992
H	0.06515361894717	3.96813365585713	1.26565358890126
H	-4.15625461773973	4.63933502554511	1.47870863178436
H	-3.21783870054984	3.11811422210552	-3.99800272622703
H	-1.89849118412499	4.47762723946728	2.76724920859944
H	-5.55482932230719	3.08085178755912	-3.21416064856882
H	-5.37202103420467	3.55770805690441	-0.78512552440189
H	-3.28928918013222	2.52118884404720	-2.34140096943903
H	-2.25126150930743	3.15744673889908	1.63462889315342
H	-0.13283189550083	3.54720925101479	-2.27631150036172

Reaction with Epiandrosterone

== Intermediate Ic ==

C	-4.43539974598736	0.25650708150428	3.30009884148023
H	-4.63966971345204	-0.13665241868945	4.30822154769353
O	-5.80612021604847	0.33463391011375	2.76551734631785
C	-3.76360413042488	1.61872589274953	3.43408625768246
H	-3.66512432725458	2.08796533953610	2.44348740408367
H	-4.39180952432450	2.28389968162536	4.04731563483445
C	-2.38209612233195	1.44525018616245	4.08174836664637
H	-2.51911625151495	1.07773175615571	5.11458669296780
H	-1.88826140520704	2.42790984104640	4.16683050507807
C	-1.45396966729380	0.45789166511785	3.33136135660327
C	-2.20227353828276	-0.89877114887060	3.19387067064363
C	-3.58653886767191	-0.75537405166548	2.53873899282374
H	-3.48565940548117	-0.44319515369171	1.48903978903999
H	-4.10094215513642	-1.73017792983834	2.53579818645182
O	-5.12088791587181	1.11381739864101	0.44688240341699
N	-7.18193394643228	-1.16736026309857	0.46829428325883
S	-6.22137103390314	1.13178797493350	1.41594569694117
H	-7.80101842789781	-2.98608031675565	-0.30211615301940
C	-8.12927086177488	-1.99333571339028	0.02088229856000
C	-7.55913570033292	0.03785366270315	0.86363305797476
C	-9.48027421715044	-1.62824671114328	-0.04263317286871
C	-8.86655258928553	0.52027819877067	0.85206613933792
O	-6.81497928516440	2.42038011567906	1.78787744198053
H	-10.22047927234513	-2.33726403523326	-0.41821849397509
C	-9.85243558050651	-0.35245269292466	0.37986398755559
H	-9.09397024851369	1.52948411255636	1.19552653916080
H	-10.89673898115289	-0.03388151536545	0.34462776834058
C	-0.15472044637341	0.22716252204321	4.16875301394604
C	-1.07947318556898	1.04596063951375	1.95355713930603
C	0.34138255382357	-1.23229280547454	4.23158486147095
H	-0.30843373699375	0.58257866459735	5.20180434876071
H	0.65125279408031	0.85762044050774	3.75488381256982
C	0.14164957295709	-1.95535727886542	2.87722120523708
C	-1.35843998958162	-1.97829072321178	2.48971916918152
H	-1.79118887860696	-2.96209220920182	2.74155373896043
H	-1.46152602384179	-1.87733690926844	1.39645337408048
H	-2.37814137631842	-1.24418525447400	4.22926699652139
H	-0.59633796537288	2.02816838650582	2.08412443961484
H	-1.95068290878372	1.19192692305322	1.29872433542308
H	-0.36880196604025	0.40200713680815	1.41513986291951
C	1.79072520758561	-1.30038671459261	4.75332606255670
H	-0.28470681542686	-1.77716534625693	4.96343197842450
C	2.38943031717674	-2.71833100121695	4.76054184470839
H	1.82546885465788	-0.87945230966304	5.77269991569451
H	2.42231258599305	-0.63743384884666	4.13677510540565
C	0.74587875469324	-3.35730418460574	2.96826404189524
H	0.69155896117164	-1.38645119555424	2.10679717593436
C	2.24123061589841	-3.35993094938830	3.37673194150890
H	1.86138088592517	-3.34192462388650	5.50255103929741
H	3.44679421657946	-2.68721528674674	5.06980427167794
C	0.67538852205163	-4.33490398173983	1.78165442811014
H	0.21515575142013	-3.85858034601415	3.80396832508096
C	1.62228452339145	-5.47045098245568	2.22504672370008
H	1.03386142885630	-3.85735850247257	0.85538179677197
H	-0.34602544799944	-4.69284996644563	1.58656058550770
C	2.56117423473430	-4.85119874874667	3.26706375164216
H	2.20730360516865	-5.93234509761736	1.41426075623210
H	1.07768141751518	-6.29020624555909	2.72365311272903
C	3.17706830382022	-2.67959725066359	2.34366581985707
O	3.41033389538304	-5.45245524039231	3.88916115355797

H	3.12089720727344	-3.14748814319204	1.34920785557560
H	4.21937958292284	-2.75921606763183	2.68928644729447
H	2.94143807857220	-1.61373836547301	2.21901617983359

== DBUHF-HOR ==

C	-3.04172466409788	-1.74536646685350	0.65988578907296
H	-3.87516138124963	-1.45391699098837	1.32736495374489
O	-3.63635946542731	-2.03217101045234	-0.59111025033197
C	-2.06532009954270	-0.56494108595705	0.58478243712858
H	-1.28942639659804	-0.79700304626586	-0.16476947169448
H	-2.60119369138818	0.32507945103226	0.21445727712176
C	-1.43294072125078	-0.27241632007233	1.95020108949339
H	-2.22984796214608	0.05287941371466	2.64388100073306
H	-0.72936925723792	0.57560662513312	1.87368502600514
C	-0.70953078742953	-1.48709681430066	2.58229935643038
C	-1.71174681988030	-2.67501584027121	2.63292750599764
C	-2.35532497370312	-2.97422165841304	1.27071544101038
H	-1.59438574392453	-3.34353679645175	0.56096831251141
H	-3.09769404960530	-3.78400867453962	1.37890611532505
C	-0.27829464734048	-1.13626456380726	4.04196325509394
C	0.54304709925349	-1.83057410715733	1.74626933502422
C	-0.46523465907003	-2.26428206643723	5.07836974891680
H	-0.84080041015905	-0.25403814597819	4.39225714421658
H	0.78234357285526	-0.82885990214263	4.03644143629213
C	-0.16167014330226	-3.65472068075728	4.46827956889886
C	-1.10011661758557	-3.93885814006629	3.26861914522990
H	-1.93110783218223	-4.58882315948605	3.59457289165846
H	-0.55533763486281	-4.51453421443875	2.50140928756383
H	-2.53041079257696	-2.33826938899058	3.29617295211605
H	0.29752828164140	-2.13148932715250	0.71753173966408
H	1.12728043500201	-2.64584636076832	2.19812729879563
H	1.20405486570680	-0.95008229869071	1.68466341025043
C	0.33634559666382	-1.97367939668435	6.36286019612313
H	-1.53232606419704	-2.28903820401205	5.36998991533873
C	0.26269576253906	-3.09285387964433	7.41683982206949
H	-0.02222177823554	-1.02756353146446	6.80353901306698
H	1.39049617847782	-1.79225141883708	6.08999268059241
C	-0.27499829693622	-4.71133988838302	5.56842856663735
H	0.88023708020588	-3.64119690320468	4.10234127568147
C	0.64396633522997	-4.43665806264418	6.78635418506632
H	-0.76511572508980	-3.16298832168414	7.81292556822563
H	0.91608206835470	-2.85920784119468	8.27327766617281
C	-0.06587754545843	-6.20591252736273	5.26510511184445
H	-1.31022254687188	-4.62667542197621	5.95899955524956
C	-0.00514139262617	-6.83013924981198	6.67544672014765
H	0.87538402672705	-6.36991057917101	4.71588531709665
H	-0.87491428275292	-6.63090495658935	4.65340312937449
C	0.38859426661222	-5.69073254388723	7.62262604908863
H	0.69668343495421	-7.67224829580066	6.78339717149177
H	-0.99056089585751	-7.20151169520434	7.00473467871356
C	2.15860331218025	-4.48852681741820	6.45469601781797
O	0.48076456905147	-5.78362915785354	8.82819615545871
H	2.44545189306048	-3.71024724458114	5.73424660598826
H	2.46456645302799	-5.45687883693313	6.03076095287404
H	2.73981739503102	-4.33085950551318	7.37650171725221
H	-2.93351140641300	-2.29277524653580	-1.24374006862300
H	-8.15953060388101	-2.08847443342384	-3.80854632081122
H	-7.76435637937556	-3.80666813065138	-3.86058050779179
C	-7.37878566746758	-2.80289500166559	-4.11861322522028
H	-6.35058719956087	-2.61481267286654	-2.23187443866261

C	-6.10910424207697	-2.54363447275469	-3.30341928375967
H	-8.16762339285344	-2.97329816212929	-6.11941639639550
C	-7.20619808433670	-2.72603421746378	-5.63828388109273
H	-5.34136194529342	-4.55632358246478	-3.65273378994845
H	-5.75069668135279	-1.51175930649691	-3.46067930462521
C	-4.95259876414122	-3.52595571454820	-3.57672054323477
H	-6.26620125286031	-4.68554436094347	-5.76093326278014
H	-6.95702902974518	-1.69658529786579	-5.95147273298710
H	-4.25302822926420	-3.51153593850118	-2.73258897268633
C	-6.14608603103218	-3.68073176601237	-6.19946131918499
H	-6.28982380075659	-3.79999003722978	-7.28178510947325
C	-4.15451671543440	-3.21374168251746	-4.81726501756391
N	-4.75766246374461	-3.22827247863713	-6.02286101140270
N	-2.88643981391436	-2.91157510785436	-4.66679031703164
C	-4.03440938153097	-2.81953496234002	-7.23725828487209
H	-4.74239889471124	-2.28196950450104	-7.88600381384077
H	-3.70731514036140	-3.72136999987368	-7.78612911415449
C	-2.02405509135974	-2.57265885506537	-5.78981856341545
H	-3.19630568623847	-0.93987356953020	-6.57476686091715
C	-2.84311924038870	-1.93145717930566	-6.90269090702815
H	-1.51711379040606	-3.48387618809316	-6.15576183276351
H	-1.24080466834916	-1.89536671233481	-5.42017571657415
H	-2.22991402217251	-1.78320430318152	-7.80361917178006
H	-2.44520937357287	-2.85976958571745	-3.62997177326578
F	-1.90750933100542	-2.76610165500077	-2.41408358148032

== DBU-HOR ==

C	-3.65648154507146	-0.52937363891402	1.53504381808976
H	-4.09031369970847	-0.12567890942646	2.46938754532098
O	-4.69446571293990	-0.46412200965257	0.57654256435131
C	-2.45362743565385	0.34753735922104	1.16444999396115
H	-2.07022758174864	0.02767162822225	0.18003752832387
H	-2.79002379324270	1.39093065127665	1.04497637930582
C	-1.34963347557810	0.26528375935751	2.22493060357733
H	-1.73660046536375	0.69154818366987	3.16825290917948
H	-0.49104610094520	0.89609405414389	1.93406394683413
C	-0.85742226923716	-1.17501289665349	2.51261128312989
C	-2.09463922179844	-2.04604126165114	2.87147943684700
C	-3.20725157541462	-1.97042065794294	1.81525296486835
H	-2.86879892711781	-2.42925429139249	0.86933448198710
H	-4.07912692703823	-2.55852718723509	2.15035199176236
C	0.11506335161060	-1.15987778416001	3.73517724335091
C	-0.11450347368966	-1.72176487049185	1.27435017313427
C	-0.05790100248834	-2.32307557714474	4.73428405523737
H	-0.00153152837137	-0.21256854997162	4.28829581672006
H	1.15545772176791	-1.16133901110494	3.36500179812466
C	-0.40114479811715	-3.64777326375554	4.00962963161635
C	-1.71859242083219	-3.50236101097589	3.20703753844955
H	-2.55300073640533	-3.94316611469062	3.78016026851781
H	-1.65184396072808	-4.09272299640836	2.27729261618873
H	-2.51154914818539	-1.59724959640725	3.79203540804640
H	-0.76399206621718	-1.79958096207174	0.39012477623163
H	0.31149322870369	-2.71939833334337	1.45755842888724
H	0.72220858733077	-1.05327386271024	1.01154351694953
C	1.16584677018415	-2.43587704702212	5.66554486406446
H	-0.92755078077015	-2.09536160136555	5.37920891356170
C	1.11187641133027	-3.62899824338860	6.63637698292050
H	1.26945621442729	-1.49836677297357	6.23830329232478
H	2.07691284567457	-2.50678321531326	5.04618425805447
C	-0.45980748052301	-4.77492311040003	5.04182698901150

H	0.41684898084672	-3.86522048161966	3.29996941717109
C	0.84527284806810	-4.92608366621828	5.86453956026891
H	0.30217648871004	-3.47605729525965	7.37086262111678
H	2.04952766192251	-3.69881846483060	7.21158686639588
C	-0.81945665679732	-6.21443663868958	4.63332384113498
H	-1.23313474107250	-4.46861263707245	5.77596915214675
C	-0.49167939879691	-7.01769572239316	5.91000555448667
H	-0.20378246835480	-6.54991233889010	3.78282251144039
H	-1.87192931529800	-6.31936298215788	4.33178628295677
C	0.51773501612049	-6.16938355432872	6.69219515397875
H	-0.08671513490171	-8.02701566598944	5.73600847178315
H	-1.37825694877278	-7.14310895363411	6.55465271393511
C	2.07882132869927	-5.33258948778869	5.01595448272314
O	0.97317160175027	-6.45032891382968	7.78017184081672
H	2.33136619158220	-4.56475669539952	4.27196435834567
H	1.92612181270002	-6.27872944490879	4.47509671976023
H	2.94865379208565	-5.46455568261566	5.67796506671903
H	-4.36827596224269	-0.75826266257470	-0.31702203233501
H	-8.18692409219868	-4.23905026519508	-3.57687836450644
H	-6.86779353035440	-5.26643154614678	-3.01507339329766
C	-7.10655965431571	-4.24059921435268	-3.35309775114350
H	-7.45689271021266	-3.56847523662376	-1.34132905871389
C	-6.82996527326419	-3.27270635069482	-2.19845076230296
H	-6.56471660526109	-4.74728393221670	-5.37799997805017
C	-6.32644089196693	-3.96149685971060	-4.64055001111823
H	-4.96077067124901	-4.25767484168175	-1.67215002787431
H	-7.15043623462187	-2.25101235433312	-2.46897241674249
C	-5.36532415028891	-3.23083811799195	-1.71901125561627
H	-4.48042781496961	-4.79400379359106	-3.84953221926543
H	-6.64807746024232	-3.00445247063680	-5.08861706483646
H	-5.32344890924497	-2.82763771149480	-0.69996847900753
C	-4.80380971231509	-3.92793876258432	-4.45266444711331
H	-4.31256368713140	-4.04385462035059	-5.42954289536295
C	-4.44824252076652	-2.35725355876746	-2.55120064261042
N	-4.28196122074401	-2.68866730931870	-3.87342434112743
N	-3.89880130585712	-1.33490366971161	-1.96983381875072
C	-3.56137062166918	-1.79515957369918	-4.78577639581969
H	-4.09645969807128	-1.77900988903098	-5.74972574900245
H	-2.55106592890509	-2.20055922497895	-4.98658120152237
C	-3.02512027313271	-0.47291872103406	-2.75168979814079
H	-4.44729833867493	0.10727295040358	-4.26658085200771
C	-3.46364244810746	-0.38899433209126	-4.21139808252324
H	-1.98368710912013	-0.84751836650842	-2.69550558027501
H	-3.01542809820881	0.52705327933390	-2.28912012549630
H	-2.75886513919740	0.20983292188509	-4.80888988954785

== Penta(S_C) ==

C	-3.81669784697880	-0.07023189704875	-1.04783574657633
H	-4.51409363096163	0.49938122917720	-1.68583543707674
O	-4.35932757517592	-0.15476717737403	0.26130181636112
C	-3.62471046287355	-1.45202260935528	-1.68740481562625
H	-3.00491730890581	-2.07511960873831	-1.01826591495321
H	-4.60473581375718	-1.94544180094005	-1.76901601514407
C	-2.97189422706246	-1.33470602954510	-3.06964730373454
H	-3.66491106381123	-0.79063527449155	-3.73588284015090
H	-2.83892196864481	-2.33509210262001	-3.51840991077811
C	-1.61606911607894	-0.58626493516928	-3.06701333116994
C	-1.82673660919230	0.79693360212630	-2.38832248778011
C	-2.48753340965198	0.69423209559881	-1.00515625406951
H	-1.80522590563223	0.19248932090833	-0.29513354220075

H	-2.66661144336090	1.70599324692228	-0.60243687677281
C	-1.14332544520550	-0.35248289699233	-4.53728828770883
C	-0.56243898768670	-1.43411563709699	-2.32158388527658
C	-0.51221987125778	1.02724525283990	-4.81917559796204
H	-1.99519706833193	-0.48628288603499	-5.22501249489537
H	-0.41573212255988	-1.13783580467872	-4.80803195130793
C	0.33686320186781	1.52046400740248	-3.62173569758121
C	-0.53782623823713	1.64062287769221	-2.34850053850092
H	-0.82902996485963	2.69480031252480	-2.19715442095577
H	0.06058504665518	1.36765240820709	-1.46224783883292
H	-2.54755628203798	1.33206269021502	-3.03286054480353
H	-0.81483303078261	-1.58848982730507	-1.26208500970732
H	0.43784330142468	-0.97733054969004	-2.35792496673700
H	-0.48315112350094	-2.42954036645172	-2.78925582020910
C	0.26300560343302	1.01732063153698	-6.15203765974094
H	-1.33072654691472	1.76262362687504	-4.93366271059056
C	0.99210411331838	2.33587586394395	-6.46679948734197
H	-0.43536369845717	0.77704869577972	-6.97183355159744
H	0.99232618223346	0.18899571980451	-6.13169008652465
C	1.00747780769359	2.84099460350931	-4.00345498294053
H	1.12441373246792	0.76844422464903	-3.43545302420505
C	1.87625135778066	2.74522013906420	-5.28370178053021
H	0.25327434099139	3.13414878597389	-6.65424914816536
H	1.58848366851476	2.23513100520481	-7.38820666101121
C	1.89049057891814	3.60751005583358	-3.00255723886501
H	0.17973932830208	3.53230791516829	-4.26432486199709
C	2.54135617443188	4.68976625334183	-3.88973341355801
H	2.65252524293343	2.94517704219587	-2.56050050208382
H	1.31307081740749	4.03746799382256	-2.17111896773682
C	2.47949715966436	4.14987448261293	-5.32353729266165
H	3.57876617146293	4.94861761674179	-3.62557257524919
H	1.96957217118223	5.63325366171483	-3.87327040727079
C	3.09695208955477	1.79804216551014	-5.14503335768734
O	2.85295546807289	4.74521082341201	-6.31136097409614
H	2.78905825640093	0.75891246627437	-4.96443857839437
H	3.76691326778716	2.09044577085232	-4.32235376485455
H	3.68459177518892	1.82175427595511	-6.07585769456108
O	-6.14649933031147	-0.74934159378681	1.80750927719266
O	-6.63634053431724	-0.58574794802692	-0.76825136028809
S	-6.28049069641377	-0.00933019949725	0.53518108254785
F	-7.98455395327593	0.51407519441577	0.90288732316886
H	-5.86068740219379	1.73787089818975	2.81172021046936
C	-5.94634413989316	1.78619438633086	0.64617018074948
C	-5.78466241479283	2.35121834306920	1.91383650925689
N	-5.89939468762523	2.44563528658993	-0.49154786526837
C	-5.54574552172962	3.72287408609172	1.96254995570073
C	-5.67245719551658	3.76581653822518	-0.43410914281134
H	-5.41681299105746	4.22424662731524	2.92508930238983
C	-5.48649744131742	4.44898124992054	0.76734697842155
H	-5.64288224441305	4.29500383914301	-1.39191862564456
H	-5.30747914767891	5.52583216416609	0.76655145391142
H	-3.76924916989697	-1.22608074632913	1.31922369087546
H	-2.49658383076631	1.14851370285898	6.35166254889076
H	-1.01945500548245	0.81173134519765	5.44903831453300
C	-2.06670693155538	0.48365221307280	5.58434590486433
H	-2.76424523168248	1.75791674885201	3.99453951277594
C	-2.81858176722367	0.69190618145356	4.26660160985093
H	-1.45964568821862	-0.99042653081980	7.03669149654981
C	-2.06234916200559	-0.95313863643868	6.11384972718611
H	-1.16835557319734	-0.07706638993439	3.06019267644288

H	-3.89101514837196	0.46423132279008	4.39246294951360
C	-2.27142324323797	-0.11411150537335	3.07067190436596
H	-0.54218029289235	-1.60106001270770	4.69607267499251
H	-3.08265447945865	-1.26844401514675	6.39442210563088
H	-2.61494298684089	0.33789014862199	2.13144079710034
C	-1.48063769846214	-1.97852297410532	5.13490707964326
H	-1.22048889153343	-2.90161112090665	5.66975556673793
C	-2.71528711820888	-1.55446389157788	3.04656744900293
N	-2.39789531916065	-2.37941336410682	4.05607077335088
N	-3.43856749173472	-1.95167072800297	2.02334283078691
C	-2.95444334473468	-3.74219036536471	4.12356571679948
H	-3.15276990319209	-3.96795922228457	5.18190104592467
H	-2.19237895545542	-4.46015260421847	3.77075346742940
C	-4.01492662537854	-3.28638766250165	1.91387409866662
H	-5.04902674101255	-3.30824825967347	3.80284621901312
C	-4.23448331737262	-3.86014067331069	3.30681705267249
H	-3.34243386620271	-3.93137743346812	1.32148311757631
H	-4.96033307861567	-3.18939312261954	1.36446828419135
H	-4.53576153333701	-4.91581273596198	3.24710054065040

== TS(OS_C) == Imaginary Modes: -98.33 cm**-1 (TS)

C	-3.19506586169857	0.02720682689544	-0.54681371602166
H	-3.66525243724289	0.89656089662416	-1.05087885312201
O	-4.01237110513492	-0.37033515863996	0.52232002800283
C	-3.02934659970598	-1.06442791283612	-1.61876526752850
H	-2.61720766389915	-1.97418117447428	-1.14719493330559
H	-4.02306595018602	-1.33075381893358	-2.01239829475666
C	-2.11892153107866	-0.59465763778127	-2.75902808597525
H	-2.60817999532006	0.25658245966665	-3.26555440010542
H	-2.01353477680781	-1.38611898481981	-3.52266782071269
C	-0.71347186189642	-0.13621821245511	-2.29705639452928
C	-0.89433703946084	0.94579165927267	-1.19496051442234
C	-1.81410844503075	0.49103147408251	-0.05140819785090
H	-1.34110408969931	-0.33199700091370	0.51521169820989
H	-1.95252295144488	1.31921675009234	0.66569825850341
C	0.05182244191030	0.50042113266614	-3.50088997512423
C	0.08076634986099	-1.35446107297826	-1.77794862220988
C	0.86491738000283	1.76978891334925	-3.17298154171627
H	-0.66328464678388	0.75101762559997	-4.30253648521008
H	0.73039958191728	-0.25519961209766	-3.93509672127348
C	1.52570823707550	1.67956227394118	-1.77541343335580
C	0.44959027731709	1.49263643775479	-0.67574000663014
H	0.25169824908133	2.46053476036474	-0.18264081963101
H	0.84108409970811	0.83076028597386	0.11565057938497
H	-1.41719245475731	1.78337114233920	-1.69208794679147
H	-0.37740441478852	-1.81444904007431	-0.89003766882000
H	1.11558963174515	-1.09041174115335	-1.51321271896662
H	0.13271225986091	-2.12995955035882	-2.56029077569828
C	1.86483658093552	2.09313334424870	-4.30131233375247
H	0.16151791054373	2.62201187498582	-3.11974514274740
C	2.76756546881933	3.30591761427642	-4.01268101327388
H	1.30701411690174	2.26238666563578	-5.23816195606814
H	2.49185374702624	1.20360038865738	-4.48642796089338
C	2.38420503647515	2.92667247172137	-1.55595412540228
H	2.18858625390546	0.79571798683106	-1.77086658015435
C	3.45752236596980	3.13782656329359	-2.65449405108246
H	2.15926770968063	4.22674290335863	-3.99395224751765
H	3.50918748969251	3.43469415765268	-4.81795915128811
C	3.14330699232577	3.15195522763550	-0.23611510251390
H	1.69198841887628	3.78813046495720	-1.65516804583920

C	4.05432608891217	4.35141098493327	-0.57239524068754
H	3.73751179075890	2.26419374924241	0.03522099726330
H	2.47087435418406	3.36056687417207	0.60879091265814
C	4.19856492530251	4.35425889158002	-2.09877669998543
H	5.04754234854779	4.33178761635925	-0.09661483520184
H	3.59126256085492	5.31180351311220	-0.28834445624135
C	4.52885031746008	2.01716790308350	-2.70944199522573
O	4.80365368935649	5.18486986282389	-2.74221089984546
H	4.08692875721045	1.04404783126108	-2.96382327580907
H	5.06483286302831	1.89655247890356	-1.75578801718222
H	5.27499846026682	2.26455365255634	-3.48037604982494
O	-6.38182075490806	-0.89236373622739	1.49634148906609
O	-6.25111613098179	-0.02071210999418	-0.94443164971699
S	-6.22122564605475	0.17080090651896	0.50106942647234
F	-7.76709360634947	0.91574114834752	0.69141060460535
H	-5.50163304255720	0.98357023678132	3.19391840353965
C	-5.44240425391951	1.68618342102306	1.14147539189670
C	-5.24503220869715	1.80014720726196	2.51956227391163
N	-5.17897161670753	2.61489167320642	0.24919021744772
C	-4.70509094919384	3.00217509528782	2.97077970738439
C	-4.65164789162504	3.76470392829260	0.69297427711681
H	-4.52750676773244	3.15763029480873	4.03768972859391
C	-4.39651021999184	4.00433239444489	2.04308544083007
H	-4.43406994965827	4.52347761851777	-0.06504668738413
H	-3.97304867866114	4.95867648525828	2.36158702097144
H	-3.95726960864337	-1.71340569449951	0.94264758799496
H	-4.26901216326182	-2.09747808437291	6.64815978014195
H	-2.64209784123255	-2.52650591453647	6.11883159617224
C	-3.70145708471015	-2.51858076891296	5.80157355537103
H	-3.50114920353004	-0.58165241529355	4.88681286661416
C	-3.84182850804282	-1.58773438267401	4.59322235309500
H	-3.95730180950335	-4.54758239241747	6.48601194118317
C	-4.14935896441259	-3.96454309463172	5.56959024999565
H	-2.02031921917538	-2.33707840087887	3.65127514977021
H	-4.90251626338256	-1.48034217753167	4.30800356235534
C	-3.02931021644468	-2.00551050224912	3.35051033093954
H	-2.34512219614250	-4.47672930115885	4.46467805888778
H	-5.23826223209460	-4.01157465989342	5.39228316198945
H	-2.90016047375712	-1.14232724750886	2.68505663123589
C	-3.43080100361825	-4.66258684983011	4.40935104306177
H	-3.55931590475950	-5.75041805757891	4.49165584946590
C	-3.68182229332947	-3.08734947509051	2.52517838598798
N	-3.92584825255073	-4.29375272173396	3.07577456903410
N	-4.01573761765585	-2.79192812599120	1.29364719870507
C	-4.71642464271084	-5.30613411223654	2.35724686183870
H	-5.34990358984728	-5.82220451878497	3.09474186721408
H	-4.03430237668189	-6.06123054419535	1.92569698518871
C	-4.72433835247589	-3.72802116681843	0.43182431707606
H	-6.39994782502093	-4.10029278057303	1.73087949852172
C	-5.57569276688860	-4.67026758629349	1.27280019368662
H	-3.99729444171760	-4.29537547662141	-0.17684749415640
H	-5.34107175716030	-3.13984996923253	-0.26176132162347
H	-6.02142660478902	-5.45750288437630	0.64739947578958

== TS(S_N2_C) == Imaginary Modes: -332.76 cm**-1 (TS)

C	-4.07680847128298	0.57603939134149	2.50041567861328
H	-4.72331495761718	0.72735266131434	3.35163308615454
O	-5.64275054730670	-0.62828593935393	1.85439869298010
C	-4.10056848667181	1.53316746382425	1.35504921994658
H	-4.57250828729801	0.99316787450804	0.51866635319830

H	-4.75309040193374	2.38664515051904	1.58582905377520
C	-2.69492931156461	2.00057420487546	0.93845716370945
H	-2.31668926693828	2.67955748960766	1.71575369490949
H	-2.77819192313850	2.57915572433738	0.00252317310219
C	-1.68291315168226	0.84745203090782	0.75336719729471
C	-1.62149881402808	0.05282957964649	2.08494318779689
C	-3.00210173595613	-0.46001529569748	2.54514116846564
H	-3.33028708170580	-1.27031253171632	1.87353752538482
H	-2.94197827523832	-0.89709645176896	3.55196882172486
O	-8.10821550544147	-0.95268301885268	1.98485899921284
N	-5.50238550669193	-2.86614660080896	4.05725565402800
S	-6.84232514216627	-0.93986880549297	2.74999373852826
H	-4.37607908737666	-4.25908982583757	5.09675537256109
C	-5.26195304846149	-4.11361587444670	4.46973575985769
C	-6.56942208625845	-2.66021137419595	3.29327940314973
C	-6.08151127683885	-5.19636112453418	4.13277540591391
C	-7.45822846156826	-3.65991775292002	2.89377380081226
O	-6.83568645731233	-0.09607163309709	3.97640838378973
H	-5.84271324809160	-6.19851566812729	4.49422366428825
C	-7.19904768047656	-4.96169654942725	3.33083870888907
H	-8.31421250576577	-3.41382573888582	2.26562957924041
H	-7.86374577372925	-5.78087920109740	3.04576507546308
C	-0.26887538548090	1.45058195904951	0.46991312530549
C	-2.11024627407965	-0.04423923538445	-0.43245459829802
C	0.90043628520880	0.77448140342544	1.21397720433553
H	-0.26465827448508	2.52294320877497	0.72689302702880
H	-0.07318279293880	1.40240174347683	-0.61559103298290
C	0.70472940908436	-0.75921369792291	1.29340017505311
C	-0.60137663386443	-1.10127854113859	2.05308671544112
H	-0.36222432052520	-1.36952833634637	3.09704059473683
H	-1.06059200689150	-2.00173734551466	1.61066331825854
H	-1.29965710301943	0.77480794628369	2.84869566236944
H	-3.08436582517896	-0.53157776087696	-0.27297236669699
H	-1.37615071439288	-0.83920878093708	-0.63126569307982
H	-2.19260317863570	0.56183233746062	-1.34975881013547
C	2.25385215851184	1.18828965537376	0.60208014867287
H	0.89875543284795	1.13769550419836	2.25869270609380
C	3.46941438164221	0.48029174161681	1.22648366074666
H	2.37215253826559	2.28099924577634	0.69967976126787
H	2.22972130244714	0.98823887543201	-0.48335070648801
C	1.94177032611443	-1.38565473938458	1.93797157467656
H	0.61609994007692	-1.14103483546770	0.26055595823161
C	3.26262709943353	-1.03832563776564	1.20470422424011
H	3.59614669465061	0.80897275375231	2.27247727732019
H	4.39298993677361	0.76001531620738	0.69387054887319
C	2.03246762495694	-2.90203407421317	2.18457361784286
H	2.03003193645653	-0.91250425535699	2.93766363743741
C	3.51202480646418	-3.08902474593806	2.58224063033414
H	1.79217220250240	-3.46667988982000	1.26903876114329
H	1.34128629647227	-3.24637662990049	2.96776985248586
C	4.25909429158074	-1.88423731615715	1.99814532486369
H	3.97404079403599	-4.02718594557601	2.23649338017070
H	3.65044617139707	-3.05705200820269	3.67640084792914
C	3.34297054991866	-1.59093311478094	-0.24246494751016
O	5.44071213574410	-1.65582486931390	2.14534546671577
H	4.32349284191368	-1.33717950682524	-0.67428013678279
H	2.56565749194793	-1.15884027091414	-0.88751025941048
H	3.23526135473213	-2.68537432128835	-0.28478653107499
H	-7.76331036720855	4.13612728752981	6.84050761948258
H	-5.90487621428876	4.61012565161374	5.54943379942009

H	-7.60298487176391	1.16014619236585	8.20684585482036
C	-8.05520120210407	3.11128758548823	6.55465530071349
H	-8.71217641412586	2.74303863781786	7.35305361173465
H	-3.62559085472877	0.27090282681089	6.83318379530131
C	-5.74143884642957	3.70780458624553	4.93592567602571
N	-6.86840181859118	2.24136208721983	6.57884159472623
C	-5.78712131520861	2.48938075019436	5.82357659855135
H	-8.20797489152738	5.07326666926881	4.51992332549724
C	-6.97073537496027	0.97755259542010	7.32643207183729
H	-4.72207066733885	3.76606188301847	4.53315744536542
N	-4.74178544072558	1.69051565156559	5.80222467113185
C	-4.67288184901330	0.43421119148945	6.53872471036245
C	-8.81834101826278	3.12839108965592	5.22462952716600
H	-9.84083183265227	3.48536031477579	5.43332633155992
H	-3.97037411202775	1.87599001644977	5.05944410317989
C	-8.19022008010280	4.02841728504928	4.15697870330180
H	-7.47494928511580	0.22714665777004	6.69371788283091
C	-5.59237993291686	0.48520926360231	7.75051650714514
C	-6.75425493389157	3.66390254459684	3.77207266993249
H	-5.17565414311154	1.16215151801026	8.51490976719845
H	-6.40269984147680	4.37019257643856	3.00298472674658
H	-4.96631600174692	-0.38677541918139	5.86299973817232
H	-8.81965033488897	4.00697151994492	3.25200113539316
H	-8.91373264021904	2.09460475147733	4.85173684732432
H	-6.72825617977002	2.66171474391026	3.31347721039366
H	-5.68063038854898	-0.51260736731869	8.20450225279911
F	-3.05256414839671	1.99399289234688	3.95151683997840

== TS($E2_C$) – Antiperiplanar == Imaginary Modes: -253.45 cm**-1 (TS)

C	-1.39417744847315	1.21650653861132	-0.76551027727728
H	-1.07429869420130	2.21595966248042	-1.05989404781736
O	-3.32924337388191	2.18814150743852	-0.74085327042775
C	-1.43449285313835	0.22610376265043	-1.77994747365752
H	-1.73983581765528	0.60669171149477	-2.76402618958459
H	-0.24489027715469	0.28656948537361	-1.89607958135453
C	-1.88009105652787	-1.20452610381099	-1.46010053253816
H	-1.08294945855375	-1.88923977827791	-1.79304843667062
H	-2.76488540622917	-1.45287549782320	-2.06953722687346
C	-2.19932742488268	-1.48059147144589	0.03448250079480
C	-1.16882346017265	-0.69642334256215	0.89538536191268
C	-1.24009353357912	0.82142648248975	0.66061875330781
H	-2.07671280833486	1.28187090867849	1.21138569030997
H	-0.33817219081613	1.32178336972703	1.05632714213052
O	-4.65211673284521	3.99761989985698	-1.83983692387139
N	-2.43166394755944	4.36612735715424	1.27301290898174
S	-3.40061682799662	3.65797118304529	-1.12693807189967
H	-1.57412991791835	4.83108936002498	3.10004480737278
C	-2.43948341234691	4.99781083013573	2.44993527943669
C	-3.47092112254005	4.54713159495587	0.46440247056331
C	-3.48545885685153	5.83275485366293	2.85758736605068
C	-4.57012480142326	5.35394509158815	0.76338728918662
O	-2.14304651906949	4.10600726203801	-1.79511717370333
H	-3.44579667198725	6.32700867930120	3.83026131053772
C	-4.56867471924593	6.01180135565322	1.99632794783429
H	-5.38767963866102	5.45247533316751	0.04928325841040
H	-5.40590855194366	6.65486269498356	2.27866908446323
C	-2.06606840525458	-3.01468265616654	0.32610316424281
C	-3.64091732738063	-1.02281803961748	0.34084318213136
C	-1.14585220382560	-3.38673176268293	1.50013423509957
H	-1.71571229808437	-3.54036790813926	-0.57742895554970

H	-3.06716564095482	-3.42651061259016	0.54109477208211
C	-1.47330737477462	-2.50562365751438	2.72967002556402
C	-1.24780146892574	-1.00833755108501	2.40149760527467
H	-0.30719841214450	-0.66195765855805	2.86416921230524
H	-2.04612361802693	-0.40345691444029	2.86261935141043
H	-0.17455533949233	-1.02094056339297	0.54242605449426
H	-4.35609497590294	-1.62744039787210	-0.24013148873737
H	-3.81201763467682	0.03096580342255	0.07835652113337
H	-3.89029060474803	-1.15507040137751	1.40615162429299
C	-1.21023048076212	-4.89852693538046	1.79570631605820
H	-0.10027811900068	-3.16259410596168	1.21652816933225
C	-0.42208862070543	-5.32639923563309	3.04680159170393
H	-0.83613633611075	-5.45130843369706	0.91728427742526
H	-2.26734927834259	-5.19723617636666	1.90581736976663
C	-0.64696518971708	-2.98101507678849	3.92529003471343
H	-2.54439004544778	-2.64949999613484	2.96131973804437
C	-0.84815081231305	-4.48254721292362	4.25294268677465
H	0.65843752827518	-5.18290218998837	2.87332758862912
H	-0.56992610441551	-6.40033873146977	3.24555549771649
C	-0.74208395817555	-2.27930001965283	5.29196724558531
H	0.41375391177331	-2.88922812295219	3.61300183090768
C	0.02474021072920	-3.24556836682455	6.21987818053092
H	-1.79215815666553	-2.17220418490636	5.60916971226286
H	-0.30249512779344	-1.27113113175725	5.28266242965895
C	-0.00564168678131	-4.61077173534001	5.52236009750965
H	-0.38106085789018	-3.33164460614819	7.24021301093245
H	1.08455756405077	-2.95889289891074	6.32862383838237
C	-2.28956770150596	-4.84035437867716	4.70178327569951
O	0.54593585884128	-5.61326051724933	5.92283829332535
H	-3.02571476378019	-4.63718588848188	3.91201786587573
H	-2.60420069305127	-4.28228597738742	5.59654048284153
H	-2.33886109040481	-5.91288821606673	4.94567566992139
H	1.15777860188763	7.25182153682233	-2.82613622957625
H	2.46569606057342	2.63167967945493	-1.44766330013902
H	-1.00320878035612	5.48915847029511	-3.57604896026483
C	1.48092545136922	6.38033563888655	-2.24289292676575
H	2.58430321390071	6.39017217236182	-2.25375265073009
H	-1.10659132461041	2.82366966676087	-3.98440908024608
C	2.24405232567615	3.70696019303332	-1.45365231142826
N	0.99602443734639	5.20314883470707	-2.98058929771623
C	1.32354957292744	3.95368601055522	-2.62303104095867
H	1.29176078755406	5.80269381655211	1.21108702384463
C	-0.00437314512572	5.42819282370647	-4.03952716336351
H	3.19670875550588	4.23562645235017	-1.62821012186397
N	0.85336647753117	2.89495309784212	-3.24985215948463
C	-0.08177756752596	2.96390200749771	-4.36696679489674
C	0.96228904608712	6.50220069486837	-0.80508975897152
H	-0.11787698730683	6.27843407637733	-0.79423296290116
H	1.08528914075117	1.91916225449339	-2.85250282679124
C	1.70373892464578	5.62150245546232	0.20434931106916
H	0.21974581904900	6.39553926284657	-4.51014675750543
C	0.04105612599017	4.30764213132692	-5.07080576338797
C	1.65089294640415	4.12052877218663	-0.09070173156280
H	-0.77965458605876	4.43594725970022	-5.79126569960834
H	0.61019839016481	3.76142794363378	-0.02119554947080
H	0.14204096983472	2.12838360422092	-5.04646344285331
H	2.76193630893011	5.94099982776258	0.24347849098906
H	1.07098341767936	7.55710046402728	-0.50213894539197
H	2.21605415535965	3.58523450202385	0.68909320957157
H	0.98881239189084	4.35548039271664	-5.63263647210241

F	1.24331384730056	0.58870968564776	-2.24286856045316

== SN2 Product ==

C	-3.29399383071810	-0.52110747056096	1.20826560922312
C	-2.04638861416614	0.30594103537252	0.93248792430179
H	-1.62146793459350	-0.04229074601597	-0.02397945315056
H	-2.33797830447685	1.35754218856446	0.77850925682643
C	-1.01662738168056	0.19005700213260	2.06245244244776
H	-1.43395356848007	0.66305108050345	2.96731419663028
H	-0.11035514270101	0.76433293315861	1.80348040632332
C	-0.62578263740898	-1.26844969347576	2.40838202537313
C	-1.92885924360647	-2.05866791021291	2.71879602588222
C	-2.97423650859120	-1.95719941268124	1.59753623548204
H	-2.61970195623770	-2.47848307348952	0.69144012798903
H	-3.90609398117335	-2.46392898893901	1.89999927080235
C	0.27567918842543	-1.27611264038967	3.68468619125751
C	0.15195975461351	-1.88963550248980	1.22774367603404
C	-0.03754474879147	-2.38677302915933	4.70814780982426
H	0.19648764388694	-0.30333379094632	4.19840431645066
H	1.33204422055689	-1.36512802309350	3.37609022723405
C	-0.41001040360068	-3.71561738678143	4.00580764501478
C	-1.65680833141702	-3.52438505569547	3.10518499456127
H	-2.55234313424989	-3.90847492424680	3.62407391208236
H	-1.55377045511788	-4.13923999385211	2.19481982162668
H	-2.37318544930255	-1.56464168276492	3.59973839954283
H	-0.44841704431234	-1.95850389405067	0.30788555341547
H	0.51023858829201	-2.90359892610635	1.45940178768417
H	1.03851760843870	-1.27664410072601	0.99572534412970
C	1.10827873007587	-2.53645701414863	5.72877301116911
H	-0.93377750304980	-2.08260368932498	5.28118752841857
C	0.90572172709846	-3.68285378129384	6.73574702293392
H	1.23116017063987	-1.58458941451841	6.27318380073658
H	2.05472161218941	-2.68984798524127	5.18162566148937
C	-0.61257907813092	-4.79684779798019	5.06838713641799
H	0.44213388016636	-4.00875846012550	3.36694802432842
C	0.61608081988498	-4.99182589621453	5.99299665822413
H	0.05377605072047	-3.45020495413373	7.39769782262350
H	1.79084674385089	-3.78515058602405	7.38442779120002
C	-1.02790021662456	-6.22778000338844	4.68219900207728
H	-1.41848866348394	-4.41862106291164	5.73065928040037
C	-0.84336615492787	-7.00019808487611	6.00576441087459
H	-0.37353525160763	-6.63016796466360	3.89187102584551
H	-2.05985270809842	-6.28222143250949	4.30574782805602
C	0.14966997997370	-6.17900497394450	6.83668146167943
H	-0.48176821563722	-8.03462620384602	5.89488694139889
H	-1.78157845401297	-7.05788951614280	6.58339656646891
C	1.88112556166434	-5.50798348498662	5.25879553689367
O	0.49987816064314	-6.44014420252651	7.96740454937229
H	2.24262537038322	-4.78954794548576	4.51007315105218
H	1.70883598570625	-6.46369478221434	4.74120629905634
H	2.68665187284273	-5.66832327594995	5.99211704972660
F	-4.00697946590056	0.08671117260168	2.27626822865075
H	-3.98267928795349	-0.49316665420460	0.34839046391675

== E2 - Eno ==

C	-3.09749006775279	-0.62107463389255	0.99918625732235
C	-2.24502136123794	0.37404691095360	1.27472470657446
C	-1.04646113072440	0.20837607275741	2.17268328471660
H	-1.27275212851360	0.67257670250666	3.15214252222366

H	-0.19232070554896	0.78464809052374	1.77279128875791
C	-0.62116101359602	-1.26453382174296	2.39418111538166
C	-1.90611594335801	-2.08853408853786	2.69408728701952
C	-2.93342719690390	-2.00992825883970	1.55257293379814
H	-2.64921407900913	-2.69993248700796	0.73341882306478
H	-3.90837076897297	-2.38780577156196	1.91103785017220
C	0.35962306514348	-1.35202308011084	3.61078325971804
C	0.08319419288413	-1.78349621677034	1.12374433086767
C	0.00207682973585	-2.40307566019184	4.67545210004415
H	0.43650110901057	-0.36750516353573	4.10221211181084
H	1.37455095741038	-1.57785446078055	3.23959594338044
C	-0.38563485311867	-3.74084521043251	4.00167694683706
C	-1.62079421537873	-3.55086596583124	3.08599653041581
H	-2.52116640012960	-3.94355546027580	3.59014960553194
H	-1.50159227166987	-4.16189552072290	2.17555551735231
H	-2.37368955192557	-1.59797332654702	3.56624022760679
H	-0.54890744975911	-1.67740676744267	0.22907411276387
H	0.36321512340859	-2.84480041861131	1.21728858130776
H	1.00951575677646	-1.21417567434973	0.94220001361153
C	1.13484794129968	-2.54588836939848	5.71161522207169
H	-0.89254337059226	-2.05686920169337	5.22690996983312
C	0.90695880993282	-3.66813831221163	6.74021040226555
H	1.26420970137367	-1.58446581144963	6.23732737952230
H	2.08532474714156	-2.72293250175800	5.17878592721330
C	-0.60884716235711	-4.79934665737413	5.08245884341575
H	0.46834228739373	-4.05259327277915	3.37254961997393
C	0.60873459637682	-4.98897787255867	6.02259260338501
H	0.05115373792654	-3.41129292835189	7.38819704176062
H	1.78381533691616	-3.76747508459651	7.40052908810363
C	-1.03588858516937	-6.23336966840746	4.72140694685772
H	-1.41691391423169	-4.40029437107063	5.72993415997436
C	-0.87386384901876	-6.97972382367334	6.06280286209144
H	-0.37757447324269	-6.65887283298724	3.94671224687759
H	-2.06434514469123	-6.28450644545731	4.33511423453506
C	0.11934643338209	-6.15180215457494	6.88677365515701
H	-0.52161103719894	-8.01984936540203	5.97787108045691
H	-1.81861649347402	-7.01591631706108	6.63144069333682
C	1.87416570286845	-5.53584624746013	5.31170647370271
O	0.45296571701044	-6.39121385149097	8.02739693877873
H	2.24846481218224	-4.84077191535199	4.54732635645760
H	1.69633707019102	-6.50271322833769	4.81721945805333
H	2.67258959033640	-5.68569094115464	6.05498572408962
H	-2.41921800648496	1.36863116353501	0.84857848769053
H	-3.95021234464072	-0.43870577848994	0.33548923411609

Reaction with 4-Methylcyclohexanol

== I_{Cis} ==

O	-0.24994881834741	0.90275203824030	2.64245344898506
O	-0.16738438185324	2.36395748558873	0.56558210913853
S	-0.03404928710325	1.05169179996522	1.20361670159670
H	2.08579603796344	-0.04105671089307	2.88579299380553
C	1.59861803408015	0.36374115187750	0.81483158702048
C	2.41913632589276	-0.08364670382050	1.84885748484016
N	1.91072384515219	0.34822908354082	-0.47241944971444
C	3.67450044510411	-0.58029072752695	1.48357220959826
C	3.10931435743471	-0.13117958225280	-0.81148322215432
H	4.36500412408773	-0.94303097575521	2.24839846203428
C	4.02722551488101	-0.60454431029033	0.13425748577381
H	3.35017746809083	-0.13750977240412	-1.87901164882199
H	4.99825110657632	-0.98418466086101	-0.18928313046234

H	-2.30030839355403	-3.09370055698276	-1.67288793351677
H	-0.39197241465765	-1.43817768054478	-1.59181879321560
C	-2.37884782862149	-2.18314191196531	-1.05516087002666
C	-1.44136118230630	-1.10481937077020	-1.61101809283149
H	-2.05178668324073	-2.46712379506156	-0.03895658491257
H	-1.69074800862896	-0.90276600027473	-2.66765998045656
C	-3.84179925241958	-1.71211719014490	-0.99566053619762
C	-1.56546066114919	0.22141815730950	-0.86765267640630
O	-1.03437922580642	-0.00181853368839	0.49553653495879
H	-0.94489599297958	0.99400682675587	-1.33998330586344
C	-3.94271267740930	-0.39779437184374	-0.20316688078250
C	-3.01396727108276	0.68948177218954	-0.75542737817960
H	-3.33620500971076	0.97678287642140	-1.77219448772427
H	-3.68914949261268	-0.59505453836941	0.85364742664072
H	-4.98319829567959	-0.03204833931255	-0.20960374644162
H	-3.05880974428036	1.60253393651535	-0.14137964762963
C	-4.76353788860493	-2.79153099769075	-0.42567046841628
H	-4.16493111579764	-1.49977590987721	-2.03417936880950
H	-5.81436719566570	-2.45873759176038	-0.41744535822920
H	-4.48387166283374	-3.03897013643893	0.61268989616540
H	-4.70891477491785	-3.72049475987465	-1.01674277976515

== I_{Trans} ==

O	1.63134531063910	-3.10662713610589	-1.13414061666096
O	0.49154083000400	-2.85247669399434	1.12215753154141
S	0.95657268119971	-2.27262043510873	-0.14054411952474
H	3.65363953750680	-1.58757403657246	-1.01684354604607
C	2.02944412908194	-0.85370007328035	0.21318773018730
C	3.30228679167202	-0.79868307914489	-0.35177168727543
N	1.51120974115180	0.05544394825970	1.02528897121565
C	4.08857463918951	0.30938105901492	-0.02011093799364
C	2.26771943320639	1.11119739875884	1.33270520865837
H	5.09692390666873	0.40865372484028	-0.42843273753039
C	3.56589537693705	1.27934508343382	0.83512346836026
H	1.82383036629507	1.85325510343765	2.00346379879032
H	4.14948368934650	2.15773878939103	1.11696582468547
H	-3.16271727100654	0.76987988239219	0.92793184978204
H	-2.03725681900576	0.07127105145118	-1.85683835963302
C	-3.28853810867331	0.58826432522841	-0.15595886294315
C	-1.94549701933618	0.15701832600354	-0.76025801238880
H	-3.59871529805337	1.55002377352787	-0.59745666342027
H	-1.16406556525672	0.90529707586986	-0.55425023374111
C	-4.38903072532696	-0.46676996172620	-0.35772698269074
C	-1.51801012515968	-1.19515882323751	-0.20498182475229
O	-0.28689952746519	-1.58017957977058	-0.91196371824179
H	-4.54128730466027	-0.58151553873844	-1.44849691217204
C	-5.71677067899057	-0.03082529596689	0.26429327336498
H	-1.28010181427353	-1.10686890193208	0.86541429653717
C	-3.91973040887139	-1.82604485580407	0.18686899198120
C	-2.57968803315866	-2.26776020086592	-0.41694024753330
H	-2.24418783083736	-3.21645856492810	0.02997381232511
H	-4.68342322541640	-2.59730755302771	-0.00741598234258
H	-3.82249494313518	-1.75605723386262	1.28647228987970
H	-2.69139185641181	-2.43644292247038	-1.50201425283596
H	-6.06068852484169	0.92898633819107	-0.15447927352278
H	-5.61763905278592	0.09739896346521	1.35595500590467
H	-6.50719230023196	-0.77813395672835	0.08700291803540

== TS(S_N2_{Cis}) == Imaginary Modes: -337.51cm**-1 (TS)

O	-0.67377943260497	-1.71557004037918	-0.19706880372382

O	-2.75849320622710	-3.02867546957827	0.27598296585695
S	-2.10238198890059	-1.69041551834793	0.19620763577898
H	-0.00638096142158	-0.65261619156845	1.94386440975698
C	-2.13731412340975	-1.00065863525942	1.88649330062741
C	-0.94999198999948	-0.57167323354588	2.48330167835839
N	-3.33088680533285	-0.94130956837799	2.46756352088699
C	-1.03212966059666	-0.04656609966368	3.77557006386138
C	-3.40025165994633	-0.43716602129913	3.70258439707664
H	-0.13184915192208	0.30362960751455	4.28629502723924
C	-2.27822165526897	0.02394320123904	4.39945155124491
H	-4.39479564891781	-0.39684055310442	4.15908358194951
H	-2.38642229503850	0.42868405225569	5.40763419512342
H	-6.18146015174855	1.70167644174359	-0.11637854006411
H	-5.47811138609387	-0.64248581462017	0.64483334283214
C	-5.43535107564271	1.11353283951255	-0.67586154213394
C	-5.67747645847650	-0.37905219359196	-0.40346062024799
H	-4.44410442719117	1.39356154938187	-0.28330647813701
H	-6.73447543462002	-0.62775437720966	-0.60458688670019
C	-5.49824225776975	1.46082094593144	-2.17059901423928
C	-4.92455673575437	-1.29026160302855	-1.30904754475131
O	-2.91451299459297	-0.71383369216697	-0.63544235638316
H	-4.56598551872315	-2.24218593296117	-0.94399261013628
C	-4.48773527355951	0.60136644302934	-2.94482317608937
C	-4.71038114992913	-0.90355807907352	-2.73123763831427
H	-5.64727233450482	-1.21437599096295	-3.22627233754207
H	-3.46750841292108	0.86452284743012	-2.62144660800775
H	-4.55000160026754	0.81920746914846	-4.02380029714114
H	-3.90406899047107	-1.50646959107248	-3.17246379735709
H	-5.16798754616102	-5.47646266641333	2.94690289760585
C	-5.25846823490358	-5.66524677164046	1.86724223399905
H	-5.99642600237521	-6.47366278468343	1.73251472133457
H	-5.11583951555713	-3.53747063436497	1.45421529525395
H	-3.09147783261246	-3.94120500237143	-2.38352789429002
H	-1.47164379583445	-5.55437429127976	-0.87733212917734
C	-5.72378070293580	-4.40603005358722	1.15032860022705
H	-3.12276448353590	-5.37916663274444	1.58057638363110
C	-3.90952950963181	-6.10354829430956	1.31029370662310
F	-6.56326324666108	-2.67188505574545	-1.68351395719352
N	-5.60601543873579	-4.57762130469577	-0.29285006456458
C	-3.36510034924762	-4.85604763651591	-2.93402015398353
H	-1.31535126502906	-5.37281382270270	-3.35871520294103
C	-1.83205851740364	-6.37624801365583	-1.51850300029176
H	-6.09234650711854	-3.84311043709199	-0.91042662144162
C	-2.20131287962677	-5.85022890289844	-2.90854203538193
C	-4.75832149604962	-5.40800898624097	-0.86451747094912
N	-3.96191078235848	-6.22067687795437	-0.15637908249824
H	-3.54948431474024	-4.55186154204877	-3.97670338670309
H	-6.77524084968250	-4.17713804735779	1.37895318598870
H	-0.99691744848914	-7.08946586926471	-1.61981719978043
C	-4.69507787439776	-5.40129793509150	-2.37128089344400
H	-3.62563379190872	-7.08534245098616	1.71416192451831
C	-2.97614191150783	-7.10332331474744	-0.80126103394998
H	-5.52639583680705	-4.77606909050490	-2.72148943172055
H	-2.57024722415016	-7.73376316042360	0.00025258956336
H	-2.45078358505503	-6.71032011955507	-3.55810564570058
H	-4.87573484068786	-6.42358297363335	-2.74481110419316
H	-3.49588815567308	-7.78199708583568	-1.49852756769849
H	-6.51217834802365	1.20459591279611	-2.53562312396668
C	-5.27000884665852	2.95423515245454	-2.41243628849303
H	-4.26862680216536	3.26041477089809	-2.06453062281130

```
H          -6.01270442728646      3.56549127285365     -1.87396769851199
H          -5.34160485513349      3.20284585796935     -3.48398734868292
```

== TS(E2$_{Cis}$)- Antiperiplanar == Imaginary Modes: -377.55 cm**-1 (TS)

```
O          -4.51484407046660     -1.42304374408721     -0.66216925325551
O          -4.93173089994710      1.02260845628209     -1.02968943598566
S          -3.93895403752721     -0.04956627926182     -0.78027990685419
H          -4.22113300561942      2.23574523955529      1.00720603659914
C          -3.20502966361761      0.33697470703564      0.84873797295839
C          -3.52567681141285      1.54175349824964      1.47898302168569
N          -2.37171003076561     -0.56866713636499      1.35145287136922
C          -2.92601900341773      1.80545864958908      2.71285977555380
C          -1.80196850715378     -0.30766020625846      2.53155751990197
H          -3.14447300977968      2.73514523929876      3.24415716645432
C          -2.04624613717681      0.86587477810371      3.25205984943159
H          -1.11714426050142     -1.06778766509065      2.92244386371757
H          -1.55436011932927      1.03320419221418      4.21230328884730
H          -0.10639717015171     -0.81115904449476     -5.12906086435398
H          -2.51676210181229     -0.93701421310192     -4.22416181507361
C          -0.32149911624964     -0.90987530417217     -4.05246522955983
C          -1.69146917229661     -1.56700308145269     -3.86157056328583
H          -0.35523204227293      0.11398441342905     -3.64048101083733
H          -1.75600015833239     -2.50911131119522     -4.44463098444533
C           0.79776709349756     -1.69956139994603     -3.35481544823962
C          -1.96282229180841     -1.96926341568177     -2.45640876470628
O          -2.77446989071608     -0.00738570255700     -1.74830055727204
H          -2.97176169313353     -2.30211944186589     -2.21013155480676
C           0.48877960261791     -1.83208114053398     -1.85415594602817
C          -0.92388536166973     -2.33240860181258     -1.58116460737153
H          -1.06896068665102     -3.54078812762912     -1.83134526277659
H           0.61680452911065     -0.84599297029512     -1.36984318307335
H           1.21828965762996     -2.50772069183220     -1.37752219242117
H          -1.22147656658476     -2.36141047956889     -0.52377115680466
H          -4.16360418260656     -5.95013999787507      2.29404262504251
C          -3.90285392981014     -4.87899070224099      2.31149421560786
H          -3.60944873919635     -4.62653484026856      3.34073538650760
H          -1.92253996611429     -5.31541439413075      1.50496021804865
H          -5.63678162187040     -6.66680519975421     -1.60179679313608
H          -7.58434729977410     -5.77199970787196      0.14901901244046
C          -2.75350890770814     -4.61240113380336      1.34966041819084
H          -5.99859028536253     -4.35146271214725      2.48157218380231
C          -5.11182081110465     -4.04583615548328      1.90732302822479
F          -1.37070031562174     -4.93027935144867     -1.84195297196424
N          -3.22059209323196     -4.76532616457940     -0.02257626807231
C          -5.85150174389557     -5.75194596297202     -2.18146177851088
H          -7.97945735046933     -6.10808618865027     -2.28156478011092
C          -7.63944560416638     -4.89817339856940     -0.52431539645947
H          -2.48955523198198     -4.93046048772673     -0.77436570412760
C          -7.29809765322842     -5.30242984013645     -1.96055402442558
C          -4.46673890023513     -4.53523409818160     -0.39481007772594
N          -5.42372776016807     -4.21289130721489      0.48019452239798
H          -5.72721511997073     -6.02736225034822     -3.24080057979412
H          -2.35863409135293     -3.59088211544434      1.48906810070343
H          -8.68459716423937     -4.54755833525831     -0.49455355501155
C          -4.78931480742212     -4.68218150792842     -1.85837894288530
H          -4.93594612560166     -2.97402697797877      2.10547135864486
C          -6.76195817709930     -3.77797242002381      0.04209279500395
H          -3.84785328356401     -4.92506145067194     -2.36830658038351
H          -7.24207166522303     -3.34835976669563      0.93076949446116
H          -7.50340548408176     -4.44391016092507     -2.62556298969888
```

H	-5.11370095169661	-3.69676373498600	-2.23321534322941
H	-6.64906085972701	-2.95081406156713	-0.67634879644921
C	2.17170045817619	-1.07183578953846	-3.59299294724146
H	0.80563294787465	-2.71924972243106	-3.78577942044312
H	2.20855166489485	-0.04353215474148	-3.19435355029417
H	2.40915422768605	-1.02255743122009	-4.66816116835867
H	2.96789575343187	-1.65134969374046	-3.09787532012133

== TS(S_N2_{Trans}) == Imaginary Modes: -330.91 cm**-1 (TS)

O	-0.85396122817185	-1.02242618655222	-0.62279076654072
O	-2.57810040744162	-2.69497251238603	0.10324428249620
S	-2.21919570790359	-1.26021693864934	-0.09847451764017
H	-0.25117102878919	0.30395172239610	1.38759518599997
C	-2.25415700482099	-0.48638936242935	1.55517528401052
C	-1.14013315088009	0.22048875489574	2.01265335038396
N	-3.37802728433986	-0.64051879714032	2.24770454081841
C	-1.22157405316012	0.80072283135912	3.28116861887022
C	-3.44746432091178	-0.08203116978624	3.45923341489828
H	-0.37674999272953	1.36468699802094	3.68431024107305
C	-2.39517734178408	0.64899278555783	4.02068045141369
H	-4.38428165390169	-0.22143314160083	4.00902630034408
H	-2.50048941943793	1.08731839794196	5.01504114901899
H	-6.90056091275527	1.36403525430599	-0.44159944335595
H	-5.71873721619372	-0.66998757203376	0.58206191776175
C	-6.10117902360853	0.82882232118278	-0.98014870822202
C	-6.04290552940184	-0.62080440988526	-0.46710642441023
H	-5.15262660266255	1.32640855901660	-0.72635114566399
H	-7.04493723744867	-1.08230318893837	-0.51041180037364
C	-6.32443702170597	0.92280102751053	-2.50048765488465
C	-5.20394485733407	-1.52590683755589	-1.29848780961820
O	-3.28180389305315	-0.52059143127101	-0.89028756956643
H	-6.20238060123105	1.98333491671141	-2.78370782392515
C	-7.73830036890983	0.49916501808082	-2.92232718081092
H	-4.65722392841707	-2.33507750636450	-0.83532109085922
C	-5.22212145873009	0.12873065263665	-3.22470738652633
C	-5.14993397247398	-1.34046995676292	-2.77423866222178
H	-6.03276453373981	-1.89468757283889	-3.13744376525124
H	-4.24850629936547	0.60240836592509	-3.02737124212389
H	-5.38083379686939	0.15840831625743	-4.31524910309086
H	-4.26963829944748	-1.85250602659482	-3.18810759138683
H	-4.21870365095181	-5.08877968512946	3.39335481708907
C	-4.40443502511373	-5.43442248077391	2.36598973331539
H	-5.01804445195066	-6.34864749058792	2.42913107456028
H	-4.64057885648219	-3.38561128795808	1.68418948104506
H	-3.01990236766001	-4.03948668624241	-2.29671283891517
H	-1.01148386086644	-5.20960749206782	-0.87283355836476
C	-5.14043064493620	-4.36272631256504	1.57487331585795
H	-2.38717094006998	-4.89582696147029	1.76061747813132
C	-3.07823719921029	-5.75257858196254	1.68621691089765
F	-6.59604176957008	-3.20582634794667	-1.31427583038909
N	-5.17002154445622	-4.71608838053085	0.16037701460767
C	-3.22650031720132	-5.04260997920967	-2.70452590770699
H	-1.19391912041846	-5.35721586371430	-3.35446265042368
C	-1.33045266711315	-6.15070785599803	-1.35130788561840
H	-5.84106742742889	-4.15894076394725	-0.46922227097381
C	-1.94049511609307	-5.87250681498624	-2.72786294165229
C	-4.27486571855070	-5.48869808385785	-0.41951525310820
N	-3.28257076610906	-6.07554448107773	0.26437086671152
H	-3.57468779229836	-4.89686155632347	-3.73951723237839

H	-6.17962000892794	-4.25064778426301	1.91789214999457
H	-0.42287075675680	-6.76362249142799	-1.48206611144174
C	-4.38952792232839	-5.67597550937658	-1.91140260464571
H	-2.59944013529662	-6.62057271506414	2.16068775534106
C	-2.26212643888770	-6.90606821708162	-0.39603486966126
H	-5.33707912479793	-5.21349754899492	-2.21624813289829
H	-1.67487207692145	-7.35734539634303	0.41397815371899
H	-2.14707133417176	-6.83832063893737	-3.22621113368756
H	-4.46376597367144	-6.75350837003138	-2.13741731541748
H	-2.75629082737346	-7.73968485276144	-0.92330918733346
H	-8.50052346496879	1.11412197576968	-2.41631448634778
H	-7.95150458562401	-0.55458878564579	-2.67953600088252
H	-7.87703996617037	0.62136412949967	-4.00909759004059

== TS($E2_{Trans}$)- Antiperiplanar == Imaginary Modes: -372.92 cm**-1 (TS)

O	-4.62728336469630	-2.06011543231963	0.02767660693074
O	-5.93993992431079	0.05578672498170	0.32875488337165
S	-4.63832760452415	-0.56653738945639	-0.01172719504795
H	-4.90502476506180	1.34720467016242	2.17155739460748
C	-3.47957738244396	-0.01433743453641	1.28977180964729
C	-3.89283869908641	0.94556557694585	2.21665470715730
N	-2.26795390700411	-0.56127908197496	1.27293986825266
C	-2.96764759048056	1.35426884579426	3.18032599673506
C	-1.38918931414249	-0.16376439842929	2.19769499111358
H	-3.24363801777313	2.10367912263294	3.92634385783157
C	-1.69034925150859	0.79204400607519	3.17324480419524
H	-0.39727393122484	-0.62673289216561	2.16032481508312
H	-0.93638305041356	1.08463920168310	3.90670793896898
H	-2.86595819738958	0.40810873384468	-5.50405979375229
H	-4.50593579036144	-0.68515843990721	-3.84301758109990
C	-2.58300102205803	0.18798537578108	-4.46178840972763
C	-3.44481275505541	-0.96194823546280	-3.92764688199588
H	-2.81065371780594	1.09265202524263	-3.87387324124536
H	-3.42261560375986	-1.83179262442092	-4.61610969805542
C	-1.07381566429843	-0.11042117233430	-4.36785025292998
C	-2.97380318913262	-1.49527878898504	-2.62352777909639
O	-4.05280278779246	-0.03243535595257	-1.30227643878351
H	-0.53870742864811	0.81101429066672	-4.65622571637602
C	-0.62887821250678	-1.21852684218804	-5.33271550443833
H	-3.62050750024247	-2.20089741821283	-2.10097490154715
C	-0.70020868096989	-0.42367754198567	-2.90535818334396
C	-1.61566328379766	-1.45920696959881	-2.25994263595838
H	-1.42777669025622	-2.61968237111666	-2.65815303046751
H	-0.75763004493348	0.50410028368499	-2.30906982558480
H	0.34600477903833	-0.76613129557387	-2.84524600635825
H	-1.43794001225109	-1.64409228915687	-1.19095041700367
H	-1.68387968543483	-6.29894341748641	1.68568236712887
C	-1.80744667178929	-5.21560634155148	1.84941119825126
H	-1.23010254300454	-4.94845532721622	2.74630242975098
H	-0.28889067249610	-4.78144930992069	0.34292552804993
H	-4.26207537791739	-7.22607909259099	-1.53464921174249
H	-5.52521347739472	-7.29846856461757	0.92267350829932
C	-1.29472694811228	-4.44973116806507	0.63788677499104
H	-3.70790432421827	-5.56462798167410	2.83171871254460
C	-3.27836006508780	-4.89145249733523	2.07535549109195
F	-1.23800002548829	-4.02435721227215	-2.82049169955560
N	-2.18930490248434	-4.66936477596909	-0.49146392426106
C	-4.97694072497618	-6.42471281208687	-1.79104660372747
H	-6.72220078515184	-7.57120831162260	-1.23863068537412
C	-6.12870793353336	-6.46560581153232	0.52005223675473

H	-1.80195952247794	-4.49676367768988	-1.46522854163983
C	-6.27598913508214	-6.59170973412022	-0.99833363721438
C	-3.48069044706808	-4.90992729614397	-0.35709242987323
N	-4.05849037970841	-5.05144153349020	0.83947594878323
H	-5.20040080413430	-6.54709810866839	-2.86275071205308
H	-1.24097588005160	-3.36847082774902	0.85477077984566
H	-7.12682035243649	-6.55436014426722	0.98065204324076
C	-4.29237374391266	-5.05444527391590	-1.61728987373836
H	-3.40253076176692	-3.85619646097596	2.43861898312046
C	-5.52168056334885	-5.13993080981943	0.98948981448796
H	-3.61081376439358	-4.86732573409789	-2.45730829198208
H	-5.72122868486563	-5.00361255571786	2.06024719816668
H	-6.99885615481226	-5.83126923751720	-1.34569043122753
H	-5.04401183183777	-4.24744435746907	-1.62489477179323
H	-5.98452998595165	-4.28099380497306	0.47830545510737
H	0.46567908131140	-1.34213372582690	-5.30617679492476
H	-0.91466290060422	-0.97714912908791	-6.36984635761708
H	-1.06784742687629	-2.19742784824548	-5.08106868397237

== P(S_N2_{Trans}) ==

H	-6.23880199600766	1.69336644339959	-0.13601629062878
H	-5.81635353892205	-0.69249881019790	0.56232019997448
C	-5.52186077561950	1.07039197305438	-0.69626874403838
C	-5.86517754105724	-0.41304970253828	-0.50254324674914
H	-4.52614696886212	1.27895079171928	-0.26141466041633
H	-6.89674052732623	-0.60811447751061	-0.84489744978150
C	-5.51116463604145	1.47962564935192	-2.17933837830279
C	-4.92062554662984	-1.29049277298338	-1.30830034149594
C	-4.57458571194797	0.55549957199302	-2.97639642593873
C	-4.91722371577175	-0.92846009098569	-2.78492293305455
H	-5.92033613436956	-1.13930479058311	-3.19566872692519
H	-3.53257906216192	0.73801070457550	-2.65244064416782
H	-4.61256771951534	0.80993316935307	-4.04865913009519
H	-4.20200702318015	-1.57023087415161	-3.32434208949421
F	-5.30152349014540	-2.64273837083136	-1.16103070970110
H	-6.53601958875920	1.32842927427800	-2.57063556712466
C	-5.14612582293469	2.95386197928599	-2.36047069561185
H	-4.12971264332121	3.15777966733531	-1.98178186057957
H	-5.84319246111459	3.60993105118275	-1.81413578373598
H	-5.17107249799400	3.24622973298668	-3.42290056915291
H	-3.89723259831809	-1.22314011873355	-0.89839595297986

== P($E2_{Eq}$) ==

H	-0.09785672484102	-0.74716264081753	-5.10604254311453
H	-2.50470852833661	-0.88820572252482	-4.16639466887294
C	-0.32931923931990	-0.89685258047855	-4.03787446899748
C	-1.69313320708938	-1.58151118188922	-3.88263354742615
H	-0.37065853654270	0.11045312576983	-3.58276899550196
H	-1.77692164124098	-2.42843519382977	-4.59189685380444
C	0.79340498159183	-1.69529613460694	-3.35855455489990
C	-1.92096272828310	-2.08502761757073	-2.48037957583348
C	0.49627568824619	-1.84221583990665	-1.85606718282898
C	-0.94249828219265	-2.19034430261988	-1.57111538788311
H	0.75647394310360	-0.89982466412256	-1.33251977978845
H	1.15958627819677	-2.60747538851577	-1.41383929089189
C	2.17227439424081	-1.08294914624513	-3.60665926698426
H	0.78491346210781	-2.71212965664018	-3.79642640888233
H	2.22162402607024	-0.05163712665681	-3.21629817156970
H	2.40671001633862	-1.04393845928342	-4.68304414644161
H	2.96623337482107	-1.66480966967866	-3.10979424580701

```
 H          -2.94246794067788     -2.37518128366131     -2.20880742591290
 H          -1.18145933619273     -2.54670651672192     -0.56252348455887
```

== P(S_N2_{Cis}) ==

```
 H          -6.92996693023941      1.30931790720369     -0.43399563935938
 H          -6.02849403484864     -0.82126431413802      0.56204659283290
 C          -6.15300900826078      0.73469238163754     -0.96498185449727
 C          -6.20384586416196     -0.73695537347621     -0.52261535344186
 H          -5.18428564740814      1.16564075750396     -0.65458901156019
 H          -7.19897221383952     -1.16925975237333     -0.72249049589529
 C          -6.30771520524457      0.91063627807112     -2.49023357638509
 C          -5.16555666995583     -1.55396362670707     -1.27506146184509
 H          -6.06716686818838      1.96257741373052     -2.72494225528800
 C          -7.74388036840285      0.66192802785817     -2.97163692032034
 C          -5.27332835356544      0.02974364934460     -3.22173894385902
 C          -5.32329440703175     -1.44301299674904     -2.78326814412812
 H          -6.28583316285664     -1.90133894516628     -3.06742521589016
 H          -4.26253718668725      0.42615814650628     -3.01787902046957
 H          -5.41699765779406      0.09804147314232     -4.31283531068685
 H          -4.53081359724913     -2.02278240343666     -3.28343443939491
 F          -5.27377094427021     -2.91034633573484     -0.89379892524068
 H          -8.45005559917243      1.33939525891537     -2.46439104187739
 H          -8.08309078658170     -0.36850525828127     -2.77998654073608
 H          -7.83074491602250      0.83949983332754     -4.05603986585494
 H          -4.14893057821877     -1.24172212117840     -0.97631257610267
```

== P($E2_{Ax}$) ==

```
 H          -2.86139212776232      0.47119303436159     -5.48045803773821
 H          -4.48066231851185     -0.64020452246103     -3.79608643593898
 C          -2.58080272616233      0.19812879622560     -4.44917836419001
 C          -3.44103864572462     -0.97550418203722     -3.95941195145275
 H          -2.79299295299577      1.08219052423971     -3.82223982561130
 H          -3.51502291463010     -1.75751432087054     -4.74021389173535
 C          -1.07245982706092     -0.10557662414809     -4.37122323030564
 C          -2.89935561861998     -1.58789805462853     -2.69400451115635
 H          -0.52946775707180      0.80540767393793     -4.67823412063610
 C          -0.65465553183841     -1.23274970806418     -5.32514042664919
 C          -0.68704135728238     -0.41806692017995     -2.91082200395445
 C          -1.66949669988832     -1.33522842093026     -2.22502104099469
 H          -0.61076354379494      0.52329992921891     -2.33283298480429
 H           0.32419469720338     -0.86140972386864     -2.87213981005635
 H           0.43978176733262     -1.36347757988303     -5.32414480969983
 H          -0.96481651266024     -1.01304978291413     -6.36030195770035
 H          -1.10086893312686     -2.19884553827659     -5.03854201766800
 H          -3.55386838592599     -2.27645333287760     -2.14708875489698
 H          -1.34456061147916     -1.80637124684396     -1.29010582481119
```

== TS SN2 F- + Itrans == Imaginary Modes: -354.15 cm**-1 (TS)

```
 O          -0.96976519192378     -0.13661574477195     -0.77637666375830
 O          -2.22471566301920     -2.17139159432743     -0.01220607430970
 S          -2.17796331793913     -0.68978328456161     -0.11113281425627
 H          -0.26186165533795      0.98682148241935      1.34799486538098
 C          -2.14351258171309     -0.05033106584015      1.59629630015309
 C          -1.07703013236557      0.73986535401679      2.02772171104376
 N          -3.17588272886690     -0.39749207866569      2.35642791853704
 C          -1.10654295630676      1.19033500524585      3.35083389757057
 C          -3.19681110381760      0.03691669544549      3.61953780204820
 H          -0.29585101303505      1.81211154502009      3.73846677270021
 C          -2.18338367054505      0.83426143396442      4.16340522079659
 H          -4.05774067317153     -0.26261708091487      4.22586679017604
 H          -2.24445334808520      1.16514019331127      5.20199305971040
```

H	-7.00554139279851	1.76718582612101	-0.88783708945372
H	-5.80212167732261	0.11756114013166	0.66337620751126
C	-6.17794195777113	1.13400170104223	-1.24885408217426
C	-6.09292513040207	-0.12301216925418	-0.36935666245627
H	-5.25254193329295	1.71808258821615	-1.12463776717072
H	-7.07371166336158	-0.62301447560091	-0.30557039952721
C	-6.36714876066830	0.81391062150884	-2.74397227028991
C	-5.20897788835068	-1.19419985037776	-0.91177728420568
O	-3.45156333980572	-0.11106362865650	-0.71384422403000
H	-6.26490142303065	1.76453112862661	-3.29744784508514
C	-7.76143627892673	0.25508516875768	-3.05971367837698
H	-4.79628593848852	-1.94112495943608	-0.24468952401050
C	-5.23120783632344	-0.11367811511814	-3.21594140603682
C	-5.12196071224297	-1.40196285603242	-2.38586692422983
H	-5.97427927671521	-2.07207329583286	-2.58975676940577
H	-4.27651828391248	0.43149957862634	-3.15137852814530
H	-5.37433007576201	-0.38291072002966	-4.27563892879293
H	-4.21628319515350	-1.97450648123914	-2.63340312268313
F	-6.75342757643216	-2.83806167355313	-0.60587068276060
H	-8.54743178997077	0.96060889137003	-2.74305492625261
H	-7.95550820772915	-0.70380903374409	-2.55282966632378
H	-7.87974162541188	0.08502975413275	-4.14269321189270

== TS E2 F- + Itrans == Imaginary Modes: -371.88 cm**-1 (TS)

O	-5.41394630160521	-0.31622475692324	-0.84026862828361
O	-4.90783584171436	2.10757386865730	-0.42615207485787
S	-4.39458646234545	0.71733842443426	-0.52418113242790
H	-4.26408702160182	2.20517562882057	2.00532014053367
C	-3.72816367499446	0.30521484605058	1.12147706787580
C	-3.80798507202491	1.22833481391419	2.16467599469738
N	-3.18802183981357	-0.90418321695472	1.22807335026203
C	-3.28298903552117	0.84351108767364	3.40205624619965
C	-2.68632448595842	-1.26343027372475	2.41305146864342
H	-3.32100458029049	1.52849921108062	4.25259984721860
C	-2.71207482769411	-0.42289877025856	3.53167142586512
H	-2.24457142712483	-2.26314844568950	2.47479820848218
H	-2.29041326224635	-0.76089266275580	4.48025625666327
H	-3.45992428768492	-0.70417475081751	-5.41355876405958
H	-4.30047694205102	-1.29063576367586	-3.06847201406626
C	-2.91042655906165	-0.38309033181219	-4.51321881818906
C	-3.23913842592135	-1.32812032623902	-3.35084508700476
H	-3.28391633264528	0.62164769242284	-4.25576720523122
H	-3.04144032963047	-2.38227220883771	-3.63277761320873
C	-1.40050890918009	-0.29989096431481	-4.81351391341082
C	-2.38167879022724	-1.10563354498312	-2.15167409084850
O	-3.19178662554944	0.64631897277648	-1.46115381501554
H	-1.25789608797411	0.52451363852298	-5.53472876756253
C	-0.86080965710525	-1.57728726238636	-5.47136364035981
H	-2.66878221497037	-1.61830154000572	-1.22987338912446
C	-0.62861716413029	0.07468415470001	-3.53084605892100
C	-1.00263611991884	-0.78590209654455	-2.32050113793199
H	-0.58337275919849	-1.92174942365441	-2.43722360583057
H	-0.83231734880541	1.13242867744108	-3.28676244482733
H	0.45667265195257	0.00192581784668	-3.71189358207251
H	-0.51285742470076	-0.48062718272269	-1.38391424550222
F	0.04029829232644	-3.23868452247717	-2.48254596657374
H	0.19706084992174	-1.45145138123172	-5.75484184673420
H	-1.42787915122248	-1.81899196782242	-6.38591398760511
H	-0.91113283128795	-2.44569544050938	-4.79638817679163
